# High-precision calculation of the quark–gluon coupling from lattice QCD

**DOI:** 10.1038/s41586-026-10339-4

**Published:** 2026-04-08

**Authors:** Mattia Dalla Brida, Roman Höllwieser, Francesco Knechtli, Tomasz Korzec, Alberto Ramos, Stefan Sint, Rainer Sommer

**Affiliations:** 1 Dipartimento di Fisica, Università di Milano-Bicocca, Milano, Italy; 2 INFN Milano-Bicocca, Milan, Italy; 3 Department of Physics, Bergische Universität Wuppertal, Wuppertal, Germany; 4 Instituto de Física Corpuscular (IFIC), CSIC-Universitat de Valencia, Valencia, Spain; 5 School of Mathematics and Hamilton Mathematics Institute, Trinity College Dublin, Dublin, Ireland; 6 Deutsches Elektronen-Synchrotron DESY, Zeuthen, Germany; 7 Institut für Physik, Humboldt-Universität zu Berlin, Berlin, Germany

**Keywords:** Theoretical particle physics, Phenomenology

## Abstract

The outcomes of modern particle physics experiments, such as proton–proton collisions at the Large Hadron Collider at CERN (European Organization for Nuclear Research), depend crucially on the precise description of the scattering processes in terms of the fundamental forces. Among all the known forces that contribute, the limited understanding of the strong nuclear force is a key source of inaccuracy. At the fundamental level, the strong force is described by quantum chromodynamics, the theory of quarks and gluons. Their coupling, *α*_s_, becomes weaker at high energies (asymptotic freedom), enabling power series expansions in *α*_s_, but the confinement of quarks in hadronic bound states usually requires additional model assumptions. Consequently, determinations of *α*_s_ from experiment mostly remain with large systematic theory errors^1,2^. Here we report the model-free determination of *α*_s_ with unprecedented precision from low-energy experimental input combined with large-scale numerical simulations of the first-principles formulation of quantum chromodynamics on a space–time lattice. The uncertainty, about half that of all other results combined^3^, originates predominantly from the statistical Monte Carlo evaluation and has a clear probabilistic interpretation. The result for *α*_s_ describes both low-energy hadronic physics with the help of lattice quantum chromodynamics and high-energy scattering using the perturbative expansion. By removing a source of theoretical uncertainty, our estimate of *α*_s_ could enable markedly improved analyses of many high-energy experiments^4^. This will contribute to the likelihood that small effects of yet unknown physics are uncovered, as well as enable stringent precision tests of the Standard Model.

## Abstract (web summary)

Large-scale lattice quantum chromodynamics simulations enable a model-free, highly precise determination of the strong coupling constant *α*_s_, reducing theoretical uncertainty and improving precision tests of particle physics.

## Main

At the fundamental level, the strong nuclear force between nucleons arises from quantum chromodynamics (QCD), a quantum field theory formulated in terms of their ‘colour-charged’ elementary constituents, the quarks and gluons. The interaction between these constituents is characterized by being weak at very high energies and short distances, a phenomenon known as asymptotic freedom^5,6^, whereas, in contrast to the other forces, it is so strong at nuclear distances that thinking of quarks and gluons as individual particles makes no sense at all. We speak of ‘confinement’: fundamental quarks and gluons cannot be directly observed, but instead, only composite ‘colour-neutral’ states, such as protons, neutrons or π-mesons, are observed in experiments. This fact poses several challenges, including the fundamental question of how to determine the strength of the interaction between quarks and gluons at high energy.

The quark–gluon coupling, *α*_x_(*μ*), depends on the energy scale, *μ*, of the interaction and also on its detailed definition, summarized as the ‘scheme’, x. Owing to confinement, we cannot collide quarks with quarks or gluons and determine *α*_x_(*μ*), directly in experiments. Instead, phenomenological estimates of the strong coupling are obtained by examining different processes, such as electron–positron or proton–proton collisions at various energy scales. After decades of theoretical and experimental efforts to parameterize the effects of confinement and to identify observables in which these effects are minimized, significant uncertainties persist. In particular, in determining a world average of *α*_x_(*μ*), notably by the Particle Data Group (PDG), different categories still exhibit uncertainties in the range of 1.5–3% (compare ref. ^3^ and Fig. 5). In fact, in most cases, these are not simply due to the limited precision of the experimental data, but include significant systematic uncertainties originating from the lack of an analytic understanding of confinement. In this situation, we cannot profit much from having more experimental data in reducing the uncertainty in *α*_x_(*μ*).

The inaccuracy of *α*_x_(*μ*) limits the potential of current experiments that test the fundamental laws of nature^4^. Even when all phenomenology extractions of the strong coupling are combined, they lead to an error of about 1%. This uncertainty propagates, for example, into a 2–4% uncertainty in the rate of production of Higgs particles by gluon fusion^7^ or its decay into gluons^8^. Furthermore, reducing the current uncertainty in the strong coupling by a factor of 2 turns out to be crucial^9^ for finding out whether the vacuum of the Standard Model is stable^10^ and to constrain extensions of the Standard Model, which cure the possible instability^11,12^.

A first-principles, robust, free of modelling uncertainties determination of the strong coupling avoids the limitations of extractions from experimental data and will affect  ongoing searches for new physics.

**Fig. 1:**
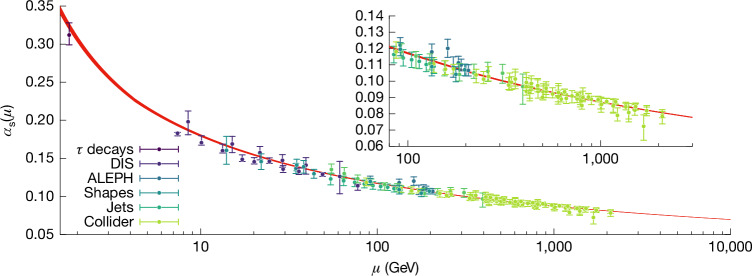
Scale dependence of the strong coupling. The strong coupling for a wide range of energy scales, as determined from our result for *Λ*_QCD_, is represented by the red band. The data points show the experimental determinations from various processes with their uncertainties as quoted by the PDG^3^.

Here we provide such a determination. We analyse the scale dependence of the strong coupling, as described by its *β*-function:

1 $$\mu \frac{{\rm{d}}}{{\rm{d}}\mu }{\alpha }_{{\rm{x}}}(\mu )={\beta }_{{\rm{x}}}({\alpha }_{{\rm{x}}}(\mu )),$$

which has an expansion of the form ${\beta }_{{\rm{x}}}({\alpha }_{{\rm{x}}})=-{\beta }_{0}{\alpha }_{{\rm{x}}}^{2}-{\beta }_{1}{\alpha }_{{\rm{x}}}^{3}+{\rm{O}}({\alpha }_{{\rm{x}}}^{4})$, with leading positive coefficients *β*_0_, *β*_1_, which are independent of the scheme. This implies that *α*_x_(*μ*) runs with the scale *μ*, decreasing with increasing *μ*, with a leading behaviour proportional to 1/ln(*μ*/*Λ*_x_), as shown in Fig. 1. This phenomenon, known as asymptotic freedom^5,6^, implies that perturbative series expansions in powers of the strong coupling become accurate at high energies, as shown by the *β*-function itself. The scheme independence of the leading coefficients, *β*_0_, *β*_1_, implies that the asymptotic scale dependence is universal, and the *Λ-*parameters of different schemes are simply related by exactly calculable constants. Conventionally, we use the modified minimal subtraction scheme^13^ ($\overline{{\rm{M}}{\rm{S}}}$) to quote the coupling ${\alpha }_{{\rm{s}}}\equiv {\alpha }_{\overline{{\rm{M}}{\rm{S}}}}$ and ${\Lambda }_{{\rm{Q}}{\rm{C}}{\rm{D}}}\equiv {\Lambda }_{\overline{{\rm{M}}{\rm{S}}}}$.

Knowledge of *Λ*_QCD_ and the *β*-function is equivalent to knowing the coupling at any given scale *μ*. In the $\overline{{\rm{M}}{\rm{S}}}$ scheme, the expansion coefficients of ${\beta }_{\overline{{\rm{M}}{\rm{S}}}}$ are known up to high order, including *β*_4_, that is, five-loop order^14–18^, so that the scale dependence of ${\alpha }_{\overline{{\rm{M}}S}}(\mu )$ can be accurately predicted down to *μ* of the order of 1 GeV.

In this paper, we determine *Λ*_QCD_ with two independent, dedicated strategies replacing the modelling of confinement by numerical simulations of lattice QCD. Our *α*_s_-uncertainty of about 0.5% is due to the finite computational resources and not due to our limited theoretical understanding. (The total cost of our *α*_s_-dedicated simulations is 400 million core hours). Combined with ${\beta }_{\overline{{\rm{M}}{\rm{S}}}}$, our determination of the coupling can be compared with experimental estimates at various energies, as shown by the data points in Fig. 1. The new, precise result also provides opportunities for better understanding of how confinement manifests itself in different processes and for extracting more detailed information from the experimental data.

### The role of lattice QCD

The modelling of confinement is entirely by-passed in lattice QCD, a genuinely non-perturbative formulation of QCD on a (Euclidean) space–time lattice with spacing *a*. Quark and gluon fields are sampled on the lattice points and edges, respectively. If the space–time volume is finite, the number of QCD degrees of freedom is reduced to a finite albeit large number, enabling the numerical evaluation of observables by large-scale computer simulations. Predictions for hadronic observables, such as the mass of the proton, *m*_p_, or the leptonic decay width of π-mesons, can be obtained for a given choice of the Lagrangian parameters, the bare quark masses and bare coupling *g*_0_. To make contact with the natural world, we need to take the continuum limit, *a* → 0, based on numerical data for a range of *a* values. This is achieved by simulating lattices with decreasing values of the bare coupling, ${g}_{0}^{2}\to 0$ and thus *a* → 0, while the bare quark masses are tuned to match the physical values of the chosen experimental inputs.

In lattice QCD, confinement is a direct consequence of the simulated nonlinear dynamics of QCD, not of some model. Still, conventional lattice QCD determinations of the strong coupling are typically limited by systematic uncertainties. In a volume large enough to accommodate hadrons, the typical momentum cutoff π/*a* is 6−15 GeV. This is one order of magnitude below the universal large energy region, in which low-order perturbation theory is accurate. Together with the basic requirement that physical scales have to be well below the cutoff, *μ* ≪ π/*a*, a large-volume approach to determine the strong coupling would require lattices with significantly more than 100 million lattice points (the current state of the art), along with computational resources several orders of magnitude beyond what is presently available. Instead, most lattice QCD determinations of *α*_x_(*μ*) make compromises, performing the extraction at intermediate energies with estimates of what is the effect on *α*_x_(*μ*). Different strategies exist, as reported in the FLAG (Flavour Lattice Averaging Group) review^19^, with estimated precisions of 1–2%. Similar to the phenomenological situation, these uncertainties are not the result of the limited statistics in the computer simulation, but they are limited by our insufficient analytical control over QCD at low energies. A notable increase in precision can only be reached with a dedicated strategy reaching high energy non-perturbatively.

This strategy, known as step scaling, was suggested more than 30 years ago^20^. Then it was tested in a model with one space dimension. The distinguished idea is to use a scheme for the running coupling, in which the energy scale is given by the size of the simulated world, *μ* = 1/*L*. Small volumes probe the high-energy regime of QCD, whereas large volumes probe low-energy scales. The energy dependence of the coupling is obtained by simulating pairs of lattices with extents *L*/*a* and 2*L*/*a*, and a subsequent continuum extrapolation. This relates the values of the coupling separated by a factor of 2 in scale. By iterating this step scaling *n* times, a scale change of 2^*n*^ is achieved. For QCD with *N*_f_ = 3 flavours, the method was developed and applied^21–24^ over many years. As of today, this result dominates the world average and is the only determination with negligible perturbative uncertainties.

Here, we use this step-scaling approach to not only reach a significant increase in precision but also have better control of the potential remaining systematic effects. We show that the continuum limit is approached smoothly and that the perturbative inclusion of dynamical charm and bottom quark effects is well under control.

But most crucially, we complement the step-scaling approach with the ‘decoupling technique’ described in refs. ^25,26^. In our previous implementation, we had discretization errors linear in *am*_*q*_ removed only at one-loop order. In ref. ^27^, we determined the improvement coefficient non-perturbatively. With this knowledge, we now eliminate the so-far dominating systematic effect. The decoupling strategy is based on the observation that QCD with very heavy quarks can be expanded in the inverse quark mass, and the lowest-order term is the theory without quarks. This observation allows us to relate the QCD coupling and the coupling in a world without quarks.

**Fig. 2:**
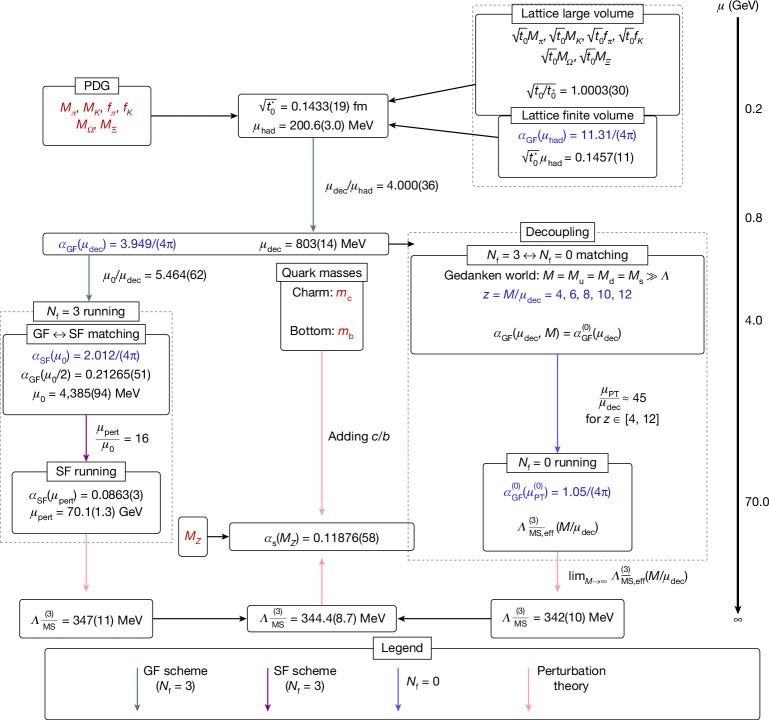
Overview of our computation. We have colour-coded experimental inputs in red and scale definitions in blue. The flowchart follows the energy scale from top to bottom, starting with hadron masses and meson decay constants as input used to set the scale by different collaborations^29–35,43^. This scale is used to reach an energy scale *μ*_dec_. From there on two branches describe our two approaches (*N*_f_ = 3 running and decoupling), to obtain ${\Lambda }_{\overline{{\rm{M}}{\rm{S}}}}^{(3)}$. Our final value, a combination of the result obtained from the two computations and the values of the charm and bottom quark masses^19^, is then used to determine the strong coupling. Vertical, coloured arrows represent the non-perturbative running, characteristic of our strategy, in different finite-volume schemes. Perturbation theory is a key element in our computation, but it is only used at very high energies (above 70 GeV) or to include the effect of the missing charm and bottom quarks (which induces very small perturbative and non-perturbative uncertainties).

Results from lattice simulations come in units of the lattice spacing *a*. To express them in physical (energy) units, the units of the lattice spacing *a* must be established through an experimental input, for example, $a={\widehat{m}}_{{\rm{p}}}/{m}_{{\rm{p}}}^{\exp }$, where ${\widehat{m}}_{{\rm{p}}}$ is the dimensionless proton mass measured in lattice simulations, and ${m}_{{\rm{p}}}^{\exp }$ is the physical, experimentally measured, one. To minimize the uncertainty from the conversion between lattice and physical units, it is common^19^ to introduce an intermediate step, by first relating the experimental input to a technical, not experimentally accessible, (length-) scale, $\sqrt{{t}_{0}}$, derived from the Yang–Mills gradient flow (GF)^28^. Nominally quite precise values $\sqrt{{t}_{0}}$ are available from the literature^29–35^. They differ by the discretization of QCD, and some results include the heavier charm quark in the simulations. They also use various experimental inputs, from baryon octet masses, the Ω-baryon mass, to leptonic decay rates of pion and/or kaon^19^ for the overall scale, whereas the physical quark masses are set by the experimental masses of pions and kaons (Fig. 2, top box).

As discussed in more detail in the Supplementary information, the values of $\sqrt{{t}_{0}}$ differ outside of the quoted error bars. A fit to a common value yields *s* ≡ *χ*^2^/dof = 2.8, where dof indicates the degree of freedom. In these situations, the standard PDG procedure stretches all errors by $\sqrt{s}$. This yields $\sqrt{{t}_{0}}=0.1434(7)\,\mathrm{fm}$. For a safe estimate, we enlarge the error further such that all precise central values are covered. This yields $\sqrt{{t}_{0}}=0.1434{(7)}_{{\rm{s}}{\rm{t}}{\rm{a}}{\rm{t}}}{(17)}_{{\rm{r}}{\rm{o}}{\rm{b}}{\rm{u}}{\rm{s}}{\rm{t}}}{(18)}_{{\rm{t}}{\rm{o}}{\rm{t}}}\,\mathrm{fm}$ (see Extended Data Fig. 3 and the Supplementary information for details). The robust error originates from further enlarging the error from the PDG procedure. It contributes by far the largest systematic uncertainty to our result, but is expected to be reduced significantly by the ongoing simulations and analysis of the community^4,19^.

Apart from *t*_0_, we use other theory-defined scales to split up the computation in a way that yields a very precise result for *α*_s_(*m*_*Z*_). Their definition is based on a common principle. Generic running couplings decrease monotonically with the energy scale (Fig. 1); they are in one-to-one relation with the energy scale. Given a non-perturbatively defined coupling *α*_x_(*μ*), we can then define a scale *μ*_ref_ by specifying a reference value for a coupling, ${\alpha }_{{\rm{x}}}({\mu }_{{\rm{r}}{\rm{e}}{\rm{f}}})\equiv {\alpha }_{{\rm{x}}}^{{\rm{r}}{\rm{e}}{\rm{f}}}$. For convenience, the used theory scales are shown in Fig. 2, which also serves as an orientation about our strategy. It shows how we reach higher and higher energy and finally determine the *Λ-*parameter.

The main split of our computation uses *μ*_dec_ in refs. ^25,26^:

2 $${\Lambda }_{{\rm{Q}}{\rm{C}}{\rm{D}}}\sqrt{{t}_{0}}={\mu }_{{\rm{d}}{\rm{e}}{\rm{c}}}\sqrt{{t}_{0}}\times \frac{{\Lambda }_{{\rm{Q}}{\rm{C}}{\rm{D}}}}{{\mu }_{{\rm{d}}{\rm{e}}{\rm{c}}}}\,.$$

Both dimensionless factors can be computed with high precision. However, the second factor presents a main challenge and dominates the error budget. Therefore, we computed it using two methods with very different systematics: the massless step scaling in *N*_f_ = 3 and the decoupling method.

### Direct approach in *N*_f_ = 3 QCD

We implemented the step-scaling method using two different renormalization schemes for the coupling at low- and high-energy scales, respectively. In the region from hadronic *μ*_had_ = 200 MeV to intermediate scales *μ*_0_ = 4.4 GeV, our finite-volume scheme is based on the Yang–Mills gradient flow^21,28^, and we indicate it with *α*_GF_(*μ*); it is closely related to the low-energy scale $\sqrt{{t}_{0}}$ (for details see the Supplementary information). Altogether, our dataset includes 98 simulations at 10 different volumes *L* in the range 1/*L* ≈ 0.2–4.4 GeV. Compared with refs. ^24,26^, our new analysis includes a very fine lattice spacing, with *a*/*L* = 1/64. This allows us to improve the precision and perform crucial checks on the previous continuum extrapolation. We implicitly define an energy scale, *μ*_dec_, by prescribing the value *α*_GF_(*μ*_dec_) = 3.949/(4π). We then determine ${\mu }_{{\rm{d}}{\rm{e}}{\rm{c}}}\sqrt{{t}_{0}}=0.5831(71)$, which implies *μ*_dec_ = 803(14) MeV. For energies above *μ*_dec_, we combine these results with our previous simulations of the high-energy regime in the SF scheme^24^. These include more than 40 simulations at eight values of the volume *L*, which cover energy scales 1/*L* ≈ 4−140 GeV non-perturbatively. An extensive analysis of the continuum limit together with a detailed exploration of the asymptotic high-energy regime^22,36^ leads to *Λ*_QCD_/*μ*_dec_ = 0.433(11), which translates to our final result for the direct method, *Λ*_QCD_ = 347(11) MeV. Although a further error reduction, especially in the high-energy part, seems feasible, we decided to develop an alternative: the decoupling method. It is computationally more efficient and, even more importantly, affected by very different systematic uncertainties: discretization errors and perturbative errors are very different in *N*_f_ = 3 and the pure gauge theory.

### The decoupling method

The idea is based on the following observation^25^. If we increase the masses of the quarks in a gedanken experiment, eventually the low-lying spectrum of QCD matches the spectrum of the pure gauge theory, in which quarks are absent; we say they are decoupled. In this way, QCD is connected with the pure gauge theory, the theory without any quarks. As the latter is easy to simulate, better precision can be achieved compared with QCD^37^. The exact connection requires the fundamental scale of the pure gauge theory, *Λ*^(0)^, to be adjusted appropriately, ${\Lambda }_{\overline{{\rm{M}}{\rm{S}}}}^{(0)}=P(M/{\Lambda }_{\overline{{\rm{M}}{\rm{S}}}}^{(3)})\,{\Lambda }_{\overline{{\rm{M}}{\rm{S}}}}^{(3)}$, where in ${\Lambda }_{\overline{{\rm{M}}{\rm{S}}}}^{({N}_{{\rm{f}}})}$ the number of quarks, *N*_f_, is indicated. Here and below, *M* refers to the renormalization group invariant mass of the *N*_f_ heavy quarks. The matching factor *P* is known perturbatively to four-loop order^38^ and is routinely being used to relate ${\Lambda }_{\overline{{\rm{M}}{\rm{S}}}}^{(3)}\to {\Lambda }_{\overline{{\rm{M}}{\rm{S}}}}^{(4)}\to {\Lambda }_{\overline{{\rm{M}}{\rm{S}}}}^{(5)}$, across the charm and bottom quark thresholds^39^.

Decoupling works up to *O*(1/*M*^2^) corrections. Detailed studies have shown^38^ that the corrections are small already at masses of the order of the charm quark mass (*M*_c_ ≈ 1.5 GeV). Here we use masses in the range *z* = *M*/*μ*_dec_ = 4−12, where *μ*_dec_ = 803(14) MeV, which translates to *M* ≈ 3−10 GeV, allowing us to explore the approach *M* → ∞ in detail and safely match QCD with the pure gauge theory. Again, we use the GF to define a coupling, now in QCD with three degenerate heavy quarks.

**Fig. 3:**
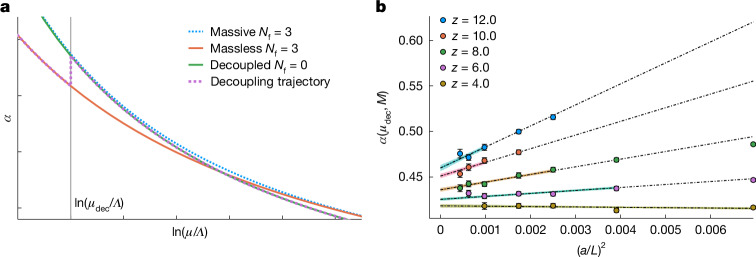
Decoupling of three heavy quarks and continuum extrapolation of massive couplings. **a**, Illustration of the decoupling of three heavy quarks with large mass as described in the model of section 11.2 of the Supplementary information. For energies *μ* ≪ *M*, the massive coupling runs as the pure gauge coupling, whereas for *μ* ≫ *M*, the coupling runs as the massless three-flavour coupling. **b**, Continuum extrapolation of the massive coupling *α*(*μ*, *M*) for *z* = *M*/*μ*_dec_ = 4, 6, 8, 10, 12. The error bands for the different *z* values indicate which data points are included in the fit. Even with the conservative cutoff in the data (*aM*)^2^ < 0.16, the extrapolated continuum values are still very precise.

To illustrate the procedure, the running of this massive coupling *α*_GF_(*μ*, *M*) is shown schematically in Fig. 3 (for a more precise account, see Methods, ‘Decoupling of heavy quarks’). For energies well below their mass, the heavy quarks are decoupled, and *α*_GF_ runs as in pure gauge theory (green line), whereas at *μ* far above their mass, the running is governed by the massless *β*-function; it is slowed down.

Our strategy now follows the magenta trajectory in Fig. 3a. Below *μ*_dec_, we reuse the running in the massless theory. Then, at fixed *μ* = *μ*_dec_, we increase the mass of all three quarks artificially to very high values, eventually to *M* ≈ 10 GeV, following the vertical part of the magenta trajectory. (Note that fixed *μ*_dec_ just means to keep both the bare coupling and the dimensionless *aμ*_dec_ fixed; that is, to work in a mass-independent renormalization scheme.) At the resulting value of the massive coupling, we switch to the pure gauge theory and run to large *μ* where ${\Lambda }_{{\rm{G}}{\rm{F}}}^{(0)}$ is obtained. Converted (exactly) to the $\overline{{\rm{M}}{\rm{S}}}$ scheme, we then use the accurate high-order relation $P(M/{\Lambda }_{\overline{{\rm{M}}{\rm{S}}}}^{(3)})$ between the *Λ-*parameters with and without quarks to revert to ${\Lambda }_{\overline{{\rm{M}}{\rm{S}}}}^{(3)}={\Lambda }_{{\rm{Q}}{\rm{C}}{\rm{D}}}$.

The main challenge is the continuum extrapolation of the massive coupling from our simulation results at finite *a*. On the one hand, the quark mass has to be large for the decoupling approximation to be as accurate as possible. On the other hand, the mass has to be below the momentum cutoff of π/*a* of the lattice. In other words, we need *aM* ≪ 1 and a good understanding of the asymptotic behaviour of discretization effects close to the continuum limit. To this end, we determined an improved discretization^27^ that allows us to completely cancel the dangerous terms linear in *aM*. Next, sufficiently small lattice spacings were simulated, and we performed a combined extrapolation of the results for different quark masses and different lattice spacings. Analysing the theory for discretization effects^40^ in an expansion in 1/*M*, we arrive at an asymptotic form of the discretization errors with only two free parameters. This form fits the data (Fig. 3b) remarkably well. The fit tells us that the coupling changes from *α*_GF_(*μ*_dec_, *M*) = 0.4184(22) for *z* = *M*/*μ*_dec_ = 4 to *α*_GF_(*μ*_dec_, *M*) = 0.4600(41) for *z* = 12 in continuum QCD.

We then matched to the pure gauge theory by equating *α*_GF_(*μ*_dec_, *M*) with the *N*_f_ = 0 coupling. Using previous results in the pure gauge theory from ref. ^37^, we arrive at an estimate of *Λ*_QCD_/*μ*_dec_ at each value of the quark mass. These estimates should all agree up to the mentioned *O*(1/*M*^2^) corrections. The numbers are very close: for quark masses between 5 GeV and 10 GeV, *Λ*_QCD_/*μ*_dec_ varies by 5%. They also follow the expected *c*_0_ + *c*_1_*M*^−2^ behaviour. An extrapolation with this form thus yields our final number *Λ*_QCD_/*μ*_dec_ = 0.426(10) in the three-flavour theory from the decoupling strategy. Together with the value for *μ*_dec_, we get *Λ*_QCD_ = 342(10) MeV. The uncertainty covers the statistical errors and several variations of the functional form used in the continuum, *a* → 0, and decoupling, *M* → ∞, extrapolations. It also includes the uncertainty of the conversion from ${\Lambda }_{\overline{{\rm{M}}{\rm{S}}}}$ of the pure gauge theory to *Λ*_QCD_.

### Final result and concluding remarks

Both the above methods to extract *Λ* have uncertainties dominated by statistics. Theoretical uncertainties, in particular those related to the use of perturbation theory, are subdominant. The systematics are also very different in both methods, and their agreement further corroborates the robustness of our methodology. An average is justified and leads to

3 $${\Lambda }_{{\rm{Q}}{\rm{C}}{\rm{D}}}=344.4(8.7)\,{\rm{M}}{\rm{e}}{\rm{V}}.$$

We still need to account for the missing charm and bottom quarks in our simulations. Their effect is known including high order in the perturbative expansion. A detailed study of both perturbative uncertainties and possible non-perturbative effects is discussed in the Supplementary information. These considerations lead to our final result

4 $${\alpha }_{{\rm{s}}}({m}_{Z})=0.11876(58),$$

where $${\alpha }_{{\rm{s}}}={\alpha }_{\overline{{\rm{M}}{\rm{S}}}}^{({N}_{{\rm{f}}}=5)}$$.

**Fig. 4:**
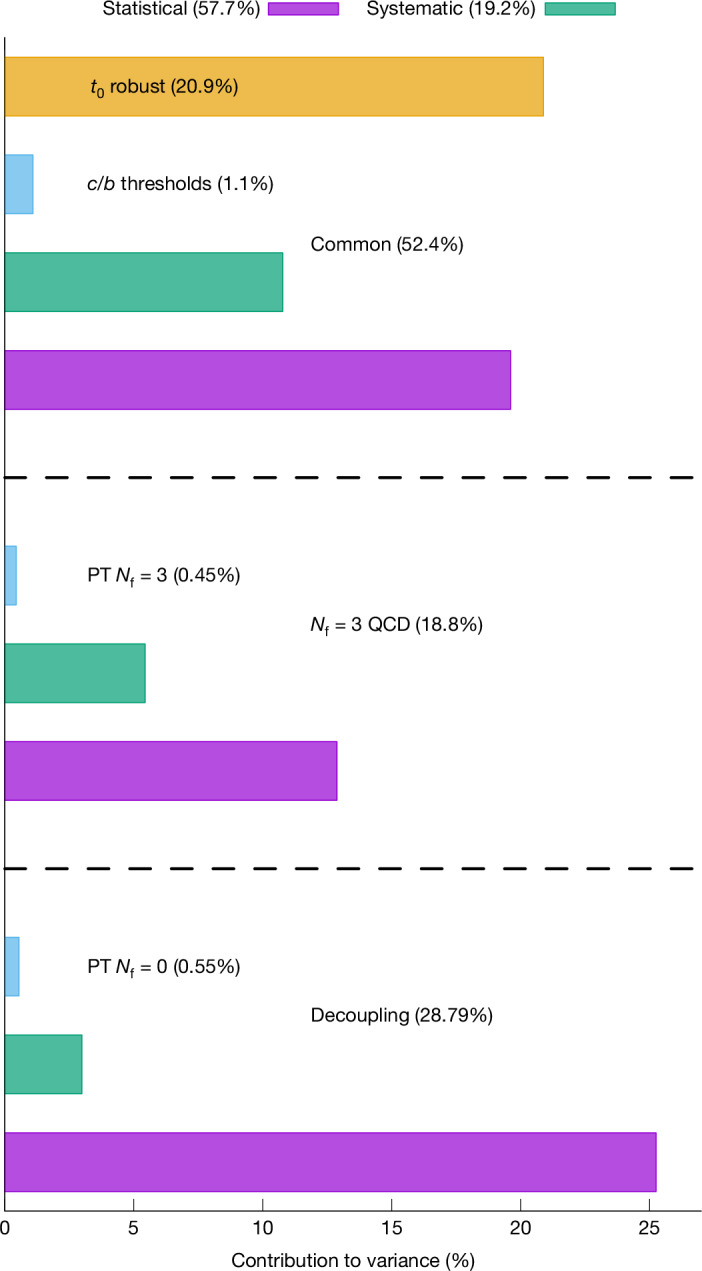
The percentage contributions, ${{\boldsymbol{(}}{{\boldsymbol{(}}{\boldsymbol{\Delta }}{{\boldsymbol{\alpha }}}_{{\bf{s}}}{\boldsymbol{)}}}_{{\boldsymbol{i}}}/{\boldsymbol{\Delta }}{{\boldsymbol{\alpha }}}_{{\bf{s}}}{\boldsymbol{)}}}^{{\bf{2}}}$, originating from error source *i.* The figure splits the different errors in the three main components of our computation: a common component that connects hadronic physics with the intermediate decoupling scale *μ*_dec_, the connection with large energies using *N*_f_ = 3 running and the connection with large energies using the decoupling strategy (see also Fig. 2).

The break-up of the variance, ${(\Delta {\alpha }_{{\rm{s}}})}^{2}$, of *α*_s_ into different contributions is shown in Fig. 4. In both the direct *N*_f_ = 3 QCD and the decoupling approaches, statistical errors dominate by far. Small systematic errors originate from the models used to extrapolate the data to the continuum and a bound on residual linear ${\rm{O}}(a)$ effects (see Extended Data Table 4 and the Supplementary information for details). Perturbation theory enters our computation of *Λ*^(3)^ as well as in including the effect of the charm and bottom quarks, but the effect due to the truncation of the series expansion affects our errors by 2% (Extended Data Table 4). This is a direct consequence of our strategy. Namely, we use perturbation theory at truly weak coupling only (extraction of *Λ*^(3)^) or to very high order combined with very good apparent convergence (charm and bottom thresholds). A special case is the ‘robust’ estimate of the uncertainty in *t*_0_, the only significant non-statistical source of uncertainty. It will be eliminated once scale determinations of different lattice computations agree more closely. It also has a small effect on our final number: dropping it (as in the standard PDG average) would decrease the error in *α*_s_(*M*_*Z*_) only by about 10%.

**Fig. 5:**
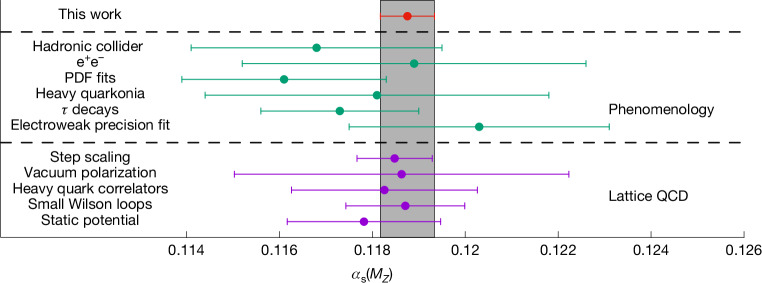
Our determination of *α*_s_(*m*_*Z*_) compared with previous results from the literature. The strong coupling constant *α*_s_ can be determined from a variety of experimental processes, as reviewed by the PDG^3^, and from lattice QCD calculations, as reviewed by FLAG^19^. Our method achieves significantly better precision than the most accurate individual determinations, while maintaining well-controlled systematic uncertainties.

Figure 5 shows our result compared with numbers from other strategies. In comparison with our result, most of them have uncertainties dominated by systematic effects associated with the use of perturbation theory and/or the continuum extrapolation, as quoted by the PDG for the phenomenology results^3^ and by FLAG for lattice results^19^.

An important exception is the category labelled ‘Step scaling’, which uses the methods developed over the years by the ALPHA Collaboration. This result is dominated by our earlier computation^24^, the first robust sub-per cent determination of the strong coupling. Still, as emphasized by one of the PDG reviewers^2^, this computation was based on a single strategy and key systematics (the effect of the heavier charm and bottom quarks and the approach to the continuum limit) required confirmation^24^. Our present computation provides not only this confirmation by simulating finer lattice spacing and with a detailed study of the heavy quarks missing in our simulation, but also a complementary determination based on a new strategy (decoupling of heavy quarks), which also has negligible systematic uncertainties. Moreover, the precision is significantly better than our previous computation, and about two times more precise than all experimental estimates combined.

Beyond the precise number for the strong coupling, there is a qualitative lesson. Recall that QCD is a complicated nonlinear theory with the observed particles completely different from the fundamental quanta in the Lagrangian. Still, surprisingly, we can determine the intrinsic scale *Λ*_QCD_ of the theory and, equivalently, the coupling between quarks and gluons. A conceptual achievement beyond the mere precision of *α*_s_ is that it is determined with experimental low-energy input: the *Ω* baryon mass, together with π, K, D, B meson masses (and decay constants), which are all bound states of quarks and gluons. Figure 1 compares the resulting coupling with phenomenological determinations. Although the latter have some issues, the overall qualitative agreement confirms QCD as the single theory of the strong interactions at all energies, both small and large compared with *Λ*_QCD_. Thus, there is very little room for any modifications or additions to the theory of the strong interactions.

Our precise and first-principles determination of the strong coupling will be key in the quest for new physics at the energy frontier. The analysis of the Higgs boson production and decay at the LHC, the puzzle of the top quark mass or the analysis of the stability of the Standard Model vacuum will immediately and crucially benefit from the increase in precision^4,7–9^. Moreover, the low-energy experimental input used in our methods is uncorrelated with the experimental data of the LHC. Thus, our value for *α*_s_ can be used as input to determine the hadronic parton distribution functions^3,41,42^ relevant for all LHC processes, without having to disentangle correlations between experimental processes and determination of *α*_s_. Being a prediction of QCD, matched to nature at low energy, our value cannot hide or mask new physics effects, a possibility always present when using experimental high-energy data as input.

## Methods

### The scale of QCD and the *Λ-*parameter

We consider QCD with *N*_f_ quark flavours and show how an intrinsic scale can be defined, the QCD *Λ-*parameter. In what follows, we adopt the conventions of our past papers and use the coupling ${\bar{g}}_{{\rm{x}}}(\mu )=\sqrt{4{\rm{\pi }}\,{\alpha }_{{\rm{x}}}(\mu )}$. The *β*-function, as a function of ${\bar{g}}_{{\rm{x}}}$, is given by

5 $$\mu \frac{\partial {\bar{g}}_{{\rm{x}}}(\mu )}{\partial \mu }={\beta }_{{\rm{x}}}({\bar{g}}_{{\rm{x}}}).$$

We emphasize that *β*_x_ is non-perturbatively defined if this is the case for the coupling ${\bar{g}}_{{\rm{x}}}$. As mentioned earlier, the first two coefficients in the expansion *β*_x_(*g*) = −*b*_0_*g*^3^ − *b*_1_*g*^5^ + *O*(*g*^7^),

6 $${b}_{0}=\left(11-\frac{2}{3}{N}_{{\rm{f}}}\right)\times {(4{\rm{\pi }})}^{-2},\quad {b}_{1}=\left(102-\frac{38}{3}{N}_{{\rm{f}}}\right)\times {(4{\rm{\pi }})}^{-4},$$

are renormalization-scheme independent (*β*_*k*_ of equation (1) is proportional to *b*_*k*_, for *k* = 0, 1, …). This asymptotic behaviour for small couplings allows us to integrate the differential equation (5) between the scales *μ* and *μ*′ as follows:

7 $$\frac{{\mu }^{{\prime} }}{\mu }=\exp \mathop{\int}\limits_{{\bar{g}}_{{\rm{x}}}(\mu )}^{{\bar{g}}_{{\rm{x}}}({\mu }^{{\prime} })}\frac{{\rm{d}}g}{{\beta }_{{\rm{x}}}(g)}=\frac{{\varphi }_{{\rm{x}}}({\bar{g}}_{{\rm{x}}}(\mu ))}{{\varphi }_{{\rm{x}}}({\bar{g}}_{{\rm{x}}}({\mu }^{{\prime} }))}\,.$$

Here, the function *φ*_x_ is given by

8 $${\varphi }_{{\rm{x}}}({\bar{g}}_{{\rm{x}}})={({b}_{0}{\bar{g}}_{{\rm{x}}}^{2})}^{-{b}_{1}/(2{b}_{0}^{2})}{{\rm{e}}}^{-1/(2{b}_{0}{\bar{g}}_{{\rm{x}}}^{2})}\times \exp \left\{-\mathop{\int}\limits_{0}^{{\bar{g}}_{{\rm{x}}}}{\rm{d}}g\,\left[\frac{1}{{\beta }_{{\rm{x}}}(g)}+\frac{1}{{b}_{0}{g}^{3}}-\frac{{b}_{1}}{{b}_{0}^{2}g}\right]\right\}.$$

Note that, by design, the integrand in the exponent is regular at zero coupling. Furthermore, the combination $\mu {\varphi }_{{\rm{x}}}({\bar{g}}_{{\rm{x}}}(\mu ))$ is independent of *μ*, has units of energy, and is known as the *Λ-*parameter of QCD:

9 $${\Lambda }_{{\rm{x}}}^{({N}_{{\rm{f}}})}=\mu {\varphi }_{{\rm{x}}}({\bar{g}}_{{\rm{x}}}(\mu ))={\mu }^{{\prime} }{\varphi }_{{\rm{x}}}({\bar{g}}_{{\rm{x}}}({\mu }^{{\prime} })).$$

As these equations are exact, the *Λ-*parameter can be evaluated at any scale *μ*, provided the integral in the exponent of *φ*_x_ can be evaluated reliably. This poses a particular challenge for lattice QCD: the experimental observables used to fix the free parameters, that is, the quark masses and the lattice spacing, are measured at very low energies, where QCD is non-perturbative. Matching to hadronic physics thus requires a hadronic low-energy scale, *μ*_had_. However, the integral extends to zero coupling, that is, an infinitely large energy scale. The idea then is to split the computation into two factors:

10 $$\frac{{\Lambda }_{{\rm{x}}}^{({N}_{{\rm{f}}})}}{{\mu }_{{\rm{h}}{\rm{a}}{\rm{d}}}}=\frac{{\mu }_{{\rm{p}}{\rm{e}}{\rm{r}}{\rm{t}}}}{{\mu }_{{\rm{h}}{\rm{a}}{\rm{d}}}}\times {\varphi }_{{\rm{x}}}({\bar{g}}_{{\rm{x}}}({\mu }_{{\rm{p}}{\rm{e}}{\rm{r}}{\rm{t}}})),$$

where *μ*_pert_ ≫ *μ*_had_ is a large scale, deep in the perturbative regime of QCD. We then use the non-perturbative step-scaling approach (see below) to determine the large-scale ratio (first factor), together with the values of the coupling at both scales. The second factor is evaluated in perturbation theory by inserting the perturbative expansion of the *β*-function, which is, in the $\overline{{\rm{M}}{\rm{S}}}$-scheme, known up to five-loop order.

The *Λ*-parameter is scheme-dependent. Given two mass-independent schemes x and y, with the respective couplings related perturbatively by

11 $${g}_{{\rm{x}}}^{2}={g}_{{\rm{y}}}^{2}+{c}_{{\rm{x}}{\rm{y}}}{g}_{{\rm{y}}}^{4}+\ldots $$

we have the exact relation

12 $${\Lambda }_{{\rm{x}}}/{\Lambda }_{{\rm{y}}}=\exp ({c}_{{\rm{x}}{\rm{y}}}/2{b}_{0}),$$

that is, the scheme dependence is completely encoded in the perturbative one-loop coefficients relating the respective couplings. This provides an indirect non-perturbative meaning for *Λ*-parameters defined in purely perturbative schemes such as the $\overline{{\rm{M}}{\rm{S}}}$ scheme, and ${\Lambda }_{\overline{{\rm{M}}{\rm{S}}}}^{({N}_{{\rm{f}}})}$ is thus taken as the reference scale in QCD. For our particular case, we have

13 $${\Lambda }_{\mathrm{SF}}^{(3)}/{\Lambda }_{\overline{{\rm{M}}{\rm{S}}}}^{(3)}=0.38286(2),$$

with a numerical uncertainty two orders of magnitude below the best current error estimates for ${\Lambda }_{\overline{{\rm{M}}{\rm{S}}}}^{(3)}$.

### Finite-volume renormalization schemes and step scaling

#### Theory

To relate physical observables defined at very different scales by lattice simulations, the key idea is to define an intermediate renormalization scheme in a finite space–time volume, in which the linear extent of the volume, *L*, is used to set the renormalization scale, that is, *μ* = 1/*L*. Given the renormalized coupling in such a finite-volume scheme x, we then proceed with the computation of the so-called step-scaling function (SSF),

14 $${\sigma }_{{\rm{x}}}(u)={\bar{g}}_{{\rm{x}}}^{2}(\mu /2){| }_{{{\bar{g}}_{{\rm{x}}}}^{2}(\mu )=u},$$

which determines the coupling at scale *μ*/2 as a function of the coupling at scale *μ*. The step-scaling function is closely related to the *β*-function

15 $$\mathop{\int}\limits_{\sqrt{{\sigma }_{{\rm{x}}}(u)}}^{\sqrt{u}}\frac{{\rm{d}}g}{{\beta }_{{\rm{x}}}(g)}={\rm{l}}{\rm{n}}\,2.$$

**Extended Data Fig. 1:**
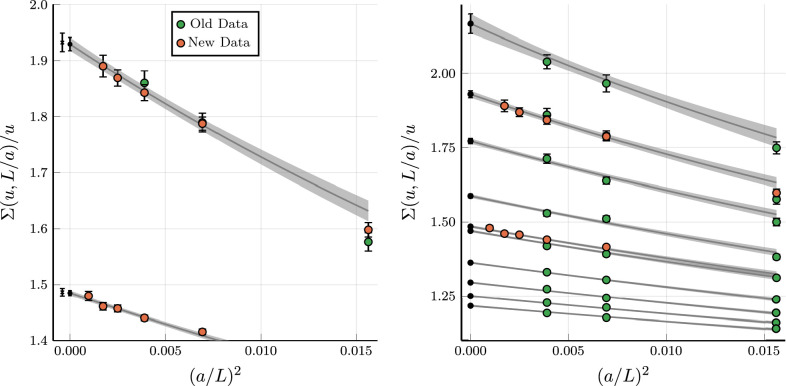
Continuum limit of the step scaling function of the GF coupling. The dataset of Extended Data Table 1 allows for a precise determination of the step scaling function in the continuum limit for *u* ∈ [2.1257, 6.5489]. Left: In particular at two values of the coupling (*u* = 3.9490, 5.8673) we have determined the lattice step scaling function *Σ*(*u*, *L*/*a*) for lattice sizes up to *L*/*a* = 32. The choice *u* = 3.9490 differs from the one in ^23^, while *u* = 5.8673 was also present in the old data. The continuum values are significantly improved in precision (the old continuum values are displayed with a cross; they correspond to the leftmost black points), but central values have barely moved. Right: The full dataset, that includes both the new data and the old data^23^.

Given a finite-volume coupling ${\bar{g}}_{{\rm{x}}}(\mu )$ and its SSF *σ*_x_(*u*) for a range of *u*-values, we obtain the non-perturbative scale evolution of the coupling towards lower energy scales by setting ${u}_{0}={u}_{\min }$ and then iterating

16 $${u}_{k}={\sigma }_{{\rm{x}}}({u}_{k-1}),\qquad k=1,2,3,\ldots $$

until we exit the range of available *u*-values. After 10 steps, we have covered a scale factor of 2^10^, that is, three orders of magnitude. Yet the non-perturbative construction of the SSF can be carried out without ever dealing with large scale differences. All that is required are pairs of lattices of linear dimension *L*/*a* and 2*L*/*a*, in which the measured value of the coupling on the smaller lattice defines the argument *u* and the measured value (at the same bare parameters) on the 2*L*/*a* lattice defines a lattice approximant, *∑*_x_(*u*, *a*/*L*) to *σ*_x_(*u*). Repeating these computations for a range of lattice resolutions, *a*/*L*, then allows us to take the continuum limit

17 $${\sigma }_{{\rm{x}}}(u)={\rm{l}}{\rm{i}}{{\rm{m}}}_{a/L\to 0}{\Sigma }_{{\rm{x}}}(u,a/L)$$

(Extended Data Fig. 1). The procedure is repeated for different values of *u* by adjusting the bare coupling ${g}_{0}^{2}$. Note that equation (15) can be used to obtain the *β*-function. First, we find a suitable parametrization of the *β*-function. Several different choices have been made in the literature, from simple polynomials, inspired by perturbation theory^22^, to Padé ansätze, suitable for a description of data at low and intermediate energy scales^23^. Once the functional form *β*_x_(*x*) is fixed, the set of parameters is determined using a standard *χ*^2^ fit

18 $${\chi }^{2}=\mathop{\sum }\limits_{i=1}^{{N}_{{\rm{p}}{\rm{t}}}}\frac{{\left({\rm{l}}{\rm{n}}2-\mathop{\int}\nolimits_{\sqrt{{\sigma }_{{\rm{x}}}({u}_{i})}}^{\sqrt{{u}_{i}}}\frac{{\rm{d}}g}{{\beta }_{{\rm{x}}}(g)}\right)}^{2}}{{[\beta (\sqrt{{u}_{i}})]}^{-2}{[\delta {u}_{i}]}^{2}+{[\beta (\sqrt{{\sigma }_{{\rm{x}}}({u}_{i})})]}^{-2}{[\delta {\sigma }_{{\rm{x}}}({u}_{i})]}^{2}}.$$

Knowledge of *β*_x_(*g*) then allows us to compute the ratio of the scales associated with two values of the coupling

19 $${\rm{ln}}\frac{{\mu }_{1}}{{\mu }_{2}}=\mathop{\int}\nolimits_{{\bar{g}}_{{\rm{x}}}({\mu }_{1})}^{{\bar{g}}_{{\rm{x}}}({\mu }_{2})}\frac{{\rm{d}}x}{{\beta }_{{\rm{x}}}(x)}\,.$$

Although the step-scaling strategy is straightforward in principle, its practicality depends on several technical details that have been developed over the past 30 years. Perturbation theory in finite volume is non-standard and can be very intricate, depending on the chosen boundary conditions. Using Schrödinger functional (SF) boundary conditions and the standard SF coupling^44,45^, the perturbative conversion to the $\overline{{\rm{M}}{\rm{S}}}$ scheme has been pushed to two-loop order, so that the three-loop *β*-function is known^46^. However, the standard SF coupling is not so well-suited at low energies, because of the increase in its variance for larger physical volumes. Schemes based on the gradient flow (GF) are much better adapted to this regime. A strategy using both couplings and a non-perturbative matching between them at an intermediate scale has been devised for QCD with *N*_f_ = 3 quark flavours in ref. ^24^. We refer to the Supplementary information for the precise definition of the couplings in the SF and GF schemes.

#### Application in this work

As indicated in Fig. 2, these practical issues lead to a further splitting of the ratio *μ*_pert_/*μ*_had_ of equation (12) into two factors:

20 $$\frac{{\mu }_{{\rm{pert}}}}{{\mu }_{{\rm{had}}}}=\frac{{\mu }_{0}}{{\mu }_{{\rm{had}}}}\times \frac{{\mu }_{{\rm{pert}}}}{{\mu }_{0}},$$

where *μ*_0_ denotes the scale at which the GF and SF couplings are matched non-perturbatively. The first factor (that is, the low-energy part) is computed using the GF scheme with SF boundary conditions^21^, whereas the second factor is computed using the SF scheme^44,45^. This strategy combines the advantages of both schemes: the high statistical precision of the GF scheme at low scales and the available analytic control in perturbation theory for the SF scheme.

In detail, the splitting scale, *μ*_0_, is defined through the SF scheme:

21 $${\bar{g}}_{{\rm{S}}{\rm{F}}}^{2}({\mu }_{0})=2.012$$

and non-perturbatively connected to the GF scheme in the form^23^

22 $${\bar{g}}_{{\rm{G}}{\rm{F}}}^{2}({\mu }_{0}/2)=2.6723(64).$$

The second factor in equation (20) is

23 $$\frac{{\mu }_{{\rm{p}}{\rm{e}}{\rm{r}}{\rm{t}}}}{{\mu }_{0}}=16,$$

because we iterate the step-scaling function four times. We arrive at

24 $${\bar{g}}_{{\rm{S}}{\rm{F}}}^{2}(16{\mu }_{0})={\bar{g}}_{{\rm{S}}{\rm{F}}}^{2}({\mu }_{{\rm{p}}{\rm{e}}{\rm{r}}{\rm{t}}})=1.085(4),$$

using the data for the step-scaling function of ref. ^22^ together with the detailed study of the subdominant *O*(*a*) boundary effects in ref. ^36^. At *μ*_pert_, we use perturbation theory in the form equation (8) with the three-loop *β*-function and equation (13) to obtain

25 $$\frac{{\Lambda }_{{\rm{S}}{\rm{F}}}^{(3)}}{{\mu }_{{\rm{p}}{\rm{e}}{\rm{r}}{\rm{t}}}}=0.001894(45),\quad \frac{{\Lambda }_{\overline{{\rm{M}}{\rm{S}}}}^{(3)}}{{\mu }_{{\rm{p}}{\rm{e}}{\rm{r}}{\rm{t}}}}=0.00495(12).$$

The resulting ratio

26 $$\frac{{\Lambda }_{\overline{{\rm{M}}{\rm{S}}}}^{(3)}}{{\mu }_{0}}=0.0792(19)$$

was then also checked to be independent of where the switch to perturbation theory is made, for scales ranging from 2 × *μ*_0_ to 32 × *μ*_0_ (ref. ^36^).

At low energies, the data of ref. ^23^ have been significantly improved with simulations close to the continuum limit (Extended Data Table 1). In this way, precise values of the continuum limit step-scaling function *σ*(*u*) and the *β*-function are obtained. Defining the scale ${\bar{g}}_{{\rm{GF}}}^{2}({\mu }_{{\rm{had}}})=11.31$, we get the ratio

27 $$\frac{{\mu }_{0}}{{\mu }_{{\rm{h}}{\rm{a}}{\rm{d}}}}=21.86(33),$$

so that *μ*_pert_/*μ*_had_ = 16 × 21.86(33) = 349.8(5.3).

What remains to be done is the matching of *μ*_had_ to the experimental input. The scale *μ*_had_ refers to massless QCD, as the coupling is renormalized in the chiral limit. To obtain *μ*_had_ in MeV, we pass through the technical scale $\sqrt{{t}_{0}}$. Using an average of different works (including our own), we obtain $\sqrt{{t}_{0}}=0.1434(18)\mathrm{fm}$, determined using different experimental inputs (mainly meson decay constants and hadron masses). The rather generous error of 1.3% covers all the differences, but remains subdominant in the total error for the *Λ*-parameter.

Relating *μ*_had_ to the technical scale $\sqrt{{t}_{0}}$ is then done by

28 $${\mu }_{{\rm{h}}{\rm{a}}{\rm{d}}}=\frac{1}{\sqrt{{t}_{0}}}\times \sqrt{\frac{{t}_{0}}{{t}_{0}^{\star }}}\times (\sqrt{{t}_{0}^{\star }}\times {\mu }_{{\rm{h}}{\rm{a}}{\rm{d}}})=200.6(3.0)\,{\rm{M}}{\rm{e}}{\rm{V}},$$

where ${t}_{0}^{\star }$ is defined as the value of *t*_0_ in flavour symmetric *N*_f_ = 3 QCD. The exact point is specified by $12{m}_{{\rm{\pi }}}^{2}{t}_{0}^{* }=12{m}_{{\rm{K}}}^{2}{t}_{0}^{* }=1.11$. It is close to the point in parameter space where the up-, down- and strange-quark masses are all equal to the average of their physical masses^47,48^. In this symmetric limit, the masses of pions and kaons are about 420 MeV.

Here we have used refs. ^33,43^, in which $\sqrt{{t}_{0}/{t}_{0}^{\star }}=1.0003(30)$, and ref. ^24^, in which $\sqrt{{t}_{0}^{\star }}\times {\mu }_{{\rm{had}}}=0.1457(11)$.

In summary, the step-scaling procedure has allowed us to connect the scale *μ*_had_ ≈ 200 MeV with *μ*_pert_ ≈ 70, 000 MeV without relying on perturbation theory and to obtain the lambda parameter as cited in the main text.

### Decoupling of heavy quarks

#### Strategy

The decoupling strategy is discussed in detail in refs. ^26,38,25^. Here we briefly summarize the main ingredients.

The basic starting point is the relation between the *Λ*-parameters of the fundamental and the decoupled theories

29 $${\Lambda }_{\overline{{\rm{M}}{\rm{S}}}}^{(0)}/{\Lambda }_{\overline{{\rm{M}}{\rm{S}}}}^{(3)}=P(M/{\Lambda }_{\overline{{\rm{M}}{\rm{S}}}}^{(3)}).$$

The function *P* is known with high precision^38^ from coupling matching at four-loop order and running at five-loop order^14–16,18,49–56^. Its argument, $M/{\Lambda }_{\overline{{\rm{M}}{\rm{S}}}}^{(3)}$, involves the renormalization group invariant quark mass, *M*. The perturbative function *P* enters the master equation

30 $${\rho }_{{\rm{e}}\mathrm{ff}}P(z/{\rho }_{{\rm{e}}\mathrm{ff}})=\frac{{\Lambda }_{\overline{{\rm{M}}{\rm{S}}}}^{(0)}}{{\Lambda }_{{\rm{G}}{\rm{F}}}^{(0)}}{\varphi }_{{\rm{G}}{\rm{F}}}^{(0)}({\bar{g}}_{{\rm{G}}{\rm{F}}}(\mu ,M)),\quad z=M/\mu ,$$

which, for given *μ*, implicitly defines *ρ*_eff_. Moreover, this equation contains the *N*_f_ = 0 function ${\varphi }_{{\rm{GF}}}^{(0)}$ (see equation (8)). The latter is known rather precisely from the analysis in ref. ^37^, typically with a 1% accuracy. In Fig. 3, it is illustrated by the curve labelled ‘decoupled’. Solving the master equation for *ρ*_eff_ and extrapolating in *z* at fixed *μ* removes the *O*(*μ*^2^/*M*^2^) corrections to decoupling. Thus, we expect that

31 $${\rm{l}}{\rm{i}}{{\rm{m}}}_{z\to \infty }{\rho }_{{\rm{e}}\mathrm{ff}}={\Lambda }_{\overline{{\rm{M}}{\rm{S}}}}^{(3)}/\mu .$$

The final ingredient in the strategy is the argument of ${\varphi }_{{\rm{GF}}}^{(0)}$: the massive coupling ${\bar{g}}_{{\rm{GF}}}(\mu ,M)$. In its definition and evaluation, the scale, *μ*, is fixed in the massless theory by ${\bar{g}}_{{\rm{GF}}}(\mu ,0)$. Its value determines numbers for the bare improved coupling ${\mathop{g}\limits^{ \sim }}_{0}^{2}$ for any desired resolution *a*/*L* = *μa*. We then keep ${\mathop{g}\limits^{ \sim }}_{0}^{2},a/L$ fixed when switching to finite mass in lattice units, *aM*. This is the non-perturbative implementation of a mass-independent renormalization scheme^57^.

#### Implementation

In our implementation of the strategy, we take some small detours to achieve improved precision. In particular, the massive coupling in the *N*_f_ = 3 theory is defined as ${\bar{g}}_{{\rm{GFT}}}(\mu ,M)$, where the time extent is enlarged, *T* = 2*L*, to suppress boundary effects, while ${\bar{g}}_{{\rm{GF}}}({\mu }_{{\rm{dec}}},0)=3.494$ is kept fixed. In the *N*_f_ = 0 theory, we then switch back from GFT to GF coupling using ${\bar{g}}_{{\rm{GF}}}^{(0)}({\mu }_{{\rm{d}}ec})=\chi ({\bar{g}}_{{\rm{GFT}}}({\mu }_{{\rm{dec}}},M))$ with a non-perturbative scheme change described by *χ*. Our numerical implementation also determines the product $\frac{{\Lambda }_{\overline{{\rm{M}}{\rm{S}}}}^{(0)}}{{\Lambda }_{{\rm{G}}{\rm{F}}}^{(0)}}\times {\varphi }_{{\rm{G}}{\rm{F}}}^{-1}$ through a non-perturbative switch to the SF scheme at a value of the coupling ${\bar{g}}_{{\rm{GF}}}^{(0)}({\mu }_{{\rm{P}}T})=1.05$, combined with the ratio $\frac{{\Lambda }_{\overline{{\rm{M}}{\rm{S}}}}^{(0)}}{{\Lambda }_{{\rm{S}}{\rm{F}}}^{(0)}}$. We refer to ref. ^26^ for further details.

We vary *z* from 4 to 12, and for each *z* we extract

32 $${\rho }_{{\rm{e}}\mathrm{ff}}={\Lambda }_{\overline{{\rm{M}}{\rm{S}}},{\rm{e}}\mathrm{ff}}^{(3)}(z)/{\mu }_{{\rm{d}}{\rm{e}}{\rm{c}}}={\Lambda }_{\overline{{\rm{M}}{\rm{S}}}}^{(3)}/{\mu }_{{\rm{d}}{\rm{e}}{\rm{c}}}+O(1/{z}^{2})$$

as the numerical solution of equation (30). Combined with the already known *μ*_dec_, we obtain estimates for ${\Lambda }_{\overline{{\rm{M}}{\rm{S}}},{\rm{e}}\mathrm{ff}}^{(3)}$.

The values for the massive coupling, ${\bar{g}}_{{\rm{GFT}}}({\mu }_{{\rm{dec}}},M)$, where we evaluate ${\varphi }_{{\rm{GF}}}^{(0)}(\chi ({\bar{g}}_{{\rm{GFT}}}))$, are determined by a careful continuum extrapolation. First, as discussed before, it is crucial to remove linear effects of the form *aM* (ref. ^27^). Second, we used effective field theory (EFT) to derive the leading terms of the expansion of the lattice discretized observable, namely, ${\bar{g}}_{{\rm{GFT}}}$, in *a* and 1/*M* in the region *aM* ≪ 1 and *z* ≫ 1. The EFTs are the Symanzik effective theory as well as the decoupling expansion, that is, the pure gauge theory with perturbations by dimension six operators. The latter provides corrections to the infinite *M* limit. These EFTs have to be applied in the correct order: the decoupling expansion is applied to the Symanzik effective theory. The crucial information obtained from the EFT analysis is that for sufficiently small *aμ*_dec_, *aM* and large *z*, results at finite lattice spacing are described by

33 $${\bar{g}}_{{\rm{G}}{\rm{F}}{\rm{T}}}^{2}({\mu }_{{\rm{d}}{\rm{e}}{\rm{c}}},M,a)={\bar{g}}_{{\rm{G}}{\rm{F}}{\rm{T}}}^{2}({\mu }_{{\rm{d}}{\rm{e}}{\rm{c}}},M)+{p}_{1}{[{\alpha }_{{\rm{s}}}({a}^{-1})]}^{\hat{\Gamma }}{a}^{2}{\mu }_{{\rm{d}}{\rm{e}}{\rm{c}}}^{2}+{p}_{2}{[{\alpha }_{{\rm{s}}}({a}^{-1})]}^{{\hat{\Gamma }}^{{\prime} }}{(aM)}^{2}.$$

The *a*^2^ terms are accompanied by ${\alpha }_{{\rm{s}}}^{\hat{\Gamma }}(1/a)$ terms, which vary logarithmically with the argument. However, the leading powers $\hat{\Gamma },\,\hat{\Gamma }{\prime}$ are small^40^. Our explicit tests with the available data show that including them or neglecting them has a minor effect, and these variations of the analysis are part of our error budget.

Numerically, the functional form (33) describes the data very well (Extended Data Fig. 4) for quite a large range of ${(a{\mu }_{{\rm{dec}}})}^{2}$ and all *z* ≥ 4. To obtain our continuum results ${\bar{g}}_{{\rm{GFT}}}^{2}({\mu }_{{\rm{dec}}},M)$, we impose a cut of (*aM*)^2^ < 0.16, but the curves in Fig. 3 do extend to larger values and show that the exact position of the cut is not relevant. The numerical analysis also shows no evidence of higher powers in *a* or the mentioned logarithmic corrections. In summary, the continuum values are solid.

**Extended Data Fig. 2:**
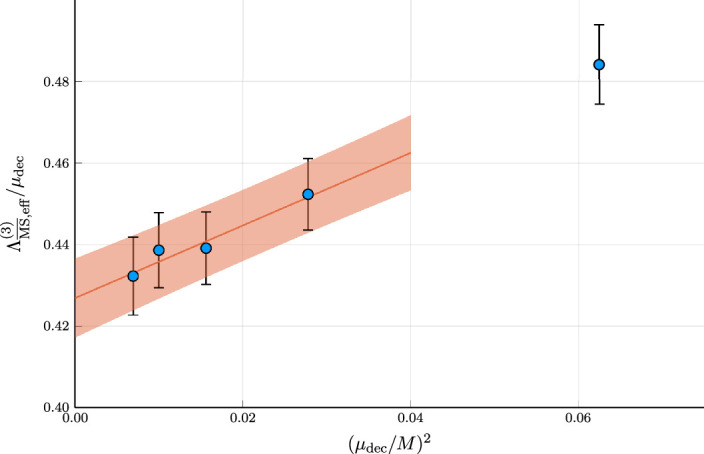
Large mass extrapolation of the lambda parameter from the decoupling strategy. In the decoupling approach the intrinsic scale of QCD is extracted after matching *N*_f_ = 3 QCD and the pure gauge theory using three degenerate heavy quarks of mass *M*. In the limit *M* → ∞ the decoupling relation becomes exact. We performed simulations with quark masses in the range *M* ≈ 3 − 10 GeV. This plot shows that the approach to infinite mass (with corrections O(1/*M*^2^)) agrees with the expectations of an analysis based on effective field theory. The *M* → ∞ limit is obtained considering only masses *M* ≳ 5 GeV.

**Extended Data Fig. 3:**
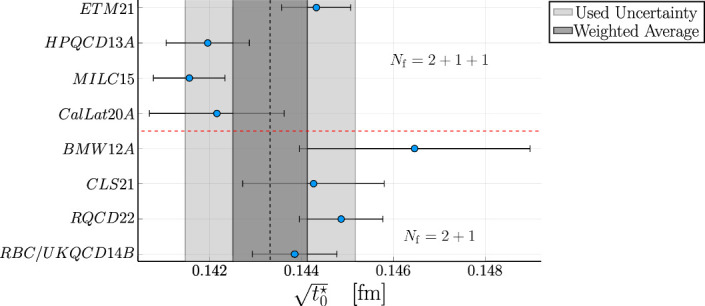
The length scale $\sqrt{{{\boldsymbol{t}}}_{{\bf{0}}}^{{\boldsymbol{\star }}}}$ in fm using the ratio $\sqrt{{{\boldsymbol{t}}}_{{\bf{0}}}/{{\boldsymbol{t}}}_{{\bf{0}}}^{{\boldsymbol{\star }}}}$ and the results for $\sqrt{{{\boldsymbol{t}}}_{{\bf{0}}}}$ from different collaborations^29–35,43^. Our conservative average includes the central values of all precise results, and has an uncertainty almost two times larger than a weighted average. It thus takes the presently poor agreement into account.

**Extended Data Fig. 4:**
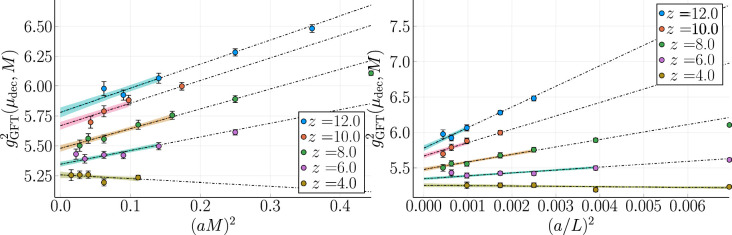
Continuum extrapolation of our data for the massive coupling. Our data, after being corrected with the non-perturbative value of *b*_*g*_, agrees with expectations derived from an effective field theory analysis: most cutoff effects are proportional to (*aM*)^2^, with a constant slope.

**Extended Data Fig. 5:**
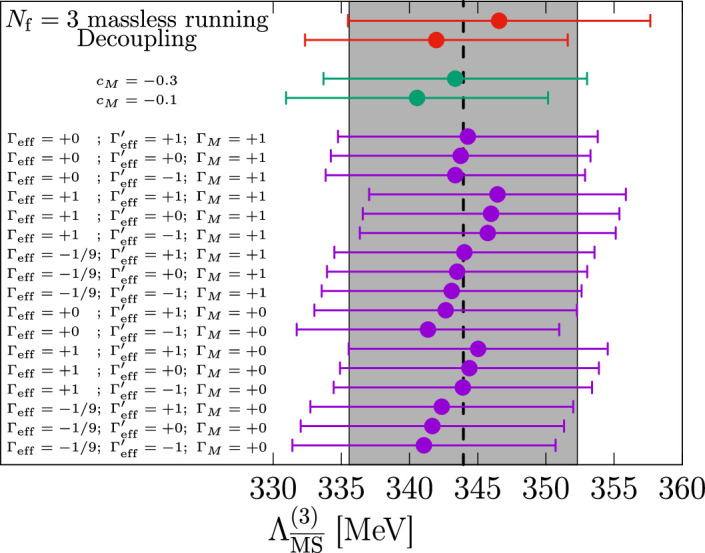
Results for the lambda parameter and its dependence on extrapolation parameters. The two main results, using the massless running or the decoupling strategy, are displayed in red. Our final result (a weighted average of the previous two results) is displayed by vertical grey band. Our result for ${\Lambda }_{\overline{{\rm{M}}{\rm{S}}}}^{(3)}$ depends very little on the logarithmic corrections to the continuum extrapolation of the massive coupling as well as on those in the large-mass extrapolation; these are all displayed by the purple points. The dependence on the linear O(*aM*) shifts in *Δb*_g_ (green points) is even smaller.

All that is left is to take the limit *z* → ∞ of the estimates ${\Lambda }_{\overline{{\rm{M}}{\rm{S}}},{\rm{e}}\mathrm{ff}}^{(3)}(z)/{\mu }_{{\rm{d}}{\rm{e}}{\rm{c}}}$ (Extended Data Fig. 2 and Extended Data Table 3). The extrapolation turns out to be smooth, with the data with *z* ≥ 8 being compatible with the *M* = ∞ value. All data with *z* ≥ 4 are well described by a linear functional form in 1/*z*^2^. Logarithmic corrections to this linear functional form have a very small effect in the final determination ${\Lambda }_{\overline{{\rm{M}}{\rm{S}}}}^{(3)}/{\mu }_{{\rm{d}}{\rm{e}}{\rm{c}}}$ (Extended Data Fig. 5).

We note that our definition of ${\bar{g}}_{{\rm{GFT}}}$ takes place in a finite volume with boundaries in time (as opposed to periodic). In principle, this feature allows for linear corrections in *a* and 1/*M*. The associated boundary terms in the EFTs are rather simple, and we computed their effects. In our setup with *T* = 2*L*, they are well below the statistical uncertainties of ${\bar{g}}_{{\rm{GFT}}}^{2}({\mu }_{{\rm{dec}}},M)$. Note that the uncertainty in the massive coupling contributes a small amount to the total uncertainty in *α*_s_.

## Online content

Any methods, additional references, Nature Portfolio reporting summaries, source data, extended data, supplementary information, acknowledgements, peer review information; details of author contributions and competing interests; and statements of data and code availability are available at 10.1038/s41586-026-10339-4.

## Supplementary information

Supplementary Information This file contains Supplementary Information sections 1–11, including Supplementary Figs. 1 and 2 and Supplementary Tables 1–5.

## Figures and Tables

**Extended Data Table 1:**
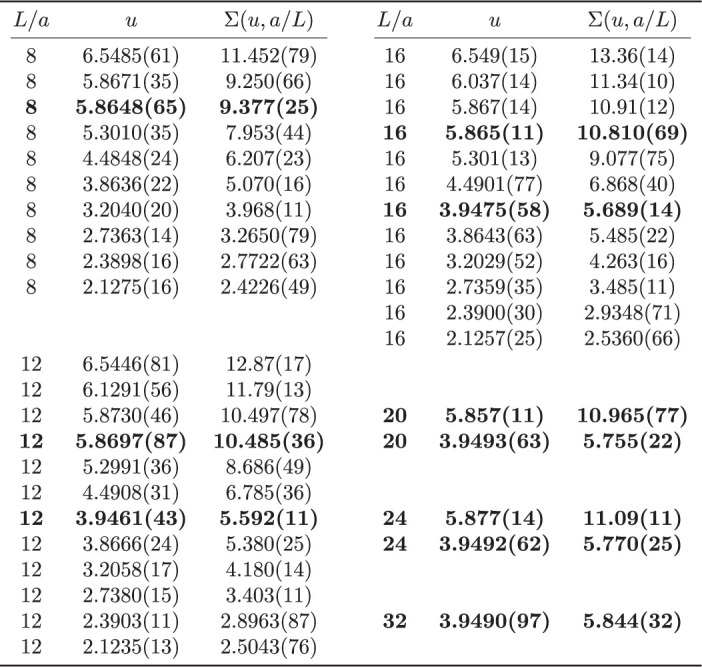
Data used to determine the *β*-function in *N*_f_ = 3 QCD

The columns labeled *u* represent the values of the coupling ${\bar{g}}_{{\rm{GF}}}^{2}(\mu ,a/L)$ at scale *μ* = 1/*L* on a lattice of size *L*/*a*, while the columns labeled *Σ*(*u*, *a*/*L*) list the couplings on a lattice of size 2*L*/*a* with identical bare coupling and thus identical *a*. Data in bold represent the new computations on larger lattices that require simulations of sizes up to 2*L*/*a* = 64 for the determination of *Σ*(*u*, *a*/*L*) for *L*/*a* = 32. This dataset allows for a precise and accurate continuum extrapolation of the step scaling function (see Extended Data Fig. 1).

**Extended Data Table 2:**
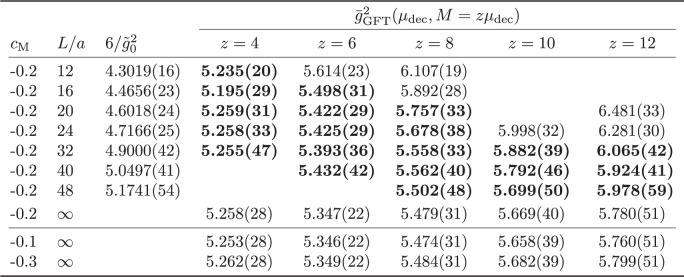
Massive couplings ${{\bar{{\boldsymbol{g}}}}^{{\bf{2}}}}_{{\bf{GFT}}}{\boldsymbol{(}}{{\boldsymbol{\mu }}}_{{\bf{dec}}},{\boldsymbol{M}}{\boldsymbol{)}}$ at finite *L*/*a* for different values of *z* = *M*/*μ*_dec_ corrected to the non-perturbative *b*_g_ value and parameter *c*_M_ = −0.2 (cf. Supplementary information, Section 6.1)

In the last three rows we list the continuum limits of the couplings also considering *c*_M_ = − 0.1, − 0.3. The reader can see that the effect of the parameter *c*_M_ is not significant in the values of the coupling in the continuum. The continuum limits are computed from a global ansatz (Supplementary information, Section 6.1) with ${\Gamma }_{{\rm{e}}\mathrm{ff}}=\Gamma {\prime} =0$ using only the data highlighted in bold face. These satisfy the cut (*aM*)^2^≤0.16. Different choices of ${\Gamma }_{{\rm{e}}\mathrm{ff}},\Gamma {\prime}$ are considered later (cf. Extended Data Fig. 5).

**Extended Data Table 3:**
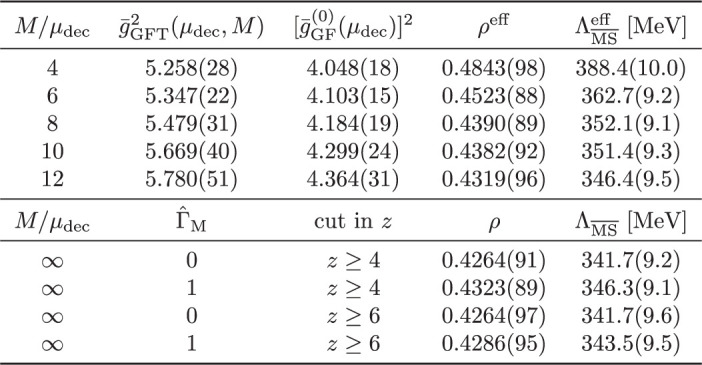
Values of the massive coupling in the continuum

The massive GFT coupling (*c *= 0.36, *T *= 2*L*) in the continuum and the corresponding values of the GF coupling in the pure gauge theory (*c *= 0.3, *T *= *L)* are given. We also quote the effective Λ-parameter both in units of the decoupling scale *μ*_dec_ and in physical units. Finally we also quote the extrapolations *M* → ∞ of the latter. The couplings themselves diverge logarithmically in this limit.

**Extended Data Table 4:**
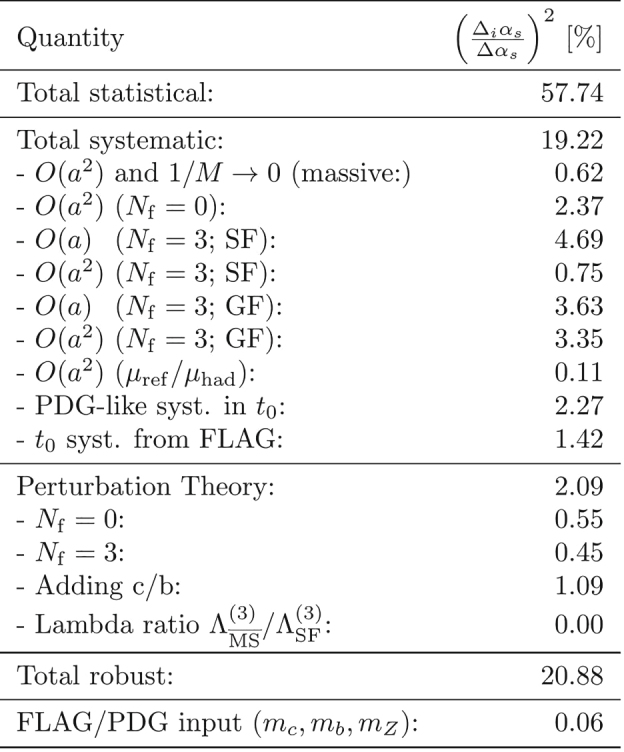
Complete breakdown of the per-cent contributions ${{\boldsymbol{(}}{{\boldsymbol{(}}{\boldsymbol{\Delta }}{{\boldsymbol{\alpha }}}_{{\boldsymbol{s}}}{\boldsymbol{)}}}_{{\boldsymbol{i}}}/{\boldsymbol{\Delta }}{{\boldsymbol{\alpha }}}_{{\boldsymbol{s}}}{\boldsymbol{)}}}^{{\bf{2}}}$ to the total uncertainty in *α*_*s*_(*M*_*Z*_), itemized by error source *i*

The statistical uncertainty dominates the budget; individual systematic terms contribute a small amont to the squared error, except for our robust scale uncertainty (see Supplementary information, Section 3, for a detailed discussion). Uncertainties tied to perturbation theory account for a negligible  ≈ 2% of the squared error.

## Data Availability

We have made a replication package^58^ publicly available at https://igit.ific.uv.es/alramos/replication-package-2501.06633. This package contains a reduced dataset from our original simulations that is nevertheless enough to reproduce the analysis and final numbers quoted in this paper. The dataset can be found in the different files under the folder data. Data analysis is based on the so-called Γ-method^59^, using automatic differentiation for error propagation as suggested in ref. ^60^. All data are stored in the open format BDIO (http://bdio.org/). We have developed free software libraries to read and write in this format in different languages: C*,* https://github.com/to-ko/bdio; Julia, https://igit.ific.uv.es/alramos/bdio.jl; and ALPHAio.jl, https://igit.ific.uv.es/alramos/alphaio. All data of the replication package^58^ are stored using this format.

## Code availability

Configurations and measurements were produced with a custom version of the open source code openQCDv1.6 (https://luscher.web.cern.ch/luscher/openQCD/index.html). Our modified version is publicly available at https://igit.ific.uv.es/alramos/openqcdv1.6 (branch gbar-massive). Example input files (in particular those used to determine the massive coupling in Extended Data Table 2) can be found under the folder infiles. Code to reproduce the analysis of the paper can be found in our replication package^58^.
