# Supplementary material for: High-precision calculation of the quark–gluon coupling from lattice QCD
Source: Nature. 2026 Apr 8;652(8109):328–34. doi: 10.1038/s41586-026-10339-4 (PMC13061608; doi:10.1038/s41586-026-10339-4)
Supplement: Supplementary file 1 — This file contains Supplementary Information sections 1–11, including Supplementary Figs. 1 and 2 and Supplementary Tables 1–5. [file 41586_2026_10339_MOESM1_ESM.pdf]

nature portfolio

https://doi.org/10.1038/s41586-026-10339-4

## Supplementary information

# High-precision calculation of the quark–gluon coupling from lattice QCD

In the format provided by the
authors and unedited

# Supplementary material for: High-precision calculation of the Quark–Gluon coupling from Lattice QCD

(ALPHA collaboration)

Mattia Dalla Brida[1,2], Roman Höllwieser[3], Francesco Knechtli[3], Tomasz Korzec[3], Alberto Ramos[4*], Stefan Sint[5], Rainer Sommer[6,7]

[1]Dipartimento di Fisica, Università di Milano-Bicocca, Piazza della Scienza 3, I-20126 Milano, Italy.
[2]INFN Milano-Bicocca, Piazza della Scienza 3, Milan, I-20126, Italy.
[3]Department of Physics, Bergische Universität Wuppertal, Gaußstr. 20, 42119 Wuppertal, Germany.
[4]Instituto de Física Corpuscular (IFIC), CSIC-Universitat de Valencia, 46071, Valencia, Spain.
[5]School of Mathematics and Hamilton Mathematics Institute, Trinity College Dublin, Dublin 2, Ireland.
[6]Deutsches Elektronen-Synchrotron DESY, Platanenallee 6, 15738 Zeuthen, Germany.
[7]Institut für Physik, Humboldt-Universität zu Berlin, Newtonstr. 15, 12489 Berlin, Germany.

*Corresponding author(s). E-mail(s): alberto.ramos@ific.uv.es;
Contributing authors: mattia.dallabrida@mib.infn.it; roman.hoellwieser@gmail.com; knechtli@uni-wuppertal.de; korzec@uni-wuppertal.de; Sint@maths.tcd.ie; Rainer.Sommer@desy.de;

## Contents


**1 On the estimation of uncertainties** **2**
1.1 Systematic errors . . . 4

**2 Observables** **5**
2.1 Gradient flow . . . 5
2.2 Schrödinger functional . . . 6
2.3 Gradient-flow couplings . . . 9

**3 Scale setting** **11**

**4 The running coupling at low energies** **12**

**5 The strong coupling from $N_\mathrm{f} = 3$ QCD** **14**

**6 Decoupling of heavy quarks** **15**
6.1 Continuum limit of the massive couplings . . . 17
6.2 The determination of $\Lambda^{(3)}_{\overline{\mathrm{MS}}}$ . . . 20

**7 Charm and bottom contributions** **22**
7.1 Perturbative truncation errors . . . 22
7.2 Non-perturbative *charm* contributions . . . 25

**8 $\Lambda$ parameters and the strong coupling** **26**

**9 Mass renormalization** **28**

**10 Boundary contributions to the decoupling limit** **33**
10.1 Effective decoupling theory and $\mathrm{O}(1/M)$ terms . . . 33
10.2 Leading order estimate of the $\mathrm{O}(1/M)$ corrections . . . 34

**11 Massive gradient flow coupling in perturbation theory** **36**
11.1 One-loop GF coupling in infinite volume . . . 36
11.2 Renormalization group improved coupling in the one-loop model . . . 37
11.3 Leading $\mathrm{O}(1/z^2)$ corrections to the decoupling relation in the model . 38
11.4 Illustration . . . 40


# 1 On the estimation of uncertainties

Our results are subject to statistical and systematic uncertainties, inherent to lattice QCD computations. Statistical uncertainties originate in the stochastic nature of the Monte Carlo sampling methodology that underlies Lattice QCD. These uncertainties are well understood, and we use the $\Gamma$-method to estimate them [2, 3].

Systematic uncertainties originate from the different models used to extrapolate/interpolate our data, and from the use of perturbation theory. There is no single method to estimate these uncertainties. Here we distinguish between two situations:

*Small systematics:* In this case the different models produce different results that deviate one from each other by an amount that is smaller than the statistical fluctuations. In our analysis chain this is, with one exception, the situation for all our systematic effects. In these cases we just use the standard deviation of the central values produced by different models as an estimate of the associated systematic uncertainty. Being precise, if $Q_\alpha$ ($\alpha = 1, \ldots, M$) are the results for a quantity using $M$ different models, we use

$$\Delta_{\rm sys} = \sqrt{\frac{1}{M-1}\sum_{\alpha=1}^{M}[Q_\alpha - \bar{Q}]^2}; \quad \left(\bar{Q} = \sum_{\alpha=1}^{M} Q_\alpha\right), \tag{1}$$

as estimate of the systematic error. In some cases we just compare two different models. In this case the previous formula reduces to taking the absolute value of the difference as an estimate of the systematic.

*Significant systematic:* In certain instances, specific models and/or datasets exhibit deviations that exceed statistical fluctuations. The scale $t_0$ (see Section 3 for details) presents a case where results from various collaborations display tension, being the only case where we find this tension. The literature proposes multiple methods to address such discrepancies, often involving the assignment of different weights to distinct models based on *p-values* and/or *information criteria*. For averages, such as that of $t_0$, the standard procedure employed by the PDG and FLAG involves increasing the uncertainty utilizing the $\chi^2/\text{dof}$. In this work, we adopt a more conservative strategy, supplementing our analysis with a *robust* estimate that encompasses the central values of all precise computations. Specifically, if the contribution of a particular data point to the $\chi^2$ exceeds 2, the *robust* uncertainty is inflated to guarantee that the central value of that specific result falls within the total error band.

Our procedure to deal with the case of significant systematics is very conservative. For the case of $t_0$ the *robust* error estimate contributes a 90% to the final uncertainty in $t_0$, and is four times larger than the usual PDG/FLAG uncertainty.

Both statistical and systematic uncertainties are propagated using the techniques described in [3–5]. This ensures that the correlations between the data are properly taken into account. All numbers in sections 3–8 of this supplementary material, where our main results are obtained, will include an error budget[1]. For some quantity $A$ with central value $v$ we use the format:

$$A = v(STAT)_{\rm stat}(SYS)_{\rm sys}(ROB)_{\rm robust}(TOT)_{\rm total}\,. \tag{2}$$

The uncertainties are denoted as follows: $STAT$ represents the statistical uncertainties, $SYS$ represents the sum of systematic uncertainties, $ROB$ represents the propagation of the robust uncertainty used for $t_0$, and $TOT$ represents the total error on the

[1] The technical sections, namely Sections 9 to 11, do not incorporate an error budget, as the quantities presented therein yield a negligible contribution to the uncertainty in $\alpha_s$ and are predominantly subject to statistical errors.

quantity. Note that for dimensionless quantities, the robust error is often zero, in which case the corresponding value will be omitted.

## 1.1 Systematic errors

According to the previous discussion, we only need to specify the different models that enter in the estimates of the systematic in the following sections. These are listed below, but the reader is encouraged to only read this detailed information after having understood the details of sections 3 to 8.

*Scale setting (section 3):* We use the weighted average of the results of different collaborations to quote a final value for $t_0$. As mentioned above, the different results show some tension, so we add the usual PDG inflation of the error as an additional systematic. On top of that we add our robust estimate, that in the end, accounts for a 90% of the error squared in $t_0$.

*The running coupling at low energies (section 4):* Our usual analysis to determine the continuum extrapolation of the step scaling function already includes an additional weight that reduces the weight of the coarser lattices. On top of this, we estimate the systematic in the continuum extrapolation by taking the difference between including or excluding the coarsest lattice.

*The strong coupling from $N_\mathrm{f} = 3$ QCD (section 5):* The result for $\Lambda^{(3)}_{\overline{\mathrm{MS}}}/\mu_0$ includes a systematic coming from matching with perturbation theory. This is estimated by taking the difference of matching with perturbation theory at approximately 70 GeV and a factor two larger (i.e. approximately 140 GeV). The systematic associated with the continuum extrapolation is estimated by using the different models [6] (see table 6 of the cited reference).

*Decoupling of heavy quarks (section 6):* In this case we have to perform the continuum extrapolation of the massive couplings (see equation (65)), and also take the limit $M \to \infty$ (see equation (67)). The systematic on the quantity $\Lambda^{(3)}_{\overline{\mathrm{MS}}}/\mu_\mathrm{dec}$ is estimated by using different logarithmic corrections (see [1, Extended Data Figure 5]) according to the effective field theory analysis [24]. The decoupling approach uses $N_\mathrm{f} = 0$ results of [8]. Perturbative errors in the matching with the $\overline{\mathrm{MS}}$ scheme are determined by computing the variance of the results after matching with perturbation theory for values $1.0 < g^2 < 1.44$ in the step scaling approach (see figure 15 of [8]). The systematic in the continuum extrapolation are determined using as models different numbers of parameters used to determine the beta function (this includes also variations on the number of parameters used to describe the cutoff effects). We also consider including or dropping the $L/a = 10$ lattices in our analysis.

In general terms the $N_\mathrm{f} = 0$ systematic are very small, and our estimates are very generous. Note that in this pure gauge theory study, very fine lattice spacings are reached, and also very small values of the renormalized coupling. It is worth pointing that the results are completely compatible with the exploration of the different systematic detailed in [9].

*Charm and bottom contributions (section 7):* The section contains detailed information on how the perturbative and non-perturbative systematic are studied.

## 2 Observables

For the reader's convenience, in this section we provide the definitions of the primary observables employed in this work. For clarity of presentation, we introduce them in the continuum framework, while referring the reader to the literature for the details regarding their lattice formulation.

### 2.1 Gradient flow

The Yang-Mills gradient flow (GF) is a powerful tool to probe the dynamics of QCD non-perturbatively [10]. The flow field $B_\mu(t,x)$ of SU(3) gauge fields is defined as the solution of the gradient flow equation,

$$\partial_t B_\mu = D_\nu G_{\nu\mu}\,, \qquad B_\mu(0,x) = A_\mu(x)\,, \tag{3}$$

$$G_{\mu\nu} = \partial_\mu B_\nu - \partial_\nu B_\mu + [B_\mu, B_\nu]\,, \qquad D_\mu = \partial_\mu + [B_\mu, \cdot\,]\,, \tag{4}$$

where $A_\mu$ is the fundamental gauge field in QCD. The parameter $t > 0$ is referred to as flow time and has mass dimension $-2$. The flow equation drives the gauge field along the direction of steepest descent towards the stationary points of the Yang–Mills action. For this reason, the flow has a "smoothing" effect on the gauge field by damping its high-frequency modes.

The key feature of the gradient flow is that gauge-invariant local composite fields made out of the flow field $B_\mu$ are automatically finite once the QCD parameters (coupling and quark masses) are renormalized [11]. This allows for the definition of a compelling family of observables that can be exploited in lattice QCD calculations to probe different energy/length scales of the theory. Such observables have in fact a well-defined continuum limit without the need of any renormalization and are typically characterized by small statistical errors in Monte Carlo simulations.

One such quantity is the (Euclidean) action density at positive flow time,

$$E(t,x) = -\frac{1}{2}\sum_{\mu,\nu=0}^{3} \mathrm{tr}\left\{G_{\mu\nu}(t,x)G_{\mu\nu}(t,x)\right\}, \tag{5}$$

where $G_{\mu\nu}$ is defined in eq. (4). On the lattice, at zero flow time, this quantity would require both a multiplicative and additive renormalization, but for $t > 0$, it is automatically finite once the bare coupling and quark masses have been renormalized.

A first application of $E(t,x)$ is that of defining a reference low-energy scale $\mu_{\rm ref}$ through a specific value of the flow time, which we shall denote as $t_0$. To this end, we introduce a non-perturbative renormalized coupling [10],

$$\bar{g}^2_\infty(\mu) = \frac{16\pi^2}{3} t^2 \langle E(t,x)\rangle\Big|_{\mu=1/\sqrt{8t}}\,, \tag{6}$$

where the renormalization scale is set by the inverse of (the square root of) the flow time. In this equation the expectation value is intended in infinite space-time volume

and for physical values of the quark masses. The numerical factor in the definition (6) has been chosen so that $\bar{g}^2_\infty = g_0^2$ at lowest order in perturbation theory, where $g_0^2$ is the bare gauge coupling.

The flow time $t_0$ which defines the reference scale $\mu_{\rm ref} = 1/\sqrt{8t_0}$ is then fixed by a prescribed value of the coupling $\bar{g}^2_\infty$, specifically,

$$\bar{g}^2_\infty(\mu_{\rm ref}) = \frac{8\pi^2}{5}\,, \qquad \mu_{\rm ref} = \frac{1}{\sqrt{8t_0}}\,. \tag{7}$$

This specific value for the coupling $\bar{g}^2_\infty$ has been chosen so that the energy scale $\mu_{\rm ref}$ is a low-energy scale of about 500 MeV (cf. Section 3).[2] Clearly, the value of $t_0$ cannot be measured in any experiment. For this reason, $t_0$ is referred to as a *technical scale*. Through lattice QCD simulations, however, we can compute, e.g. $\sqrt{8t_0}m_H$, where $m_H$ is some hadron mass. Knowing $m_H$ in physical units then allows for establishing $\sqrt{8t_0}$ in physical units. Defining technical scales through specific values of non-perturbative couplings is a defining feature of our strategy to bridge the energy gap between the hadronic and perturbative regimes of QCD. In Fig. [1, Figure 2] we collected the most relevant scales defined in this way and positioned them within our strategy.

In practice it may be convenient to introduce other scales along the lines of $t_0$. One example is to consider the definition (7) but where the coupling $\bar{g}^2_\infty$ is measured for degenerate quark masses, $m_u = m_d = m_s = m$, with $m$ being close to the average of the *physical* values of the quark masses; we refer to this choice as the SU(3) flavour symmetric point. As a result one obtains a different flow time $t_0^\star$, and corresponding low-energy scale $\mu^\star_{\rm ref} = 1\sqrt{8t_0^\star}$ [12, 13]. In Section 3 the connection between $t_0$ and $t_0^\star$ is given.

We conclude by noticing that on the lattice both the gradient flow equation (3) and the field $E(t,x)$ (5) need to be discretized. In this work we exploit the $\mathrm{O}(a^2)$-improved discretization of the flow equation and of the observable proposed in [14]. We refer the interested reader to this reference for the details.

## 2.2 Schrödinger functional

The Schrödinger functional (SF) of QCD [15, 16] is another powerful tool to probe the dynamics of QCD at different scales. It is defined as the Euclidean partition function of QCD in a finite space-time volume of spatial size $L$ and temporal extent $T$, where the fields are requested to satisfy Dirichlet boundary conditions in the temporal direction at $x_0 = 0, T$. More precisely, we can write the SF as,

$$\mathcal{Z}[C, C'] = \int D[\Lambda] \int D[A,\psi,\overline{\psi}]\mathrm{e}^{-S[A,\psi,\overline{\psi}]}, \tag{8}$$

[2] We shall often refer to low-energy scales $\mu = \mathrm{O}(\Lambda_{\rm QCD})$ as hadronic scales, even though, strictly speaking, they may not be linked to any specific property of a given hadron. Simply, their values are of the same order of magnitude as typical low-lying hadronic masses.

where $S = S_g + S_f$ is the Euclidean QCD action, with,

$$S_g = -\frac{1}{2g_0^2}\int_0^T \mathrm{d}x_0 \int_0^L \mathrm{d}^3\mathbf{x}\,\mathrm{tr}\left\{F_{\mu\nu}(x)F_{\mu\nu}(x)\right\}, \tag{9}$$

$$S_f = \int_0^T \mathrm{d}x_0 \int_0^L \mathrm{d}^3\mathbf{x}\,\overline{\psi}(x)(\gamma_\mu D_\mu + m)\psi(x)\,. \tag{10}$$

Here, $g_0$ denotes the bare coupling constant, $F_{\mu\nu}$ is the field tensor associated with the gauge field $A_\mu$,

$$F_{\mu\nu} = \partial_\mu A_\nu - \partial_\nu A_\mu + [A_\mu, A_\nu], \tag{11}$$

and $D_\mu = \partial_\mu + A_\mu + i\theta_\mu/L$ is the covariant derivative acting on the quark fields. It includes a constant U(1) background field which we set to $\theta_\mu = (1-\delta_{\mu 0})\theta$, with $\theta \in [0, 2\pi)$. Specific values of $\theta$ will be prescribed later.

In the spatial directions both the quark and gluon fields are $L$-periodic. At the time boundaries, instead, the fermionic fields satisfy the Dirichlet boundary conditions [16]

$$P_+\psi(x)|_{x_0=0} = 0 = P_-\psi(x)|_{x_0=T}, \qquad \overline{\psi}(x)P_-|_{x_0=0} = 0 = \overline{\psi}(x)P_+|_{x_0=T}, \tag{12}$$

with projectors $P_\pm = \frac{1}{2}(1 \pm \gamma_0)$, where $\gamma_\mu$ are Euclidean Dirac matrices.

For the gauge field one has

$$A_k(x)|_{x_0=0} = C_k^\Lambda(\mathbf{x}), \qquad A_k(x)|_{x_0=T} = C_k'(\mathbf{x}), \qquad k = 1, 2, 3, \tag{13}$$

with boundary values $C_k(\mathbf{x})$ and $C_k'(\mathbf{x})$. The boundary condition at $x_0 = 0$ refers to the gauge transformed field,

$$C_k^\Lambda(\mathbf{x}) = \Lambda(\mathbf{x})C_k(\mathbf{x})\Lambda(\mathbf{x})^\dagger + \Lambda(\mathbf{x})\partial_k\Lambda(\mathbf{x})^\dagger\,. \tag{14}$$

The integration over the SU(3)-valued and spatially periodic gauge functions $\Lambda(\mathbf{x})$ in eq. (8) ensures gauge invariance of the Schrödinger functional.

While, in principle, the boundary values $C_k, C_k'$ can be any, in practice, it is convenient to restrict the attention to Abelian and spatially constant fields. Without loss of generality, the latter can be written as,

$$C_k(\mathbf{x}) = \frac{i}{L}\phi, \qquad C_k'(\mathbf{x}) = \frac{i}{L}\phi', \qquad k = 1, 2, 3, \tag{15}$$

where $\phi$ and $\phi'$ are traceless and diagonal $3 \times 3$-matrices. Their diagonal elements

$$\begin{aligned}
\phi_1 &= \eta - \frac{\pi}{3}, & \phi_1' &= -\eta - \pi, \\
\phi_2 &= \eta\left(\nu - \frac{1}{2}\right), & \phi_2' &= \eta\left(\nu + \frac{1}{2}\right) + \frac{\pi}{3}, \\
\phi_3 &= -\eta\left(\nu + \frac{1}{2}\right) + \frac{\pi}{3}, & \phi_3' &= -\eta\left(\nu - \frac{1}{2}\right) + \frac{2\pi}{3},
\end{aligned} \tag{16}$$

are parameterized by two real parameters, $\eta$ and $\nu$.

It is possible to show that, in the temporal gauge, the classical field equations deriving from the action $S$ with these boundary conditions are solved by,

$$\mathcal{B}_0 = 0, \qquad \mathcal{B}_k = C_k + \frac{x_0}{T}\left(C'_k - C_k\right), \qquad k = 1,2,3, \tag{17}$$

which corresponds to a constant chromo-electric field,

$$\mathcal{G}_{0k} = \partial_0 \mathcal{B}_k = \frac{C'_k - C_k}{T} = \frac{i(\phi' - \phi)}{LT}, \qquad k = 1,2,3, \tag{18}$$

all chromo-magnetic components of the field tensor being zero. The background field $\mathcal{B}$ is, up to gauge transformations, uniquely determined by the gauge boundary fields since it corresponds to the absolute minimum of the gauge action [15]. Having a unique global minimum of the action permits to straightforwardly set up a perturbative expansion considering fluctuations of the gauge field around this background-field configuration. This tremendously simplifies perturbative calculations in a finite space-time volume, if compared, for instance, to the case of periodic boundary conditions in all directions [17].

Due to the uniqueness of the background field, one may define the effective action as a function of it,

$$\Gamma[\mathcal{B}] = -\ln \mathcal{Z}[C', C], \tag{19}$$

and its perturbative expansion,

$$\Gamma[\mathcal{B}] \overset{g_0 \to 0}{\sim} \frac{1}{g_0^2}\Gamma_0[\mathcal{B}] + \Gamma_1[\mathcal{B}] + \mathrm{O}(g_0^2), \quad \Gamma_0[\mathcal{B}] = g_0^2 S_g[\mathcal{B}]\,. \tag{20}$$

Upon setting $T/L = \rho$, with $\rho$ a constant, the SF only depends on a single scale: the spatial extent $L$. We thus introduce a finite-volume coupling, $\bar{g}^2_{\mathrm{SF}}(\mu)$, which runs with the size of the box, i.e. $\mu = 1/L$, in the following way,

$$\left.\frac{\partial \Gamma[\mathcal{B}]}{\partial \eta}\right|_{\eta=\nu=0} = \frac{k}{\bar{g}^2_{\mathrm{SF}}(\mu)}\,, \qquad k = \left.\frac{\partial \Gamma_0[\mathcal{B}]}{\partial \eta}\right|_{\eta=\nu=0} = 12\pi\,, \qquad \mu = 1/L\,. \tag{21}$$

In this equation, it is understood that the quark-masses are all set to zero, the parameter $\theta = \pi/5$, and $\rho = 1$. The constant $k$ ensures the correct normalization, $\bar{g}^2_{\mathrm{SF}} = g_0^2$ to lowest order in perturbation theory. It is possible to show that $\bar{g}^2_{\mathrm{SF}}(1/L)$ is a finite, renormalized quantity, once the QCD parameters are renormalized. Hence, as anticipated, it is a suitable definition for a non-perturbative coupling. It is common to refer to $\bar{g}^2_{\mathrm{SF}}$ as *the* SF coupling.

Since the SF coupling is defined independently of perturbation theory, the same holds true for its $\beta$-function,

$$\beta(\bar{g}_{\mathrm{SF}}) = \mu \frac{\partial \bar{g}_{\mathrm{SF}}}{\partial \mu} = -L \frac{\partial \bar{g}_{\mathrm{SF}}}{\partial L} \overset{\bar{g}_{\mathrm{SF}} \to 0}{\sim} -\bar{g}^3_{\mathrm{SF}} \sum_{k=0}^{\infty} b_k \bar{g}^{2k}_{\mathrm{SF}}\,, \tag{22}$$

where the asymptotic expansion on the r.h.s. starts out with the standard universal coefficients,

$$(4\pi)^2 b_0 = \left(11 - \frac{2}{3}N_{\rm f}\right), \qquad (4\pi)^4 b_1 = \left(102 - \frac{38}{3}N_{\rm f}\right). \tag{23}$$

The scheme dependent 3-loop coefficient is given instead, for $N_{\rm f} = 3$, by [6, 18]

$$(4\pi)^3 b_2^{\rm SF}|_{N_{\rm f}=3} \;=\; -0.064(27)\,. \tag{24}$$

As discussed in more detail in the given references, the error in $b_2^{\rm SF}$ comes from an estimate of the systematic uncertainties associated with the continuum extrapolation of the relevant Feynman diagrams computed in lattice perturbation theory.

In applications, the SF coupling is determined by the expectation value,

$$\frac{k}{\bar{g}^2_{\rm SF}} = \left\langle \frac{\partial S}{\partial\eta}\bigg|_{\eta=\nu=0} \right\rangle_{{\rm SF}\mathcal{B}}, \tag{25}$$

defined in terms of the functional integral, eq. (8), with $\theta = \pi/5$, $\rho = 1$, and boundary fields (15) specified by (16) with $\eta = 0$. We denoted the corresponding expectation value by $\langle\cdot\rangle_{{\rm SF}\mathcal{B}}$ in the above equation. Following the definitions, one shows that,

$$\frac{\partial S}{\partial\eta}\bigg|_{\eta=\nu=0} = -\frac{2}{g_0^2 L}\sum_{k=1}^{3}\int_0^L {\rm d}^3\mathbf{x}\;{\rm tr}\left\{i\lambda^8\left[E_k(x)|_{x_0=0} - E_k(x)|_{x_0=T}\right]\right\}, \tag{26}$$

where $E_k = F_{0k}$ is the color-electric field and $\lambda_8 = {\rm diag}(1, -1/2, -1/2)$.

The SF can be naturally formulated on the lattice. We refer the interested reader to the original references for the details [15, 16]. Here we simply remark that due to the presence of the Dirichlet boundary conditions at $x_0 = 0, T$ the corresponding Symanzik effective theory for the SF allows for the presence of ${\rm O}(a)$-counterterms localized at the boundary [15, 19], i.e. mass-dimension 4 fields. These translate into ${\rm O}(a)$ discretization errors in continuum limit extrapolations of observables defined with the SF. Following Symanzik's improvement programme, given our choice of boundary conditions, these ${\rm O}(a)$ effects can be eliminated by properly adjusting one gluonic and one fermionic ${\rm O}(a)$-counterterms added to the lattice action. Their coefficients are conventionally denoted as $c_{\rm t}(g_0)$ and $\tilde{c}_{\rm t}(g_0)$, respectively, and are functions of the bare gauge coupling [15, 19].

## 2.3 Gradient-flow couplings

The SF allows for the definition of other useful finite-volume couplings. One particularly compelling family is obtained by considering the action density at positive flow

time, eq. (5). More precisely, we shall consider its magnetic component,

$$E_{\rm m}(t,x) = -\frac{1}{2}\sum_{k,l=1}^{3} {\rm tr}\left\{G_{kl}(t,x)G_{kl}(t,x)\right\}. \tag{27}$$

Note that due to the different treatment of time and spatial boundary conditions in the SF, the expectation values of the (color-)magnetic and electric components of the action density are different. In order for a flow-based coupling to depend only on the spatial size $L$ as a scale, we fix the flow time $t$ at which $E_{\rm m}(t,x)$ is measured in a fixed ratio $c = \sqrt{8t}/L$ with $L$. A non-perturbative, finite-volume, gradient-flow coupling can then be defined as [20]

$$\bar{g}^2_{\rm GF}(\mu) = \mathcal{N}^{-1} t^2 \langle E_{\rm m}(t,x)\rangle_{\rm SF}\Big|^{T=L}_{x_0=T/2,\,\sqrt{8t}/L=0.3}\,, \qquad \mu = 1/L\,. \tag{28}$$

In this equation, it is understood that the quark masses are all set to zero. The expectation value $\langle\cdot\rangle_{\rm SF}$ then refers to the functional integral, eq. (8), with $\theta = 1/2$, and boundary fields (15) specified by $\phi = \phi' = 0$. The constant $\mathcal{N}$ is a normalization factor that guarantees $\bar{g}^2_{\rm GF} = g_0^2$ at the lowest order in perturbation theory. The choice of measuring the flow action density at $x_0 = T/2$ is for maximizing its distance from the time-boundaries at $x_0 = 0, T$. On the lattice, this grants a significant reduction of the O($a$) discretization errors induced by the SF compared to placing the action density closer to the boundaries. For the same reason, it is advisable to restrict the attention to the magnetic component of the action density (27), as opposed to (5) (cf. refs. [20, 21] for a discussion). Values of $c$ in the range $0.2 < c < 0.5$ are typically considered in applications, with smaller values favoring smaller statistical errors in Monte Carlo simulations, but larger discretization errors (viceversa for large values). In our case, we choose $c = 0.3$, which was shown in many applications to be a good compromise (cf. e.g. [20, 21]). We note that, for the definition (28) to be actually complete, the expectation value is missing a projection to gauge field configurations of trivial topology. However, this is a rather technical detail, which avoids the issue of topology freezing in lattice QCD simulations [22, 23]. For the ease of presentation, we prefer to skip over this detail here. The interested reader can find a complete discussion in ref. [21], where the details of the lattice definition of the GF coupling (28) are also given.

For the study of the decoupling of heavy quarks and the matching of $N_{\rm f} = 3$ QCD with the pure gauge theory, it is convenient to define a different, although closely related gradient-flow based coupling. We denote this second coupling as GFT-coupling and its definition is given by,

$$\bar{g}^2_{\rm GFT}(\mu, M) = \mathcal{N}_T^{-1} t^2 \langle E_{\rm m}(t,x)\rangle_{\rm SF}\Big|^{T=2L}_{x_0=T/2,\,\sqrt{8t}/L=0.36, ML=z}\,, \qquad \mu = 1/L\,. \tag{29}$$

There are three differences compared to the definition of the GF-coupling, eq. (28). First, the GFT-coupling is a massive, rather than massless coupling. In eq. (29), the

quark masses are all equal and tuned so that their corresponding RGI mass $M = z\mu = z/L$, where $z$ is a chosen constant. Second, the time extent $T$ is twice as large for a given $L$ than for the GF-coupling, i.e. $\rho = T/L = 2$. As discussed in more detail in Section 10, this significantly reduces the $\mathrm{O}(1/M)$ corrections to the large-mass limit of the GFT-coupling compared to the standard $T = L$ set-up. Finally, we prefer a slightly larger value of $c = 0.36$ for the GFT-coupling compared to the GF-coupling. While a larger value of $c$ leads to larger $\mathrm{O}(1/M)$ corrections from the boundary (cf. again the discussion in Sect. 10), it reduces the dominant $\mathrm{O}(1/M^2)$ bulk-corrections in the large-mass extrapolations. This is because the largest energy scale that characterizes the coupling (29) is $\mu = 1/\sqrt{8t} = 1/(cL)$, and this is smaller for larger values of $c$ (cf. ref. [24] for more details).

# 3 Scale setting

Our result for the strong coupling depends on the value of a technical scale $\mu_{\mathrm{had}}$ that has to be determined in physical units. This scale is implicitly defined by specifying a precise value for the massless coupling in 3 flavor QCD: $\bar{g}^2_{\mathrm{GF}}(\mu_{\mathrm{had}}) = 11.31$ (see [13]). Ultimately, $\mu_{\mathrm{had}}$ has to be determined from a well measured experimental quantity (like for instance the mass of the $\Omega$ baryon). In practice, it is convenient to introduce an intermediate technical length scale, $\sqrt{t_0^\star}$, derived from the gradient flow [10]. This scale differs from the more common scale $\sqrt{t_0}$ by the choice of quark masses: while $\sqrt{t_0}$ is defined for physical values of the quark masses, $\sqrt{t_0^\star}$ is defined for three degenerate quark masses $m_u = m_d = m_s$ such that $12 t_0 m_\pi^2 = 1.11$ (see [13] for further details). Our estimate for $\sqrt{t_0/t_0^\star}$, is based on [25] but with an enlarged error which also covers the central value from [26]. Yet, the uncertainty on

$$\sqrt{\frac{t_0}{t_0^\star}} = 1.0003(3)_{\mathrm{stat}}(30)_{\mathrm{sys}}(30)_{\mathrm{tot}} \tag{30}$$

is negligible for our purposes. This ratio allows us to determine $\sqrt{t_0^\star}$ from the results for $\sqrt{t_0}$ from different lattice collaborations. The estimates for the reference scale $\sqrt{t_0^\star}$ obtained in this way are displayed in [1, Extended Data Figure 3]. We only show the computations that pass the averaging criteria set by FLAG [27].[3] These computations were performed using a range of simulations that allows for controlling the main sources of systematic uncertainties in lattice computations (i.e. continuum limit, infinite volume extrapolation, chiral extrapolation, ...). They also use different experimental quantities as input (like the mass of the $\Omega$ baryon, or leptonic decay rates of Pions and Kaons; for details see [27]). Despite satisfying the FLAG criteria, the different data in [1, Extended Data Figure 3] show some tension. At present, there is no clear reason for this tension. Therefore we opt for a robust average. Our mean value for $\sqrt{t_0^\star}$ is given by the weighted average of all results, but we increase the error to include the central values of all computations which exert a pull of two or more on

[3] Note that CLS21 [26] supersedes CLS16 [13] considered in FLAG [27].

the weighted average.[4] As a result, we use

$$\sqrt{t_0^\star} = 0.1433(7)_{\rm stat}(4)_{\rm sys}(17)_{\rm robust}(19)_{\rm tot}\,{\rm fm}\,. \tag{31}$$

Exploiting this result and the dimensionless combination

$$\sqrt{t_0^\star}\,\mu_{\rm had} = 0.1457(11)_{\rm stat}(01)_{\rm sys}(11)_{\rm tot}\,, \tag{32}$$

that was accurately determined in [13], we can quote a value for the scale $\mu_{\rm had}$ in physical units

$$\mu_{\rm had} = 200.6(1.8)_{\rm stat}(0.6)_{\rm sys}(2.3)_{\rm robust}(3.0)_{\rm tot}\,{\rm MeV}\,. \tag{33}$$

Despite the modest precision on this scale (1.5%), its uncertainty is subdominant in the final uncertainty of the strong coupling. However, it does contribute by far the largest systematic error to our final result. We expect that it will be reduced significantly in the next few years, since the lattice community as a whole needs a precise scale determination and is investing significant effort into it [28]. For this reason we are listing this uncertainty separately by labeling it as robust (see section 1).

# 4 The running coupling at low energies

Once the scale $\mu_{\rm had}$ is determined in physical units, one needs to compute the running of the coupling from $\bar{g}^2_{\rm GF}(\mu_{\rm had}) = 11.31$, up to high energy scales, where perturbation theory accurately describes QCD. The determination of the $\beta$ function follows the strategy explained in methods and used in [21]. First, we determine the lattice step scaling function in the GF scheme

$$\Sigma(u, a/L) = \bar{g}^2_{\rm GF}(\mu/2)\Big|_{\bar{g}^2_{\rm GF}(\mu)=u}\,. \tag{34}$$

Our data set includes estimates of $\Sigma(u, a/L)$ from lattices with sizes $L/a = 8, 12, 16$, at nine different values of $u$, as considered in [21]. Moreover we have new data [30–33] much closer to the continuum, with $L/a = 20, 24, 32$, at two specific values of $u = 3.949, 5.8673$. This data set allows us to determine precisely the step scaling function in the continuum

$$\sigma(u) = \lim_{a/L\to 0} \Sigma(u, a/L)\,. \tag{35}$$

We use a linear functional form in $a^2$ for the continuum extrapolations. As explained in detail in [21, 26], our data shows significant discretization effects. These are well described by a simple $a^2$ term, but different subleading terms, e.g., $a^4$, can affect the result of the extrapolations. Therefore we opt to give less importance to the data at

[4] The pull is defined as the contribution to the $\chi^2$ of the fit to a constant. Computations with a small pull either have a central value that agrees well with the mean or they have a large uncertainty and are therefore not so relevant in determining the mean.

coarse lattice spacing. We do so by considering a weight $[\Delta_i]^{-2}$ which includes the statistical error $\Delta_i^{\rm stat}$ and a term reducing the weight of data further away from the continuum. It has the expected dominating $a^4$ contamination built in,

$$\Delta_i^2 = [\Delta_i^{\rm stat}]^2 + [\Delta_i^{\rm sys}]^2\,, \quad \Delta_i^{\rm sys} = 0.05\,\Sigma_i \left(8\frac{a}{L}\right)^4 \frac{u}{u_{\rm max}}\,. \tag{36}$$

The term $[\Delta_i^{\rm sys}]^2$ is negligible compared to our statistical uncertainties at all values of $u$ for lattices with $L/a \geq 12$, but it becomes dominant at large values of $u$ for $L/a = 8$. As a result, in addition to reducing the relevance of data far away from the continuum, the uncertainties of our results in the continuum are also increased. Eq. (36) is unchanged compared to our previous study [21], but we now have data closer to the continuum. A comparison of our previous extrapolations and those with the new data set show a considerable reduction of the error (cf. [1, Extended Data Figure 1]). Central values are in total agreement. This validates our approach to perform the continuum extrapolations.

We parameterize the $\beta$-function as

$$\beta(x) = -\frac{x^3}{P_N(x^2)}\,, \tag{37}$$

where $P_N(x^2)$ is a polynomial of degree $N$ in $x^2$, and extract its coefficients by fitting the continuum step scaling function data, $\sigma$, to

$$\ln 2 = \int_{\sqrt{u}}^{\sqrt{\sigma(u)}} \frac{{\rm d}x}{\beta(x)}\,. \tag{38}$$

Alternatively one can directly fit the lattice step scaling function $\Sigma(u, a/L)$ by including the cutoff effects in the fitting ansatze as

$$\ln 2 + \left(\frac{a}{L}\right)^2 Q_{n_c}(u) = \int_{\sqrt{u}}^{\sqrt{\Sigma(u,a/L)}} \frac{{\rm d}x}{\beta(x)}\,, \tag{39}$$

where $Q_{n_c}(u)$ is a polynomial in $u$ of degree $n_c$ without constant term. We obtain good fits with $N > 1$ and $n_c > 1$.[5] We choose the values $N = n_c = 2$ to quote our final result for the $\beta$ function, whose continuum form and validity range reads

$$\beta(x) = -\frac{x^3}{p_0 + p_1 x^2 + p_2 x^4}\,, \qquad \left(x^2 \in [2.2, 11.5]\right)\,. \tag{40}$$

The values of the parameters $p_i$ and their covariance can be checked in the accompanying replication package [35, file `running_le.jl`].

[5] See [34] for how one determines the quality of fits with general weights such as the ones described above.

This result allows us to compute some key ratios of scales. Specifically, from the definitions

$$\bar{g}^2_{\rm GF}(\mu_{\rm had}) = 11.31\,, \quad \bar{g}^2_{\rm GF}(\mu_{\rm dec}) = 3.949\,, \tag{41}$$

and the value

$$\bar{g}^2_{\rm GF}(\mu_0/2) = 2.6723(64)\,, \tag{42}$$

we obtain

$$\frac{\mu_{\rm dec}}{\mu_{\rm had}} = 4.000(26)_{\rm stat}(25)_{\rm sys}(36)_{\rm tot}\,, \tag{43}$$

$$\frac{\mu_0}{\mu_{\rm dec}} = 5.464(61)_{\rm stat}(17)_{\rm sys}(62)_{\rm tot}\,, \tag{44}$$

$$\frac{\mu_0}{\mu_{\rm had}} = 21.86(32)_{\rm stat}(17)_{\rm sys}(34)_{\rm tot}\,. \tag{45}$$

Given these ratios and the results of section 3, we can determine all these scales in physical units,

$$\mu_{\rm had} = 200.6(1.8)_{\rm stat}(0.6)_{\rm sys}(2.3)_{\rm robust}(3.0)_{\rm tot}\,{\rm MeV}\,, \tag{46a}$$

$$\mu_{\rm dec} = 803(9)_{\rm stat}(6)_{\rm sys}(9)_{\rm robust}(14)_{\rm tot}\,{\rm MeV}\,, \tag{46b}$$

$$\mu_0 = 4385(71)_{\rm stat}(36)_{\rm sys}(51)_{\rm robust}(94)_{\rm tot}\,{\rm MeV}\,. \tag{46c}$$

# 5 The strong coupling from $N_{\rm f} = 3$ QCD

Given the knowledge of a hadronic scale $\mu_{\rm had}$ determined from the QCD spectrum (see section 3), and a precise running of the strong coupling from $\mu_{\rm had} \approx 200\,{\rm MeV}$ to $\mu_0 \approx 4\,{\rm GeV}$ at hand, it is time to match with perturbation theory and extract the value of the strong coupling at high energy.

One may be tempted to match with perturbation theory directly at $\mu_0$; in fact perturbation theory is routinely used at these energy scales. This, however, would produce a result with very small statistical uncertainties (below 0.2% in $\alpha_s(m_Z)$), but with a difficult-to-estimate theoretical uncertainty. The defining feature of the strategy pursued by the ALPHA collaboration consists instead in pushing the computation of the non-perturbative running of the coupling to much higher energies, up to the electroweak scale, where the theoretical uncertainties associated with the use of perturbation theory are negligible. The trade off is the larger statistical uncertainty that the determination of this non-perturbative running carries.

In [6, 36] we determined the ratio

$$\frac{\Lambda^{(3)}_{\overline{\rm MS}}}{\mu_0} = 0.0792(15)_{\rm stat}(11)_{\rm sys}(19)_{\rm tot}\,. \tag{47}$$

We recall that this result was obtained by matching with perturbation theory at a scale $\mu_{\rm PT} \approx 70$ GeV. The convergence of the perturbative approach at high energy scales was checked using three different renormalization schemes, and perturbative uncertainties were estimated in many different ways, including scale variation [6].

Compared with [13], our result also includes a more accurate modeling of the $\mathcal{O}(a)$ boundary effects [6]. Together with the result for $\mu_0$ (Eq. (46)), we get

$$\Lambda_{\overline{\rm MS}}^{(3)} = 347(9)_{\rm stat}(6)_{\rm sys}(4)_{\rm robust}(11)_{\rm tot}\,{\rm MeV}\,. \tag{48}$$

The central value has moved by less than half a standard deviation from our previous result [13]. This shift is dominantly due to the change in the scale $\sqrt{t_0^\star}$. Despite the fact that the new dataset reduces the uncertainty in $\mu_0/\mu_{\rm had}$ by 20%, and we also have a slight reduction of the error in $\Lambda_{\overline{\rm MS}}^{(3)}/\mu_0$ (Eq. (47)), our determination of $\Lambda_{\overline{\rm MS}}^{(3)}$ shows only a marginal improvement in precision compared with our previous computation. Nevertheless, this update is important for two reasons. First, our analysis techniques, and in particular our method for calculating the continuum limit of the step scaling function at low energy, have been validated. Second, it highlights the crucial point that our uncertainties are primarily governed by the statistical uncertainties on the running in the high energy regime. In conclusion, using a non perturbative approach for computing the running of the strong coupling up to the electroweak scale, even if it removes the theoretical uncertainty, results in a substantial statistical uncertainty. This is reducible through extensive computational efforts, but would profit from an alternative strategy.

# 6 Decoupling of heavy quarks

As we commented, the uncertainty in $\Lambda_{\overline{\rm MS}}^{(3)}$ is dominated by the high energy running. The challenge is therefore to improve the statistical uncertainty in computing the running coupling at high energies. Here we use the alternative strategy based on the decoupling of heavy quarks introduced in [37] and applied in [24]. This approach achieves a high precision by shifting the step scaling computations from QCD to the pure gauge theory, where much better precision can be achieved (see [8, 9]).

Let us summarize the decoupling strategy as described in [24]. Using a massive scheme for the coupling, $\bar{g}^2_{\rm GF}(\mu, M)$, where all $N_{\rm f}$ quarks are taken degenerate with renormalization group invariant (RGI) mass $M$, this coupling tends to its pure gauge theory counterpart in the limit of large quark masses, $M \gg \Lambda, \mu$.[6] In formulas,

$$\bar{g}^2_{\rm GF}(\mu, M) = [\bar{g}^{(0)}_{\rm GF}(\mu)]^2 + {\rm O}(\Lambda^2/M^2, \mu^2/M^2)\,, \tag{49}$$

where the power corrections of order $\Lambda^2/M^2$ and $\mu^2/M^2$ were explicitly written. The shorthand notation used in this equation hides the fact that for a given $\mu$ (which we shall set equal to $\mu_{\rm dec}$ in our numerical application), the pure gauge coupling $\bar{g}^{(0)}_{\rm GF}(\mu)$ depends on the mass $M$ through the matching of the $\Lambda$-parameters of the two theories, which reads,

$$\Lambda^{(0)}_{\overline{\rm MS}} = P(M/\Lambda_{\overline{\rm MS}})\,\Lambda_{\overline{\rm MS}}\,. \tag{50}$$

As discussed in [38], the function $P$ is perturbatively known to very high accuracy. By this we mean that the relation is known to a high order in perturbation theory and the higher order corrections decrease very rapidly for the values of the masses of interest.

[6] We refer the reader to Section 9 for a proper definition of $M$.

In perturbation theory, the function $P$ can be computed as,

$$P(M/\Lambda_{\overline{\rm MS}}) = \frac{\varphi^{(0)}_{\overline{\rm MS}}(g_\star\sqrt{C(g_\star)})}{\varphi_{\overline{\rm MS}}(g_\star)}\,. \tag{51}$$

In this equation $\varphi^{(0)}_{\overline{\rm MS}}(g)$ and $\varphi_{\overline{\rm MS}}(g)$ refer to the function eq. ([1].8) for 0 and 3 flavours, respectively, in the $\overline{\rm MS}$ scheme. The coupling $g_\star = \bar{g}_{\overline{\rm MS}}(m_\star)$, where $m_\star$ is defined by the implicit equation $m_\star = \overline{m}_{\overline{\rm MS}}(m_\star)$ with $\overline{m}_{\overline{\rm MS}}(\mu)$ the running quark mass in the $\overline{\rm MS}$ scheme at the scale $\mu$ (see section 9). Note that the coupling $g_\star$ is a function of $M/\Lambda_{\overline{\rm MS}}$ alone, i.e. $g_\star \equiv g_\star(M/\Lambda_{\overline{\rm MS}})$ [24, 38]. The function $C(g)$ relates the couplings in the 3 flavour and pure gauge theory in the $\overline{\rm MS}$ scheme as,

$$[\bar{g}^{(0)}_{\overline{\rm MS}}(m_\star)]^2 = C(\bar{g}_\star)\bar{g}_\star^2\,, \tag{52}$$

and it is known to 4-loop order in perturbation theory [40–42].

Dividing eq. (50) on both sides by $\mu_{\rm dec}$ yields eq. ([1].30) which we rewrite here in a more precise form by including the power corrections from eq. (49),

$$\rho P\left(z/\rho\right) = \frac{\Lambda^{(0)}_{\overline{\rm MS}}}{\Lambda^{(0)}_{\rm GF}} \times \varphi^{(0)}_{\rm GF}\left(\bar{g}_{\rm GF}(\mu_{\rm dec}, M)\right) + {\rm O}(\Lambda^2/M^2, \mu^2/M^2)\,, \qquad \rho \equiv \Lambda_{\overline{\rm MS}}/\mu_{\rm dec}\,. \tag{53}$$

The scheme change $\Lambda^{(0)}_{\overline{\rm MS}}/\Lambda^{(0)}_{\rm GF} = 0.4981(17)$ [43] involves no approximation by virtue of eq. ([1].12); we profit from the full accuracy of $P$ in the $\overline{\rm MS}$ scheme by working in terms of $\Lambda$-parameters.[7]

In [24] we computed the massive coupling $\bar{g}^2_{\rm GF}(\mu_{\rm dec}, M)$ for values of the quark masses[8]

$$M/\mu_{\rm dec} \approx 4, 6, 8, 10, 12\,. \tag{54}$$

The determination of $\bar{g}^2_{\rm GF}(\mu, M)$ requires a continuum extrapolation of the corresponding lattice estimates at non-zero lattice spacing. These extrapolations are difficult. On the one hand, solid continuum extrapolations require $aM \ll 1$. On the other hand, large values of $M$ are needed in order to have small ${\rm O}(1/M^2)$ corrections in eq. (53). This multi scale problem can only be solved by employing large lattices in a finite volume scheme. These schemes avoid in fact the introduction of any additional scale since $\mu = 1/L$ (see [24, 37] for the details).

An additional problem in performing these continuum extrapolation is the presence of linear $aM$ cutoff effects. The most critical ones to deal with originate from a term $\sim aM\,{\rm tr}\,F^2$ in the Symanzik effective theory, which describes the expansion of lattice observables in powers of the lattice spacing $a$ [19, 44–47], accompanied by (non-integer) powers of $\alpha(1/a)$ [48–52]. The $\sim aM\,{\rm tr}\,F^2$ term can be removed by a proper change of the bare coupling $g_0$ as one varies the quark mass [19]. More precisely, when computing

[7] We note that section 11 contains a perturbative analysis of the decoupling strategy based on eq. (53). This may provide the reader with a different and useful perspective on the general ideas presented in this and the following sections.

[8] A precise definition of the massive coupling that we considered is given in eqs. (55) and (29).

the mass-dependence of an observable such as $\bar{g}^2_{\rm GF}(\mu, M)$, the quantity to keep fixed in order to have a fixed lattice spacing $a$ is $\tilde{g}^2_0 = g^2_0(1 + b_{\rm g}(\tilde{g}^2_0)am_q)$ rather than $g_0$. This guarantees that the $\mathrm{O}(aM)$ effects due to the $\sim aM\,\mathrm{tr}\,F^2$ term are absent in continuum extrapolations.[9]

Until recently, the improvement parameter $b_{\rm g}(\tilde{g}^2_0)$ was only known to leading order in perturbation theory [53]. This approximation was used in our previous work [24]. Our error included an estimate of the effect that the difference between the (at the time unknown) non-perturbative value of $b_{\rm g}$ and its 1-loop approximation could have on the values of the coupling at finite lattice spacing. This estimate dominated the uncertainty of the continuum extrapolation of the massive couplings $\bar{g}^2_{\rm GF}(\mu, M)$. In particular, this source of uncertainty was systematic and could only be estimated through perturbative arguments. Below we shall rely on our recent non-perturbative determination of $b_{\rm g}$ [54] in order to obtain precise continuum extrapolations of the massive coupling $\bar{g}^2_{\rm GF}(\mu_{\rm dec}, M)$ free of this systematic uncertainty.

## 6.1 Continuum limit of the massive couplings

We start with a precise definition of the massive coupling. The Gradient Flow coupling is defined with Schrödinger Functional boundary conditions as [20]

$$\bar{g}^2_{\rm GFT}(\mu, M) = \frac{1}{\hat{\mathcal{N}}(t, a/L)} t^2 \langle E(t,x)\rangle\Big|_{T=2L,\, x_0=T/2,\, \sqrt{8t}=0.36\,L}\,, \tag{55}$$

where $E(t, x)$ is the energy density of the flow fields and $\hat{\mathcal{N}}$ a normalization constant. We focus on the preferred value $c = 0.36$ for $c = \sqrt{8t}/L$, but we thoroughly checked that different choices, $c \in \{0.30, 0.33, 0.36, 0.39, 0.42\}$, lead to completely equivalent results for $\Lambda^{(3)}_{\overline{\rm MS}}$. Note the choice $T = 2L$, where $T$ is the time extent of our space-time lattices. It defines the $\mathrm{x} = \mathrm{GFT}$ scheme, which is employed only for the massive coupling, while everywhere else, e.g. in the definition of $\mu_{\rm dec}$, the $\mathrm{x} = \mathrm{GF}$ scheme with $c = 0.3$ and $T = L$ is used. A non-perturbative matching of the two schemes will be discussed below. The primary reason for having $T = 2L$ for the massive coupling is that with this choice boundary effects $\propto 1/M$ are suppressed to a level well below our statistical uncertainties (see section 10 for details).

### Couplings on lines of constant physics

The massive couplings are needed at fixed $L = 1/\mu_{\rm dec}$ and for several values of the RGI mass $M$. Furthermore, the simulations employ a certain resolution $a/L$. The dimensionless quantities defining $\mu_{\rm dec}$ and $M$ are $\bar{g}^2_{\rm GF}(\mu_{\rm dec}) = 3.949$ and $z = M/\mu_{\rm dec} = ML$, where it is understood that $\bar{g}^2_{\rm GF}(\mu_{\rm dec})$ is defined for $M = 0$, i.e. for a bare subtracted mass $m_{\rm q} = 0$. As determined in [24], the bare couplings $\tilde{g}^2_0$ listed in [1, Extended Data Table 2] ensure $\bar{g}^2_{\rm GF}(\mu_{\rm dec}) = 3.949$. The necessary values of $m_{\rm q}$ then follow from $aM = z \times a/L$ and the ALPHA-collaboration's work on the non-perturbative quark mass renormalization, which we summarize in section 9. Given $am_{\rm q}$, one needs to

[9] The Symanzik effective theory also predicts a term $\sim aM^2\overline{\psi}\psi$. This, however, can be easily removed through a proper definition of the RGI quark mass $M$ (see section 9 for more details).

perform the massive simulations with bare couplings $g_0^2$ such that the improved bare coupling $\tilde{g}_0^2 = (1 + b_{\rm g}(\tilde{g}_0^2) am_{\rm q})\, g_0^2$ is kept fixed. As discussed earlier, the improvement coefficient $b_{\rm g}$ is needed to cancel linear $a$-effects when connecting the theories at different masses, but fixed lattice spacing. For our range of $\tilde{g}_0^2$, the non-perturbative $b_{\rm g}$ is given by [54]

$$b_{\rm g}(\tilde{g}_0^2) = b_{\rm g}^{(1)} \tilde{g}_0^2 - 0.0151\tilde{g}_0^4 + 0.0424\tilde{g}_0^6\,, \quad b_{\rm g}^{(1)} = 0.036\,. \tag{56}$$

However, our simulations were performed when only the 1-loop approximation $b_{\rm g} = 0.036\tilde{g}_0^2 + {\rm O}(\tilde{g}_0^4)$ was available. We deal with this mismatch by correcting the data for $\bar{g}^2_{\rm GFT}(\mu_{\rm dec}, M)$ in Table 2 of [24] for the change in the bare coupling due to the change in $b_{\rm g}$ via

$$\bar{g}^2_{\rm GFT,corrected}(\mu_{\rm dec}, M) = \bar{g}^2_{\rm GFT}(\mu_{\rm dec}, M) - \frac{{\rm d}\bar{g}^2_{\rm GFT}(\mu_{\rm dec}, M)}{{\rm d}\tilde{g}_0^2} \Delta b_{\rm g}\, \tilde{g}_0^2\, am_q\,. \tag{57}$$

The straightforward $\Delta b_{\rm g} = b_{\rm g}(\tilde{g}_0^2) - b_{\rm g}^{(1)}\tilde{g}_0^2$ will be modified below.

As discussed in detail in [24], the derivative

$$\frac{{\rm d}\bar{g}^2_{\rm GFT}(\mu_{\rm dec}, M)}{{\rm d}\tilde{g}_0^2} = \frac{\bar{g}_{\rm GFT}\beta^{(0)}_{\rm GFT}(\bar{g}_{\rm GFT})(1-\eta^{\rm M}(g_\star))}{\tilde{g}_0 \beta_0^{(3)}(\tilde{g}_0)}[1 + R_{\rm z} + R_{\rm a}]\,, \tag{58}$$

is known with uncertainties that affect the corrected couplings only well below their statistical uncertainties. Here we shall only quote the final expression for this derivative, but encourage the interested reader to consult the original references to understand its derivation (see appendix D of [24] and references therein). We thus take

$$\beta^{(0)}_{\rm GFT}(x) \approx -k_0\, x^3\,, \tag{59}$$

$$1 - \eta^{\rm M}(g_\star) \approx \frac{9}{11}\,, \tag{60}$$

$$\beta_0^{(3)}(x) \approx -0.054\, x^3\,, \tag{61}$$

$$R_z \approx k_0 \frac{b}{z^2} \bar{g}^2_{\rm GFT}\,, \tag{62}$$

$$R_a \approx \frac{p_1 + p_2 z^2}{\bar{g}_{\rm GFT}\beta^{(0)}_{\rm GFT}(\bar{g}_{\rm GFT})(1-\eta^{\rm M}(g_\star))} \left(\frac{a}{L}\right)^2\,, \tag{63}$$

where for the case $c = 0.36$ we have: $p_1 = -21.4, p_2 = 1.08, b = 10.54$, and $k_0 = 0.076$.

Beside the accuracy of the derivative (58) itself, we also need to worry about the size of the quadratic (and higher order) terms in $\Delta b_{\rm g}$ which are neglected in the linear approximation (57). We therefore want small $\Delta b_{\rm g}$ values. This can be achieved by adding to $\Delta b_{\rm g}$ a term which only modifies the ${\rm O}((aM)^2)$ terms in $\bar{g}^2_{\rm GFT,corrected}$, i.e. it is linear in $aM$,

$$\Delta b_{\rm g} = b_{\rm g}(\tilde{g}_0^2) - b_{\rm g}^{(1)}\tilde{g}_0^2 + c_{\rm M} \times (aM)\,. \tag{64}$$

As long as the linear approximation (57) remains accurate, the coefficient $c_{\mathrm{M}}$ can be chosen at will. As anticipated, its effect is just an additional cutoff effect of $\mathrm{O}((aM)^2)$ in the massive couplings, which does not affect the continuum values. For $c_{\mathrm{M}} = -0.2$ the shift in $\bar{g}_{\mathrm{GFT}}$ is very small for the data point with $L/a = 12$ and $z = 6$. This choice results in very small shifts also for all other lattices that are relevant for the continuum extrapolation. The maximum value of $|\Delta b_{\mathrm{g}}|$ is 0.03 and the maximum shift $|\bar{g}^2_{\mathrm{GF,corrected}} - \bar{g}^2_{\mathrm{GF}}|$ is 0.1 . As a confirmation that the choice of $c_{\mathrm{M}}$ is not crucial, we repeated the entire analysis with $c_{\mathrm{M}} = -0.1$ and $c_{\mathrm{M}} = -0.3$, see [1, Extended Data Table 2].

### Continuum extrapolation

For the continuum extrapolation we consider the fit ansatz

$$\bar{g}^2_{\mathrm{GFT}}(\mu_{\mathrm{dec}}, M_i) = \bar{g}^2(z_i) + p_1[\alpha_s(a^{-1})]^{\hat{\Gamma}_{\mathrm{eff}}} a^2\mu^2_{\mathrm{dec}} + p_2[\alpha_s(a^{-1})]^{\hat{\Gamma}'_{\mathrm{eff}}}(aM_i)^2\,, \qquad (65)$$

where $\bar{g}^2(z_i), p_1, p_2$ are fit parameters and $\alpha_s \equiv \alpha^{(N_{\mathrm{f}}=3)}_{\overline{\mathrm{MS}}}$. The fit parameters $\bar{g}^2(z_i)$ are the desired continuum couplings. The fit function is a result of an analysis of the Symanzik effective theory in the limit of large $M$ [24]. Dropping relative corrections of order $\mathrm{O}(1/M^2)$ in the effective theory reveals that there are no cross-terms of the from $a^2M\mu_{\mathrm{dec}}$ and that there are global coefficients $p_1, p_2$ instead of individual terms $\tilde{p}_i a^2$ for each value of $z_i = M_i/\mu_{\mathrm{dec}}$. Due to the expansion in $1/M$, the functional form eq. (65) is only expected to describe data at large values of $M$. For our case it is a very good approximation for $z = M/\mu_{\mathrm{dec}} \geq 4$. Indeed, the slopes in the extrapolations in $(aM)^2$ in [1, Extended Data Figure 4] (left) vary little with $z$.

Table [1, Extended Data Table 2] collects the data that is included in the global fit, as well as the results of the continuum extrapolations. We also give the continuum values for several choices of the shift parameter $c_{\mathrm{M}}$. As we can see from the results in the table, varying $c_{\mathrm{M}}$ in the range $-0.1, -0.3$ around our preferred value $-0.2$ results in variations of the continuum values of at most 1/3 of the statistical uncertainties.

The effect of the non-perturbative knowledge of $b_{\mathrm{g}}$ is crucial for the accuracy of our continuum limits. Compared with our previous results of [24], our continuum data is now between three to four times more precise. It is also worth noting that after the correction the data is much more linear in $a^2$. Judging by the quality of the fit alone, we could in fact extend the fitting range to include data up to $(aM)^2 \approx 0.3$ (and thus reduce the statistical uncertainties in the continuum even further). We however decided to quote as final result the fit that only includes data with $(aM)^2 < 0.16$. This approach is conservative, since it leads to larger uncertainties and entirely compatible central values. In addition, this choice keeps the effect of the logarithmic corrections in the continuum extrapolation under control. Through the Symanzik effective theory analysis in the large quark-mass limit presented in ref. [24] we established sensible ranges for the exponents $\hat{\Gamma}_{\mathrm{eff}}, \hat{\Gamma}'_{\mathrm{eff}}$ in eq. (65) (cf. section 4.4 of that reference). Varying them in such ranges: $\Gamma_{\mathrm{eff}} \in [-1, 1]$ and $\Gamma'_{\mathrm{eff}} \in [-1/9, 1]$, the effect that we observe is typically of about the same size as the statistical errors on the continuum values. As

we shall discuss later, this effect is subdominant in $\Lambda_{\overline{\rm MS}}^{(3)}$ and at most of the order of 10% of the statistical uncertainties on $\Lambda_{\overline{\rm MS}}^{(3)}$ (cf. [1, Extended Data Figure 5]).

In conclusion, we are able to obtain precise values for the massive coupling in the continuum, where the choice of different ansätze for the continuum extrapolations results in values for $\Lambda_{\overline{\rm MS}}^{(3)}$ that vary much less than the statistical fluctuations.

## 6.2 The determination of $\Lambda_{\overline{\rm MS}}^{(3)}$

In our strategy, eq. (53), $\Lambda_{\overline{\rm MS}}^{(3)}$ is determined via the pure gauge running function $\varphi_{\rm GF}^{(0)}(g)$ in the GF scheme. On the other hand, the systematic errors due to boundary effects in the Schrödinger Functional are much easier to control for the GFT coupling, eq. (55). The idea is therefore to first apply the decoupling relation, eq. (49), to the GFT coupling, and in a second step switch to the GF scheme through the (pure gauge theory) function

$$\bar{g}_{\rm GF}^{(0)}(\mu) = \chi_{0.36}(\bar{g}_{\rm GFT}^{(0)}(\mu)) \,. \tag{66}$$

Note that the scheme switch also involves the change in the value of $c$ from $c = 0.36$ (GFT) to $c = 0.30$ (GF). (Other values for $c$ in the GFT scheme have been considered for consistency checks.)

The precise form in which we apply the relation eq. (53) is thus the following,

$$\rho^{\rm eff} P\left(z/\rho^{\rm eff}\right) = F\left(\chi_{0.36}(\bar{g}_{\rm GFT}(\mu_{\rm dec}, M)\right) \,, \quad \rho^{\rm eff} \equiv \Lambda_{\overline{\rm MS}}^{\rm eff}/\mu_{\rm dec} \,, \tag{67}$$

where we absorbed all power corrections of $\mathrm{O}(1/M^2)$ into an effective $\Lambda$-parameter, $\Lambda_{\overline{\rm MS}}^{\rm eff}$. The latter needs to be extrapolated for $M \to \infty$ in order to extract the desired result, i.e.

$$\Lambda_{\overline{\rm MS}} = \Lambda_{\overline{\rm MS}}^{\rm eff} + \mathrm{O}(1/M^2) \,. \tag{68}$$

We shall now turn to the details of the numerical analysis taking the continuum values for the massive coupling as input (cf. table [1, Extended Data Table 3]).

The functions $F$ and $\chi_{0.36}$ can be determined from the results of [8] (see also appendix B of [24]). Here we list accurate numerical representations valid for the relevant couplings. The function $\chi_{0.36}$ is implicitly defined by its inverse function $\chi_{0.36}^{-1}$ via the expression

$$[\chi_{0.36}^{-1}(g)]^2 = \frac{g^2}{1 + g^2 P_{n_p}^{0.36}(g^2)} \,, \tag{69}$$

where $P_2^{0.36}$ is a second degree polynomial with coefficients

$$p_0 = -7.8231 \times 10^{-2} \,, \tag{70}$$
$$p_1 = 8.0579 \times 10^{-3} \,, \tag{71}$$
$$p_2 = -6.8506 \times 10^{-4} \,. \tag{72}$$

This polynomial is accurate for couplings $g^2 \in [3.8, 5.1]$.

The function $F(g)$ determines the $\Lambda_{\overline{\rm MS}}$-parameter in units of $\mu$ in the pure gauge theory as a function of $g(\mu)$ in the GF scheme. We use the numerical representation

$$F(g) = f_0 \times \exp\left\{\frac{q_0}{2}\left(\frac{1}{g_{\rm sw}^2} - \frac{1}{g^2}\right) + \frac{q_1}{2}\log\frac{g_{\rm sw}^2}{g^2} + \frac{q_2}{2}\left(g_{\rm sw}^2 - g^2\right) + \frac{q_3}{4}\left(g_{\rm sw}^4 - g^4\right)\right\}, \quad (73)$$

with parameters

$$g_{\rm sw}^2 = 4\pi \times 0.2\,, \quad f_0 = 0.2658(36)0.07974886\,, \quad q_0 = 14.93613381\,, \quad (74)$$
$$q_1 = -1.03947429\,, \quad q_2 = 0.18007512\,, \quad q_3 = -0.01437036\,. \quad (75)$$

This representation is valid for $g^2 \in [4\pi \times 0.2, 11]$. In this formula the parameter $f_0$ is the pure gauge $\Lambda-$parameter in units of a scale $\mu_{\rm ref}$. It is determined by matching non-perturbatively with the SF scheme, and a good perturbative behavior is observed (see [8, Section 3.5] for details).

In practice the set of parameters $\{p_0, p_1, p_2, f_0, q_0, q_1, q_2, q_3\}$ are all correlated, since they come from fits performed to data determined from a few common Monte-Carlo simulations. The reader interested in this covariance is invited to check our replication package [35, file `main/nf0_parameters.jl`].

These functions are applied to the second column of [1, Extended Data Table 3] to determine columns three and four.

The $z \to \infty$ extrapolation of $\rho^{\rm eff}$ is carried out by a fit to the leading behavior in the large mass expansion,

$$\rho^{\rm eff} = \rho + s\,[\alpha_s(m_\star)]^{\hat{\Gamma}_{\rm M}}\frac{1}{z^2}\,, \quad (76)$$

where, we recall, $m_\star$ is defined by $\overline{m}_{\overline{\rm MS}}(m_\star) = m_\star$. From the effective theory analysis of the large mass limit of QCD in ref. [24], we established that a sensible range for the exponent $\hat{\Gamma}_{\rm M}$ is $\hat{\Gamma}_{\rm M} \in [0,1]$ (cf. Section 4.5.2 of that reference). In order to quantify the sensitivity of the $z \to \infty$ extrapolations we consider the endpoints of this interval and take $\hat{\Gamma}_{\rm M} = 0, 1$. The results are found in [1, Extended Data Table 3]. Taking the more conservative fits to the data with $z \geq 6$ ($\hat{\Gamma}_{\rm M} = 0$) we obtain

$$\rho = 0.426(10)_{\rm stat}(4)_{\rm sys}(10)_{\rm tot}\,. \quad (77)$$

The corresponding large mass extrapolation is shown in [1, Extended Data Figure 2].

We end this section with a remark on the different systematics that affect our computation. Despite the fact that we work in a finite volume, using very small values of the lattice spacing ($a^{-1} \approx 10 - 50$ GeV, with $\alpha_s(a^{-1})$ varying very little), the continuum values of the massive coupling are affected by the choice of $\hat{\Gamma}_{\rm eff}, \hat{\Gamma}'_{\rm eff}$ (cf. eq. (65)). This illustrates how difficult continuum extrapolations can be. Fortunately, our final goal is not the determination of the massive coupling in the continuum itself, but rather the determination of $\Lambda^{(3)}_{\overline{\rm MS}}$. As shown in [1, Extended Data Figure 5], the effect on $\Lambda^{(3)}_{\overline{\rm MS}}$ due to the change in the coupling values for different continuum extrapolations

is very mild. Logarithmic corrections in the $M \to \infty$ extrapolation (cf. the parameter $\hat{\Gamma}_{\rm M}$ in eq. (76)) also have little effect on $\Lambda^{(3)}_{\overline{\rm MS}}$. All in all, our decoupling strategy has allowed us to extract $\Lambda$ with an error dominated by statistical uncertainties.

# 7 Charm and bottom contributions

## 7.1 Perturbative truncation errors

Our determination of the $\Lambda$ parameter in three flavor QCD still needs to be connected with the phenomenologically most relevant quantity: the strong coupling in the five flavor theory. This requires to take into account the effect of the *charm* and *bottom* quarks. Since $\Lambda/M \ll 1$ perturbation theory can be used to relate $\Lambda^{(3)}_{\overline{\rm MS}}$ with $\Lambda^{(4)}_{\overline{\rm MS}}$ and $\Lambda^{(5)}_{\overline{\rm MS}}$. As external input we use the quark masses [27] $m_{\rm c}^\star = 1275.0(5.0)$ MeV and $m_{\rm b}^\star = 4171(20)$ MeV. Their uncertainties are irrelevant in the following. Decoupling of a single flavour is naturally discussed matching the fundamental theory with $N_{\rm f}+1$ quark flavours to the one with $N_{\rm f}$ flavours, where, in the notation of [38], the ratio of the corresponding $\Lambda$-parameters is parameterized as

$$\Lambda^{(N_{\rm f})}_{\overline{\rm MS}} = P_{N_{\rm f},N_{\rm f}+1}\left(M/\Lambda^{(N_{\rm f}+1)}_{\overline{\rm MS}}\right)\Lambda^{(N_{\rm f}+1)}_{\overline{\rm MS}}\,. \tag{78}$$

Here we need to solve for $\Lambda^{(N_{\rm f}+1)}_{\overline{\rm MS}}$ and we shall denote the inverse $P$-function by $P_{N_{\rm f}+1,N_{\rm f}}$, where the argument is now $M/\Lambda^{(N_{\rm f})}$, with $M$ still defined as the mass of the decoupled quark in the $N_{\rm f}+1$ theory. The inverse equation for $\Lambda^{(N_{\rm f}+1)}_{\overline{\rm MS}}$ is therefore implicit, as the connection between the input parameter $m_\star$ and $M$ requires the quark mass running with $N_{\rm f}+1$ quark flavours and the corresponding $\Lambda$-parameter.

In the following we evaluate $P_{4,3}(M_{\rm c}/\Lambda^{(3)}_{\overline{\rm MS}})$ and $P_{5,4}(M_{\rm b}/\Lambda^{(4)}_{\overline{\rm MS}})$ in perturbation theory, in order to establish the connection to the five-flavour theory and $\alpha_s(m_Z)$. This assumes that perturbation theory is still reliable at the relatively low scales set by the bottom and charm quark masses, and we need to carefully assess the uncertainties incurred in these steps.

We use the perturbative results of Refs. [40, 41], where matching is discussed in terms of the $\overline{\rm MS}$ couplings. To simplify notation, we denote the coupling with $N_{\rm f}+1$ massless flavours by $\bar{g}(\mu)$ and the one with $N_{\rm f}$ flavours by $\bar{g}_-(\mu)$. Then the relation between the couplings can be written in the form

$$\bar{g}(\mu) = \xi_\star(\bar{g}_-(\mu), \mu/m_\star)\bar{g}_-(\mu), \tag{79}$$

where $m_\star$ is the input heavy quark mass, defined in the theory with $N_{\rm f}+1$ quarks. The matching function up to four-loop order ($k \leq 4$) is taken from Ref. [40],

$$\xi_\star^2(\bar{g}_-(\mu), \mu/m_\star) = 1 + \sum_{k\geq 1} \bar{g}_-^{2k}(\mu) \times d'_{{\rm SI},k}(\mu/m_\star)/(4\pi^2)^k\,, \tag{80}$$

and the coefficients[10], $d'_{\mathrm{SI},k}(\mu/m_\star)$, are polynomials of $\ln(\mu/m_\star)$ of order $k$. In order to estimate the uncertainty on the ratio of $\Lambda$-parameters, we write the $P$-function in the form,

$$P_{N_\mathrm{f}+1,N_\mathrm{f}} = \frac{\mu\varphi_{\overline{\mathrm{MS}}}^{(N_\mathrm{f}+1)}\left[\xi_\star(\bar{g}_-(\mu),\mu/m_\star)\times\bar{g}_-(\mu)\right]}{\Lambda_{\overline{\mathrm{MS}}}^{(N_\mathrm{f})}}\,, \tag{81}$$

which takes account of the fact that $\Lambda_{\overline{\mathrm{MS}}}^{(N_\mathrm{f})}$ is treated as input parameter. For our central values we set $\mu = m_\star$, which seems the most natural choice, given that $d'_{\mathrm{SI},1}(1) = 0$. Note that the use of the $\beta$-function at five-loop order goes together with the matching function $\xi_\star$ evaluated to four-loop order. Our reference values are obtained using all available orders and given in the first row of Table 1. Then, reducing the loop order in both the $\beta$-function and the matching function $\xi_\star$, we obtain the results given in Table 1, for both charm and bottom quarks. The last column shows the combined effects propagated to $\alpha_s(m_Z)$.

Regarding the charm threshold, the three-, four- and five-loop contributions to $P_{4,3}(M_\mathrm{c}/\Lambda_{\overline{\mathrm{MS}}}^{(3)})$ are respectively 3.9%, 0.85% and 0.25%. This shows that even at the charm scale, this particular perturbative series is well behaved with fast decreasing contributions. We will use the highest order contribution for the perturbative uncertainty, as is appropriate in an asymptotic series. Note that the series does not really show any sign that it is close to the point where its asymptotic behavior is relevant. Thus, taking the highest order as an uncertainty is very conservative. Nevertheless, we check this also by a second estimate, where we vary the scale $\mu$ at which the couplings are matched across the quark thresholds. We first set $\mu = sm_\star$ in Eq. (81), with the scale factor $s$ set to $1/2$ or 2, for the bottom quark threshold, $P_{5,4}$. As for the charm quark threshold we only vary the scale upwards ($s = 2$), as the charm quark scale is too low for a further reduction in scale. The percentage deviations of $P_{4,3}$ and $P_{5,4}$ from their reference values are given in Table 1.

Finally, in order to enable two-sided scale variations also for the charm quark, we considered a variant of scale variation in terms of the running mass in the $\overline{\mathrm{MS}}$-scheme at scale $\mu$. Eq. (81) is now re-written,

$$P_{N_\mathrm{f}+1,N_\mathrm{f}} = \frac{\mu\varphi_{\overline{\mathrm{MS}}}^{(N_\mathrm{f}+1)}\left[\xi(\bar{g}_-(\mu),\mu/\bar{m}(\mu))\times\bar{g}_-(\mu)\right]}{\Lambda_{\overline{\mathrm{MS}}}^{(N_\mathrm{f})}} \tag{82}$$

where the required matching function $\xi$,

$$\xi^2(\bar{g}_-(\mu),\mu/\bar{m}(\mu)) = 1 + \sum_{k\geq 1}\bar{g}_-^{2k}(\mu)\times d'_{\mathrm{MS},k}(\mu/\bar{m}(\mu))/(4\pi^2)^k\,, \tag{83}$$

now comes with perturbative coefficients, $d'_{\mathrm{MS},k}(\mu/\bar{m}(\mu))$, which are given up to four-loop order ($k \leq 4$) in ref. [40]. Note that the arguments of $\xi$ involve the running mass of the heavy quark in QCD with $N_\mathrm{f}+1$ quark flavours. Its scale evolution thus requires the input of $\Lambda^{(N_\mathrm{f}+1)}$, which is also the target quantity. In principle, this circularity

[10]We here use the notation from [40], where SI stands for the "scale invariant quark mass", i.e. $m_\star$ in our notation.

| loop-orders | $P^{\text{ref}}_{4,3}$ | $P^{\text{ref}}_{5,4}$ | $P^{\text{ref}}_{5,3}$ | $\alpha^{\text{ref}}_s(m_Z)$ |
|---|---|---|---|---|
| 5/4 | 0.87548 | 0.72143 | 0.63160 | 0.11872 |
| loop-orders | $100 \times \delta P_{4,3}$ | $100 \times \delta P_{5,4}$ | $100 \times \delta P_{5,3}$ | $10^5 \times \Delta\alpha_s(m_Z)$ |
| **4/3** | **−0.2536** | **−0.0485** | **−0.3313** | **−5.992** |
| 3/2 | −0.8503 | −0.2793 | −1.2237 | −22.20 |
| 2/1 | −3.8555 | −2.6798 | −6.8235 | −126.3 |
| SI, $m_\star$ | 0.0 | 0.0 | | |
| SI, $2m_\star$ | −0.4364 | −0.0702 | | |
| SI, $m_\star/2$ | | −0.0117 | | |
| $\overline{\text{MS}}$, $\mu = \mu_h$ | −0.0299 | −0.0014 | | |
| $\overline{\text{MS}}$, $\mu = 2\mu_h$ | −0.1036 | −0.0105 | | |
| $\overline{\text{MS}}$, $\mu = \mu_h/2$ | 0.0016 | 0.0119 | | |

**Supplementary Table** 1: Perturbative uncertainties in the matching of the 3-flavour $\overline{\text{MS}}$-coupling over the charm and bottom thresholds. We recall that the crucial quantities for the analysis are the functions $P_{N_f+1,N_f}$ for $N_f = 3, 4$ (see section 7.1 for more details). Results are given as relative deviations from the reference values in the first row, e.g. $\delta P_{4,3} = (P_{4,3} - P^{\text{ref}}_{4,3})/P^{\text{ref}}_{4,3}$, or as the absolute deviation $\Delta\alpha_s = \alpha_s - \alpha^{\text{ref}}_s$. The quark masses $m_\star$ are set to the relevant input charm and bottom quark masses, $m_c = 1.275\,\text{GeV}$ and $m_b = 4.171\,\text{GeV}$, and the scales $\mu_h$ are set to $\mu_c = 3\,\text{GeV}$ and $\mu_b = 5\,\text{GeV}$, respectively. The values for $\Lambda^{(4/5)}_{\overline{\text{MS}}}$ required for the scale evolution of the running mass are set to $\Lambda^{(3)}_{\overline{\text{MS}}} \times P^{\text{ref}}_{4/5,3}$, with input $\Lambda^{(3)}_{\overline{\text{MS}}} = 343.8\,\text{MeV}$. When calculating $P_{5,3}$, the denominator is set to $\Lambda^{(3)}_{\overline{\text{MS}}}$ times $P_{4,3}$ rather than the reference value $P^{\text{ref}}_{4,3}$, so that $P_{5,3}$ differs from the product $P_{4,3} \times P_{5,4}$, except for the reference procedure. To obtain $\alpha_s(m_Z)$ we solve $\Lambda^{(5)}_{\overline{\text{MS}}}/m_Z = \varphi^{(5)}_{\overline{\text{MS}}}(\sqrt{4\pi\alpha_s(m_Z)})$, using the five-loop $\beta$-function with $N_f = 5$ in the $\overline{\text{MS}}$-scheme. For the perturbative uncertainties in the $P$-factors and $\alpha_s$ we use the row in bold.

calls for an iterative solution, however, the numerical sensitivity of the coupling on the quark mass is so small that a good guess is sufficient for our purposes. For definiteness, we take the values for $\Lambda^{(4)}_{\overline{\text{MS}}}$ and $\Lambda^{(5)}_{\overline{\text{MS}}}$ from our reference procedure for the matching. For the central scale values we choose $\mu_h = 3\,\text{GeV}$ for the charm quark, and $\mu_h = 5\,\text{GeV}$ for the bottom quark. Then, setting $\mu = s\mu_h$ we first obtain $\bar{g}(m_\star)$ and $\bar{g}(s\mu_h)$ for $s \in \{1/2, 1, 2\}$. This then allows us to convert the input values for $m_\star$ to $\overline{m}(s\mu_h)$ (cf. Section 9 for the running of quark masses). Computing the values of $\bar{g}_-(\mu)$ at the same scales, the arguments to be inserted into $\varphi^{(N_f+1)}_{\overline{\text{MS}}}$ in Eq. (82) are known and the results for the scale variations are collected in Table 1.

Out of these different variations, we use the last term in the series as estimate of the *perturbative* truncation uncertainty. It is one order of magnitude below the total uncertainty due to $\Lambda^{(3)}_{\overline{\text{MS}}}$. Of all the quoted uncertainties in table 1, only the scale

variation uncertainty at scale $2m_\star$ gives a somewhat larger uncertainty (0.4% versus 0.3%), but this difference is hardly noticeable in our result for $\Lambda^{(4)}$ and $\alpha_s(m_Z)$.

The case of the bottom quark is very similar, except that the significantly higher scale implies even smaller uncertainties. In particular, the three-, four- and five-loop contributions in $P_{5,4}$ are 2.7%, 0.3% and 0.04%, respectively. In this case a single scale variation estimate is larger than the highest loop order contribution, namely 0.07%, but such differences are completely negligible at our level of precision.

## 7.2 Non-perturbative *charm* contributions

Having addressed the perturbative truncation uncertainties, we have to consider the possibility of non-perturbative contributions. It is expected that these are dominantly due to the next higher dimensional operator in the decoupled effective theory and are thus accompanied by a $1/M^2$ suppression factor. Thus, for bottom quarks this suppression is expected to be one order of magnitude stronger than the the charm quark, so that we will focus just on the charm quark.

Hence, we are interested in the difference between a computation of $\sqrt{8t_0} \times \Lambda^{(3)}_{\overline{\rm MS}} \times P_{4,3}$, using the perturbative evaluation of $P_{4,3}$, and a direct computation in the four-flavour theory of $\sqrt{8t_0} \times \Lambda^{(4)}_{\overline{\rm MS}}$. First note that our range for $\sqrt{t_0}$ includes all precise three- and four-flavour computations (see Section 3). This means that charm effects in quantities typically used for scale setting ($\sqrt{8t_0} \times M_\Omega$ or $\sqrt{8t_0} \times f_\pi$) are below our current quoted precision in the scale. Furthermore this finding agrees with the detailed investigations of sea-quark effects of two dynamical quarks with the mass of a charm quark [55, 56]. In the model where only the heavy quarks are present, ratios of low energy scales were shown to agree with the completely decoupled theory, i.e. pure gauge theory, up to very small power corrections of a few ‰.

Could the case of $\sqrt{8t_0} \times \Lambda$ be significantly different? This question was studied in the same model in Ref. [38]. The uncertainty in quantities like $\sqrt{t_0} \times \Lambda$ was found to be 0.2% per quark at the charm mass. The estimate resulted from a rather conservative analysis.

Here we present a somewhat different, more direct, analysis for the scales $\mathcal{S}(M) = \sqrt{t_0(M)}, w_0(M)$ in two flavor QCD. We follow the lines of our decoupling strategy (see Section 6) to write

$$\mathcal{S}(M) \times \Lambda^{(2)}_{\overline{\rm MS}} = (\mathcal{S} \times \Lambda^{(0)}_{\overline{\rm MS}}) \times \frac{1}{P_{0,2}(M/\Lambda^{(2)})} \times (1 + \mathcal{O}(\Lambda^2/M^2))\,, \qquad (84)$$

where $\mathcal{S}$ is either $\sqrt{t_0}$ or $w_0$ in the pure gauge theory. In order to gain precision, it is convenient to multiply both sides of Eq. (84) by the ratio

$$\frac{L_1}{S \times \Lambda^{(0)}_{\overline{\rm MS}}}\,, \qquad (85)$$

| $M/\Lambda^{(2)}$ | $1/P_{0,2}(\Lambda/M)$ | $\sqrt{t_0(M)}/L_1$ | $w_0(M)/L_1$ |
|---|---|---|---|
| 1.2800 | 1.230494 | 0.3284(50) | 0.3448(54) |
| 2.5000 | 1.204202 | 0.3064(46) | 0.3170(49) |
| 4.8700 | 1.107274 | 0.2828(48) | 0.2897(49) |
| 5.7781 | 1.025934 | 0.2775(43) | 0.2840(47) |

**Supplementary Table** 2: Values of reference scales $\sqrt{t_0(M)}, w_0(M)$ in two flavor QCD with heavy quarks $M$ in units of a reference scale $L_1$. These values can be used to study the non-perturbative effects in the decoupling of the charm quark (see section 7.2 and figure 1)

where $L_1$, defined in [38], is a scale of the massless theory. With a trivial rearrangement of the terms, one finds the relation

$$\frac{L_1}{S(M) \times P_{0,2}(M/\Lambda^{(2)})} = \frac{\Lambda^{(2)}_{\overline{\mathrm{MS}}} \times L_1}{\Lambda^{(0)}_{\overline{\mathrm{MS}}} \times S} \times (1 + \mathcal{O}(\Lambda^2/M^2)) \,. \tag{86}$$

Note that the power corrections in Eq. (86) are exactly the same as in Eq. (84) since the factor Eq. (85) does not depend on $M$. The data allows us to estimate the l.h.s. of Eq. (86), where deviations from a constant are the seeked power corrections.

The data displayed in figure 1 agrees with expectations. A single term $\mathcal{O}(\Lambda^2/M^2)) \to 2k_{\mathcal{S}}\,\Lambda^2/M^2$ is sufficient and its magnitude provides information on the non-perturbative corrections due to the simultaneous decoupling of two quarks. The lines in Figure 1 correspond to very small $k_{\mathcal{S}}$: at the charm quark mass we have $k_{\sqrt{t_0}}\,\Lambda^2/M_c^2 = 0.043(37)\%$ and $k_{w_0}\,\Lambda^2/M_c^2 = -0.046(39)\%$. Non-perturbative corrections to decoupling in low energy quantities are very small.

The caveat of this investigation is that we obtain the numbers for $k_{\mathcal{S}}$ from the region of the charm mass and below. For this reason we replace the 0.5‰ estimate by 1‰, which is still much smaller that the perturbative uncertainty in $P_{4,3}$ estimated above. We will just ignore it in the following, just as the non-perturbative bottom contribution which is entirely negligible.

# 8 Λ parameters and the strong coupling

The two strategies that we employed for extracting $\Lambda^{(3)}_{\overline{\mathrm{MS}}}$ are largely independent, and are affected by very different systematics. First we quote the results of $\Lambda^{(3)}_{\overline{\mathrm{MS}}}$ in units of $\sqrt{t_0}$ determined using each strategy

$$\text{Massless running: } \sqrt{8t_0} \times \Lambda^{(3)}_{\overline{\mathrm{MS}}} = 0.713(18)_{\mathrm{stat}}(12)_{\mathrm{sys}}(21)_{\mathrm{tot}} \,, \tag{87}$$

$$\text{Decoupling: } \sqrt{8t_0} \times \Lambda^{(3)}_{\overline{\mathrm{MS}}} = 0.703(17)_{\mathrm{stat}}(8)_{\mathrm{sys}}(19)_{\mathrm{tot}} \,. \tag{88}$$

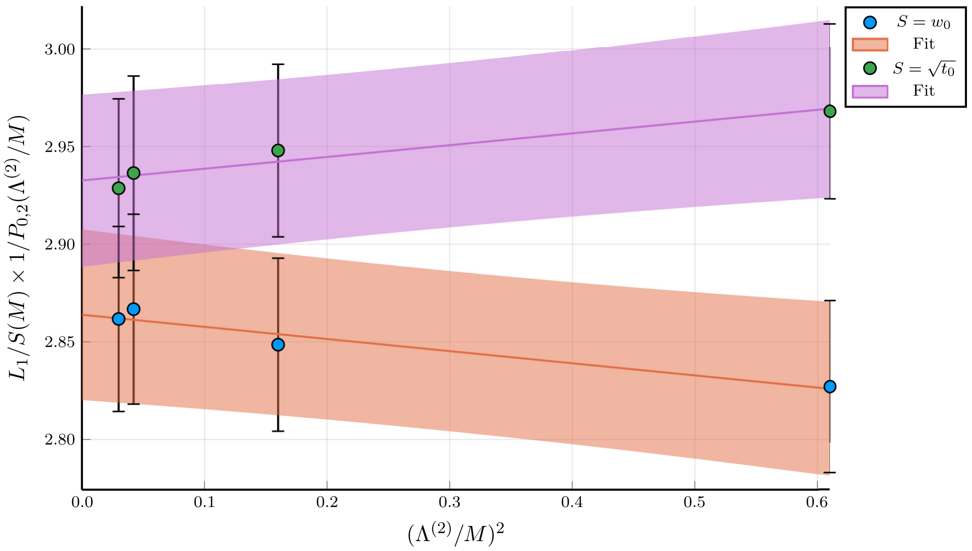


**Supplementary Figure** 1: When heavy quarks decouple in $\sqrt{8t_0} \times \Lambda$, one expects non-perturbative corrections $\mathcal{O}(\Lambda^2/M^2)$. In this plot an explicit computation using an extended dataset of [38] was performed. The quantity $L_1/S(M) \times 1/P_{0,2}(M/\Lambda^{(2)})$ approaches as $M \to \infty$ the quantity $\frac{\Lambda^{(2)}_{\overline{\rm MS}} \times L_1}{\Lambda^{(0)}_{\overline{\rm MS}} \times S}$ after the simultaneous decoupling of 2 heavy quarks in two flavor QCD. The size of the corrections shows that these non-perturbative corrections are very small, about 0.05(5)% per quark at the charm quark mass. The figure shows not only the case of $\sqrt{8t_0} \times \Lambda$ but also of the related flow scale $w_0 \times \Lambda$, with a similar behavior, pointing to the fact that these non-perturbative charm corrections are small for most common scale-setting quantities. In the end we use an estimate of the non-perturbative charm corrections of 0.1% (a factor two larger than the correction estimated here), to account for any possible modeling of the uncertainties. Still this contributes less than 1% to our total error squared in $\alpha_s$ (see [1, Figure 4]).

Using our estimate for $\sqrt{t_0}$ (cf. section 3) we obtain:

$$\text{Massless running:} \quad \Lambda^{(3)}_{\overline{\rm MS}} = 347(9)_{\rm stat}(6)_{\rm sys}(4)_{\rm robust}(11)_{\rm tot}\,\text{MeV}\,, \tag{89}$$

$$\text{Decoupling:} \quad \Lambda^{(3)}_{\overline{\rm MS}} = 342(9)_{\rm stat}(4)_{\rm sys}(4)_{\rm robust}(10)_{\rm tot}\,\text{MeV}\,. \tag{90}$$

The results show a good agreement. The Pearson correlation between the two estimates of $\Lambda^{(3)}_{\overline{\rm MS}}$ is 0.33. Since the uncertainties are dominated by statistics and have very different origin, an average is justified. A correlated fit to a constant leads to our final result

$$\sqrt{8t_0} \times \Lambda^{(3)}_{\overline{\rm MS}} = 0.708(14)_{\rm stat}(8)_{\rm sys}(16)_{\rm tot}\,, \tag{91}$$

$$\Lambda_{\overline{\rm MS}}^{(3)} = 344.4(6.8)_{\rm stat}(3.7)_{\rm sys}(4.0)_{\rm robust}(8.7)_{\rm tot}\,\text{MeV}\,. \tag{92}$$

Our result for the three flavor $\Lambda$ parameter can be converted to the four and five flavor ones by crossing the *charm* and *bottom* thresholds perturbatively

$$\Lambda_{\overline{\rm MS}}^{(4)} = 301.5(6.7)_{\rm stat}(3.8)_{\rm sys}(4.0)_{\rm robust}(8.7)_{\rm tot} \quad \text{MeV}\,, \tag{93}$$
$$\Lambda_{\overline{\rm MS}}^{(5)} = 217.6(5.4)_{\rm stat}(3.1)_{\rm sys}(3.2)_{\rm robust}(7.0)_{\rm tot} \quad \text{MeV}\,. \tag{94}$$

It is worth reiterating that we have been quite conservative in our assignment of uncertainty for the conversion of our three flavor result to the four/five flavor numbers. Our in-depth analysis suggests significantly smaller effects, particularly our estimate for non-perturbative effects (figure 1). Crucially, these uncertainties have a negligible impact on the second decimal place of our uncertainties.

Finally using the experimental input $m_Z = 91188.0(2.0)$ MeV [57] one determines $\alpha_s(m_Z)$ by solving the equation

$$\frac{\Lambda_{\overline{\rm MS}}^{(5)}}{m_Z} = \varphi_{\overline{\rm MS}}^{(N_{\rm f}=5)}\left(\sqrt{\alpha_s(m_Z)\times(4\pi)}\right)\,. \tag{95}$$

Our result

$$\alpha_s(m_Z) = 0.11876(45)_{\rm stat}(25)_{\rm sys}(27)_{\rm robust}(58)_{\rm tot}\,, \tag{96}$$

shows a remarkable precision, still the uncertainties are dominated by the uncertainty in $\Lambda_{\overline{\rm MS}}^{(3)}$ (eq. (91)). The two main sources of uncertainty are the running in the pure gauge theory and the scale $\sqrt{t_0}$, each contributing approximately 25% to the error squared in $\alpha_s(m_Z)$. The massless running at high energies (eq. (47)) contributes 16% of the error squared, and the dimensionless ratio $\sqrt{t_0^\star}\mu_{\rm had}$ (eq. (32)) contributes 10%. The rest of the uncertainty in our final result comes from the statistical uncertainties in the determination of the massive couplings.

# 9 Mass renormalization

In the decoupling strategy, the continuum limit is taken at some prescribed values of the renormalization-group invariant (RGI) quark mass, $z = M/\mu_{\rm dec} = 4, 6, \ldots, 12$, measured in units of the scale $\mu_{\rm dec}$ at which we apply decoupling (cf. Section 6). On the lattice, this is achieved by a proper tuning of the bare quark-mass parameter $m_0$ appearing in the lattice Lagrangian as a function of the lattice cutoff $1/a$. More precisely, for the lattice discretization employed in this work, the relation between the RGI and bare quark mass reads[11]

$$M = Z_{\rm M}(\tilde g_0^2)\widetilde m_{\rm q}\,, \qquad \widetilde m_{\rm q} = m_{\rm q}(1 + b_{\rm m}(\tilde g_0^2)am_{\rm q})\,, \qquad m_{\rm q} = m_0 - m_{\rm crit}\,. \tag{97}$$

In this equation, $Z_{\rm M}(\tilde g_0^2)$ is the renormalization factor that relates the so called improved bare subtracted quark mass $\widetilde m_{\rm q}$ and the RGI mass $M$. It only depends on

[11] We recall that $\tilde g_0^2$ stands for the so called improved bare coupling (cf. Section 6) and a given value of $\tilde g_0^2$ corresponds to a particular value of the lattice cutoff $1/a$.

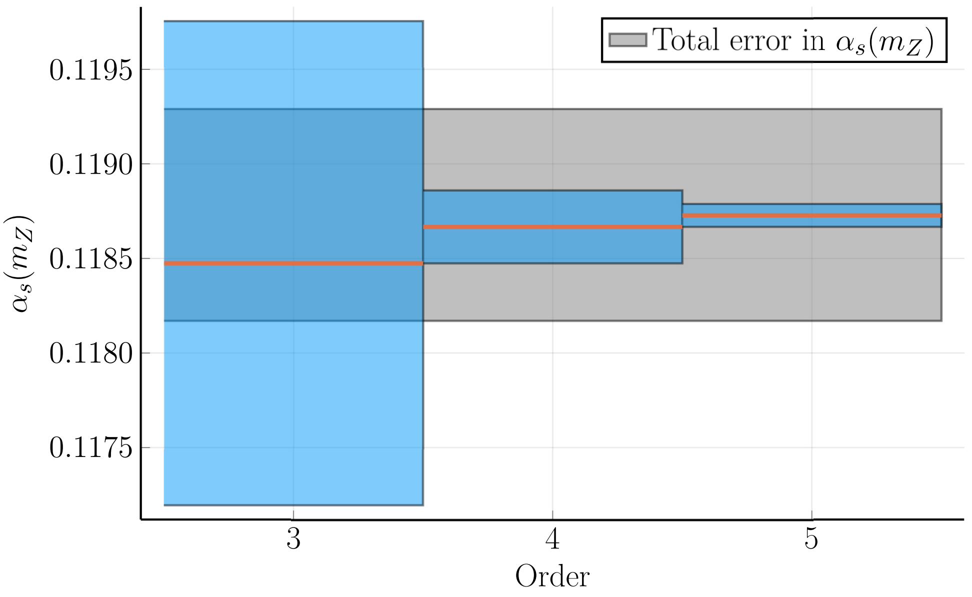


**Supplementary Figure** 2: Translating the value of $\Lambda^{(3)}_{\overline{\rm MS}}$ into the value of the strong coupling requires crossing the charm and bottom quark thresholds using perturbation theory. This plot shows the perturbative uncertainties at each order $n = 3, 4, 5$. Orange horizontal lines represent $\alpha_s(m_Z)$ extracted from our central value $\Lambda^{(3)}_{\overline{\rm MS}} = 343.8$ MeV using different orders in perturbation theory. The bands represent the perturbative uncertainties, estimated by the difference between the order $n$ and the order $n-1$ result. These perturbative estimates seem very conservative: for $n = 3$ and $n = 4$ the error bars are several times larger than the actual difference between the order $n$ and the order $n+1$. Still the perturbative uncertainty for $n = 5$ contributes less than 2% in the final error squared of $\alpha_s(m_Z)$ (see [1, Figure 4]).

the value of the lattice cutoff through $\tilde{g}_0^2$, while, as $M$ itself, it is renormalization scheme and scale independent. The coefficient function $b_{\rm m}(\tilde{g}_0^2)$ appearing in $\widetilde{m}_{\rm q}$ is analogous to the function $b_{\rm g}(\tilde{g}_0^2)$ entering the definition of the improved bare coupling $\tilde{g}_0^2$: if properly tuned, it allows for the removal of $\mathrm{O}(a)$ discretization errors in the definition of the renormalized quark mass [19].[12] Given $b_{\rm m}(\tilde{g}_0^2)$, the improved mass $\widetilde{m}_{\rm q}$ is related to the subtracted quark mass $m_{\rm q}$ by a quadratic equation. The bare mass $m_{\rm q}$ is defined in terms of the parameter $m_0$ that controls the simulations and the critical mass parameter $m_{\rm crit} \equiv m_{\rm crit}(g_0^2)$, which identifies the value of $m_0$ for which (up to discretization errors) the quarks are massless.

In ref. [24], the functions $Z_{\rm M}(\tilde{g}_0^2)$, $b_{\rm m}(\tilde{g}_0^2)$, and $m_{\rm crit}(g_0^2)$, have been determined for the values of the lattice spacing of interest from the results for $Z_{\rm m}$, $b_{\rm m}$, and $am_{\rm crit}$ collected in Table 3. The renormalization factor $Z_{\rm M}$ is determined via the introduction

[12] We note that differently from ref. [19], we take as an argument of the function $b_{\rm m}$ the improved bare coupling $\tilde{g}_0^2$ instead of $g_0^2$. The difference between these two choices amounts to an $\mathrm{O}((am_{\rm q})^2)$ effect in $M$.

| $L/a$ | $\beta$ | $am_{\rm crit}$ | $Z_{\rm m}$ | $b_{\rm m}$ |
|---|---|---|---|---|
| 12 | 4.3020 | −0.323417(38) | 1.6882(72) | −0.42(20) |
| 16 | 4.4662 | −0.312928(23) | 1.7252(80) | −0.50(12) |
| 20 | 4.5997 | −0.304289(24) | 1.739(10) | −0.47(14) |
| 24 | 4.7141 | −0.296941(14) | 1.770(11) | −0.51(10) |
| 32 | 4.9000 | −0.285427(12) | 1.813(17) | −0.619(48) |
| 40 | 5.0671 | −0.275473(11) | 1.807(19) | −0.50(10) |
| 48 | 5.1739 | −0.2693605(82) | 1.823(22) | −0.528(73) |

**Supplementary Table** 3: Results for $am_{\rm crit}$, $Z_{\rm m} \equiv Z_{\rm m}^{\rm SF}(g_0^2, a\mu_{\rm dec})$, and $b_{\rm m}$, for different values of $L/a$ and $\beta = 6/g_0^2$. See the main text for any unexplained notation.

of an intermediate, mass-independent, renormalization scheme $r$ for the quark mass, according to the equation

$$Z_{\rm M}(\tilde{g}_0^2) = \left(\frac{M}{\overline{m}_r(\mu_{\rm dec})}\right) Z_{\rm m}^r(\tilde{g}_0^2, a\mu_{\rm dec})\,. \tag{98}$$

In this equation, we denoted with $Z_{\rm m}^r(\tilde{g}_0^2, a\mu_{\rm dec})$ the renormalization constant that defines the corresponding renormalized, running, quark mass,

$$\overline{m}_r(\mu_{\rm dec}) = \lim_{a\to 0} Z_{\rm m}^r(\tilde{g}_0^2, a\mu_{\rm dec})\widetilde{m}_{\rm q}\,, \tag{99}$$

in the renormalization scheme $r$ and at the renormalization scale $\mu = \mu_{\rm dec}$.

In order to compute the first factor on the r.h.s. of eq. (98), one needs to determine, in the continuum limit, the RG evolution of the quark mass $\overline{m}_r(\mu)$ from the scale $\mu_{\rm dec}$, up to infinite energy. The RG running of the quark mass is encoded in the function $\varphi_{r,{\rm m}}(\bar{g}_s)$ that defines the RGI quark mass through,[13]

$$M = \overline{m}_r(\mu)\varphi_{r,{\rm m}}(\bar{g}_s(\mu))\,, \tag{100}$$

$$\varphi_{r,{\rm m}}(\bar{g}_s) = \left[2b_0\bar{g}_s^2\right]^{-\frac{d_0}{2b_0}} \exp\left\{-\int_0^{\bar{g}_s}\left[\frac{\tau_r(x)}{\beta_s(x)} - \frac{d_0}{b_0 x}\right]dx\right\}\,. \tag{101}$$

Here $\beta_s$ is the RG-function of the gauge coupling in the scheme $s$ introduced earlier (cf. eq. ([1].5)), while

$$\tau_r(\bar{g}_s(\mu)) = \frac{{\rm d}\ln\overline{m}_r(\mu)}{{\rm d}\ln\mu}\,, \tag{102}$$

determines the scale dependence of the quark mass in the scheme $r$. At high energy, $\mu \to \infty$, the function $\tau_r(\bar{g}_s(\mu))$ admits a perturbative expansion in powers of $\bar{g}_s^2$, where

[13] For the ease of notation we label the function $\varphi_{r,{\rm m}}(\bar{g}_s)$ only with the scheme $r$ defining the renormalized quark masses, although implicitly its form depends also on the scheme $s$ chosen for its argument, the gauge coupling. The same is true for the $\tau$-function. On the other hand, note that if either of these functions is expressed as a function of the renormalization scale $\mu$ in physical units, rather than the coupling $\bar{g}_s^2$, they only depend on $r$ (cf. e.g. ref. [58]).

the leading order coefficient $d_0$ is scheme independent, i.e.

$$\tau_r(\bar{g}_s) \overset{\bar{g}_s\to 0}{\sim} -\bar{g}_s^2(d_0 + d_1^{r,s}\bar{g}_s^2 + \ldots)\,, \qquad d_0 = 6C_F/(4\pi)^2\,. \tag{103}$$

From these definitions it is easy to show that the RGI mass $M$ is renormalization scheme and scale independent and, thus, satisfies,

$$M = \overline{m}_r(\mu)\varphi_{r,\mathrm{m}}(\bar{g}_s(\mu)) = \overline{m}_{r'}(\mu')\varphi_{r',\mathrm{m}}(\bar{g}_{s'}(\mu'))\,. \tag{104}$$

We moreover note that from eq. (100), it is immediate to infer that the ratio of quark masses at two different scales, $\mu_1, \mu_2$, can be written as

$$\frac{\overline{m}_r(\mu_2)}{\overline{m}_r(\mu_1)} = \exp\left\{\int_{\bar{g}_s(\mu_1)}^{\bar{g}_s(\mu_2)} \frac{\tau_r(x)}{\beta_s(x)}\,dx\right\}\,. \tag{105}$$

Similarly to the determination of the $\Lambda$-parameter (cf. [1, Methods]), also the ratio $M/\overline{m}_r(\mu_{\mathrm{dec}})$ is obtained by breaking up the computation of the RG running of the quark masses into two separate factors, each one referring to a different energy range,

$$\frac{M}{\overline{m}_r(\mu_{\mathrm{dec}})} = \frac{M}{\overline{m}_r(\mu_0/2)} \times \frac{\overline{m}_r(\mu_0/2)}{\overline{m}_r(\mu_{\mathrm{dec}})}\,, \tag{106}$$

where $\mu_0 = 4381(93)\,\mathrm{MeV}$ is the renormalization scale already introduced in eq. ([1].21).

For the first term in eq. (106), we find [58],

$$\frac{M}{\overline{m}_{\mathrm{SF}}(\mu_0/2)} = 1.7505(89)\,. \tag{107}$$

The determination relies on finite-volume renormalization schemes for both the quark masses and the gauge coupling based on the Schrödinger functional (SF) of QCD. It is obtained by considering eq. (100) and the following parameterizations for the RG-functions [58],

$$\tau_{\mathrm{SF}}(\bar{g}_{\mathrm{SF}}) = -\bar{g}_{\mathrm{SF}}^2 \sum_{n=0}^{2} d_n\, \bar{g}_{\mathrm{SF}}^{2n} \qquad \beta_{\mathrm{SF}}(\bar{g}_{\mathrm{SF}}) = -\bar{g}_{\mathrm{SF}}^3 \sum_{n=0}^{3} b_n\, \bar{g}_{\mathrm{SF}}^{2n} \qquad \bar{g}_{\mathrm{SF}}^2 \in [0, 2.45] \tag{108}$$

where the coefficients $b_0, b_1, b_2$, and $d_0, d_1$ are fixed to their perturbative values, with

$$d_1^{\mathrm{SF}} = \frac{1}{(4\pi)^2}(0.2168 + 0.084N_{\mathrm{f}})\,, \tag{109}$$

$$b_2^{\mathrm{SF}} = \frac{1}{(4\pi)^3}(0.483 - 0.275N_{\mathrm{f}} + 0.0361N_{\mathrm{f}}^2 - 0.00175N_{\mathrm{f}}^3)\,, \tag{110}$$

scheme dependent, while

$$(4\pi)^3 d_2^{\rm fit} = -0.18(52)\,, \qquad (4\pi)^4 b_3^{\rm fit} = 4(3)\,, \tag{111}$$

were inferred from the data. The value of the gauge coupling corresponding to the scale $\mu = \mu_0/2$ is given instead by (remember that $\mu_0$ was defined by $\bar{g}^2_{\rm SF}(\mu_0) = 2.012$) [6],

$$\bar{g}^2_{\rm SF}(\mu_0/2) = 2.452(11)\,. \tag{112}$$

For the second factor in eq. (106), we find,

$$\frac{\overline{m}_{\rm SF}(\mu_0/2)}{\overline{m}_{\rm SF}(\mu_{\rm dec})} = 0.8423(23). \tag{113}$$

It is obtained considering eq. (105) and the following parameterization of the ratio of RG-functions [58]

$$\frac{\tau_{\rm SF}(\bar{g}_{\rm GF})}{\beta_{\rm GF}(\bar{g}_{\rm GF})} = \frac{1}{\bar{g}_{\rm GF}} \sum_{n=0}^{3} f_n \bar{g}_{\rm GF}^{2n}\,, \tag{114}$$

with

$$f_0 = 1.28493\,, \quad f_1 = -0.292465\,, \quad f_2 = 0.0606401\,, \quad f_3 = -0.00291921\,. \tag{115}$$

These parameters are correlated with covariance,

$$\mathrm{cov}(f_i, f_j) = \begin{pmatrix} 2.33798\times10^{-2} & -1.47011\times10^{-2} & 2.81966\times10^{-3} & -1.66404\times10^{-4} \\ -1.47011\times10^{-2} & 9.54563\times10^{-3} & -1.87752\times10^{-3} & 1.12962\times10^{-4} \\ 2.81966\times10^{-3} & -1.87752\times10^{-3} & 3.78680\times10^{-4} & -2.32927\times10^{-5} \\ -1.66404\times10^{-4} & 1.12962\times10^{-4} & -2.32927\times10^{-5} & 1.46553\times10^{-6} \end{pmatrix}. \tag{116}$$

The relevant values of the gauge coupling are given by (cf. section 4 and [21, 24]),

$$\bar{g}^2_{\rm GF}(\mu_0/2) = 2.6723(64)\,, \qquad \bar{g}^2_{\rm GF}(\mu_{\rm dec}) = 3.949\,. \tag{117}$$

Combining the results above, we finally obtain,

$$\frac{M}{\overline{m}_{\rm SF}(\mu_{\rm dec})} = 1.4744(85). \tag{118}$$

We refer the interested reader to [24] and references therein for more details on the determinations presented in this section.

We conclude by noticing that our analysis properly takes into account the uncertainties on $Z_{\rm m}$, $b_{\rm m}$, $am_{\rm crit}$ (and their correlation) as well as those on $M/\overline{m}_{\rm SF}(\mu_{\rm dec})$ in the determination of $z = M/\mu_{\rm dec}$. These errors are propagated to the massive coupling $\bar{g}^2_{\rm GFT}(\mu_{\rm dec}, M)$ and finally to our result for $\Lambda^{(3)}_{\overline{\rm MS}}$ from decoupling. The uncertainty on $z$ contributes less than a 0.5% to the final error squared of $\Lambda^{(3)}_{\overline{\rm MS}}$ eq. ([1].3).

# 10 Boundary contributions to the decoupling limit

The GF coupling with SF boundary conditions employed in our decoupling strategy (cf. eq. (55)) is in principle affected by $\mathrm{O}(1/M)$ corrections to the large-mass limit. We can obtain an explicit expression for these contributions by invoking the tools of effective field theory.

## 10.1 Effective decoupling theory and O(1/M) terms

Focusing on the continuum theory, at large quark mass, QCD with $N_\mathrm{f}$ mass-degenerate quarks can be described by an effective theory with action [38, 59],

$$S_\mathrm{dec} = S_{0,\mathrm{dec}} + \frac{1}{m} S_{1,\mathrm{dec}} + \frac{1}{m^2} S_{2,\mathrm{dec}} + \dots \,, \tag{119}$$

where for the time being $m$ refers to a generic quark mass to be specified later. Since all quarks decouple simultaneously, the leading term $S_{0,\mathrm{dec}}$ corresponds to the pure gauge action,

$$S_{0,\mathrm{dec}} = -\frac{1}{2g^2} \int d^4x \; \mathrm{tr}\,(F_{\mu\nu} F_{\mu\nu})\,, \tag{120}$$

where $F_{\mu\nu}$ is the Yang-Mills field-strength tensor and $g$ a yet unspecified gauge coupling. The other terms, $S_{k,\mathrm{dec}}$ with $k > 0$, are given by space-time integrals of gauge invariant local operators, polynomial in the gauge field and its derivatives. In the case of a theory with no space-time boundary these are of mass dimension $4 + k$. Moreover, gauge invariance and $O(4)$ symmetry do not allow for odd values of $k$, so that the $S_{1,\mathrm{dec}}$ term must vanish.

Different is the situation in the presence of SF boundary conditions [15, 16]. In this case, additional terms are allowed in the effective action (119). The $S_{k,\mathrm{dec}}$ terms can in fact include also space integrals of dimension $3+k$ operators localized at $x_0 = 0, T$. Considering the homogeneous SF boundary conditions employed in our strategy and the symmetries of the resulting theory, one can show that we can now have a linear term in $1/m$ of the form [24]

$$S_{1,\mathrm{dec}} = \int d^3x \, \omega_\mathrm{b}(g) \big[\mathcal{O}_\mathrm{b}(0,\boldsymbol{x}) + \mathcal{O}_\mathrm{b}(T,\boldsymbol{x})\big]\,, \quad \mathcal{O}_\mathrm{b}(x) = -\frac{1}{g^2}\sum_{k=1}^{3} \mathrm{tr}\,(F_{0k}(x) F_{0k}(x))\,. \tag{121}$$

In this equation, $\omega_\mathrm{b}(g)$ is a coefficient function that needs to be properly adjusted in order to match the results of QCD with $N_\mathrm{f}$ heavy quarks and those of the effective theory up to $\mathrm{O}(1/m^2)$ corrections. Its value has been computed at 1-loop order in perturbation theory in ref. [24], and it is given by[14]

$$\omega_\mathrm{b}(g) = \omega_\mathrm{b}^{(1)} g^2 + \omega_\mathrm{b}^{(2)} g^4 + \dots\,, \qquad \omega_\mathrm{b}^{(1)} = -0.0541(5) N_\mathrm{f}/(4\pi)\,. \tag{122}$$

[14]Clearly, the value of the leading order coefficient $\omega_\mathrm{b}^{(1)}$ does not depend on the choice of coupling $g$, which is yet unspecified.

In the case of the GF coupling, the knowledge of the effective action is a crucial requirement to estimate the $\mathrm{O}(1/m)$ corrections to its large-mass limit. Following the discussion in ref. [24], we can in fact write,

$$\bar{g}^2_{\mathrm{GFT}}(\mu, M) \overset{M\to\infty}{=} \left[\bar{g}^{(0)}_{\mathrm{GFT}}(\mu)\right]^2 - \\ + \frac{\omega_{\mathrm{b}}(g_\star)}{m_\star} \int d^3x \left\langle \frac{t^2 E(t,y)}{\mathcal{N}} \left[\mathcal{O}_{\mathrm{b}}(0,\boldsymbol{x}) + \mathcal{O}_{\mathrm{b}}(T,\boldsymbol{x})\right] \right\rangle^{\mathrm{conn}}_{\mathrm{YM}} + \mathrm{O}\left(\frac{1}{m_\star^2}\right). \quad (123)$$

In the above equation $\langle \cdots \rangle^{\mathrm{conn}}_{\mathrm{YM}}$ stands for the connected expectation value in the Yang-Mills theory with action $S_{0,\mathrm{dec}}$, in the presence of homogeneous SF boundary conditions, and geometry $T = 2L$. The coupling $g_\star = \bar{g}_{\overline{\mathrm{MS}}}(m_\star)$ and quark mass $m_\star = \overline{m}_{\overline{\mathrm{MS}}}(m_\star)$ are those of the $N_{\mathrm{f}}$-flavour theory, while the energy density $E(t,y)$ is evaluated for $\sqrt{8t} = cL$ and $y_0 = T/2$ (cf. Sect. 6.1).

## 10.2 Leading order estimate of the O(1/$M$) corrections

We can obtain an estimate for the size of the $\mathrm{O}(1/m)$ term in eq. (123) using lattice simulations [24]. To this end, we define the difference,

$$\Delta(z) \equiv \bar{g}^2_{\mathrm{GFT}}(\mu_{\mathrm{dec}}, M) - [\bar{g}^{(0)}_{\mathrm{GFT}}(\mu_{\mathrm{dec}})]^2, \qquad z = M/\mu_{\mathrm{dec}}. \quad (124)$$

Given eq. (123) and the leading order (LO) result, eq. (122), for the matching coefficient $\omega_{\mathrm{b}}$, we can write the LO estimate for the $\mathrm{O}(1/M)$ corrections to the massive GFT coupling as,

$$\Delta(z)\big|_{\mathrm{LO}} \approx \frac{\omega^{(1)}_{\mathrm{b}} g_\star^2}{z}\left(\frac{M}{m_\star}\right) p_1\big(\bar{g}^2_{\mathrm{GFT}}(\mu_{\mathrm{dec}}, M)\big) + \mathrm{O}\left(g_\star^4, \frac{1}{z^2}\right). \quad (125)$$

Because $g_\star \to 0$ for $M \to \infty$, this estimate becomes more accurate the larger $M$ is, since eq. (122) becomes a better approximation to $\omega_{\mathrm{b}}$. The coefficient function $p_1$ is given by the matrix element,

$$p_1 \equiv -\lim_{a\to 0} \int d^3x \left\langle \frac{t^2 E(t,y)}{\mathcal{N}} L\big[\mathcal{O}_{\mathrm{b}}(0,\boldsymbol{x}) + \mathcal{O}_{\mathrm{b}}(T,\boldsymbol{x})\big] \right\rangle^{\mathrm{conn}}_{\mathrm{YM}}. \quad (126)$$

This must be computed, non-perturbatively, along a line of constant physics defined by a fixed value of $\bar{g}^{(0)}_{\mathrm{GFT}}(\mu_{\mathrm{dec}}) \equiv \bar{g}_{\mathrm{GFT}}(\mu_{\mathrm{dec}}, M)$.

For the lattice computation of $p_1$ we followed the strategy described in ref. [24]. We refer the interested reader to this reference for the details on the lattice set-up, and in particular for a discussion on the lattice discretization of the relevant operators. We performed simulations for $L/a = 10, 12, 16$, at 3 or 4 values of $\beta$ depending on the value of $L/a$. The values of $\beta$ were chosen in such a way that $p_1$ can be interpolated in $\beta$ so that the continuum limit can be taken at fixed $\bar{g}^{(0)}_{\mathrm{GFT}}(\mu_{\mathrm{dec}})$ corresponding to $\bar{g}_{\mathrm{GFT}}(\mu_{\mathrm{dec}}, M)$ with $z = 6$ and $c = 0.3, 0.36, 0.4$. The results for $p_1$ we obtained are

| $c$ | $\bar{g}^2_{\rm GFT}(\mu_{\rm dec}, M)$ | $L/a$ | $p_1$ |
|---|---|---|---|
| 0.3 | 4.448 | 10 | −0.396(62) |
| | | 12 | −0.370(73) |
| | | 16 | −0.305(49) |
| | | $\infty$ | −0.15(17) |
| 0.36 | 5.347 | 10 | −0.94(10) |
| | | 12 | −0.86(12) |
| | | 16 | −0.77(07) |
| | | $\infty$ | −0.48(26) |
| 0.42 | 6.690 | 10 | −2.06(17) |
| | | 12 | −1.88(20) |
| | | 16 | −1.71(12) |
| | | $\infty$ | −1.14(43) |

**Supplementary Table** 4: Results for $p_1$ at finite lattice spacing. Estimates of $a/L \to 0$ extrapolations linear in $a/L$ are also given.

collected in Table 4 for the 3 different lattice resolutions. We also provide an estimate of the continuum limit from linear extrapolations in $a/L$.

Given the non-perturbative results for $p_1$, all that is needed to compute $\Delta(z)|_{\rm LO}$ are the values for $g_\star$ and $M/m_\star$ at the relevant quark mass. The latter can be obtained once

$$\frac{M}{\Lambda^{(3)}_{\overline{\rm MS}}} = z \times \frac{\mu_{\rm dec}}{\Lambda^{(3)}_{\overline{\rm MS}}}\,, \tag{127}$$

is specified, thanks to the perturbative knowledge of the RG functions in the $\overline{\rm MS}$ scheme [40–42, 60–68]. Taking $\mu_{\rm dec}/\Lambda^{(3)}_{\overline{\rm MS}} \approx 2.3$ and $z = 6$, we find for $N_{\rm f} = 3$,

$$g^2_\star \approx 3.0277\,, \qquad M/m_\star \approx 1.4889\,, \tag{128}$$

where the 5-loop $\beta_{\overline{\rm MS}}$ and 4-loop $\tau_{\overline{\rm MS}}$ functions were used.

| $c$ | $\bar{g}^2_{\rm GFT}(\mu_{\rm dec}, M)$ | $\Delta(z=6)\vert_{\rm LO}$ |
|---|---|---|
| 0.30 | 4.448(14) | 0.0029 |
| 0.36 | 5.347(22) | 0.0075 |
| 0.42 | 6.690(37) | 0.0170 |

**Supplementary Table** 5: Results for $\Delta(z)|_{\rm LO}$ for $z = 6$ and different values of $c$.

In order to obtain a generous estimate for $\Delta(z)|_{\rm LO}$ we consider the value of $p_1$ at $L/a = 16$. This gives a more conservative choice than taking the continuum limit values (cf. Table 4). The results for $\Delta(z)|_{\rm LO}$ computed in this way are collected in Table 5. Comparing the results for $\Delta(z)|_{\rm LO}$ with the statistical uncertainties on the

corresponding massive coupling $\bar{g}^2_{\rm GFT}(\mu_{\rm dec}, M)$, we see that the former range from being about a factor 4-5 smaller for $c = 0.30$, to $\approx 3$ times smaller for $c = 0.36$, to $\approx 2$ times smaller for $c = 0.42$. We conclude that these effects can be safely discarded for our preferred choice, $c = 0.36$ and $z = 6$. They decrease further $\sim 1/z$ as we reach the decoupling limit.

# 11 Massive gradient flow coupling in perturbation theory

It is instructive to study the decoupling of heavy quarks at the lowest non-trivial order in perturbation theory. The discussion will give us some qualitative insight on the size of the leading corrections to the infinite quark-mass limit in computing the $\Lambda$-parameter via the decoupling method. Concomitantly, we hope that it will help the reader to better understand our strategy in simpler, perturbative, terms.

Unfortunately, there are no known perturbative results in QCD for the specific coupling definition we employed in our strategy, i.e. the GF coupling defined in a finite volume with SF boundary conditions; only the pure gauge part has been obtained so far [43]. There are, however, results for an infinite space-time volume [10, 69]. In the following we shall consider these in order to get a qualitative picture for our case. We note that in the limit where $c = \sqrt{8t}/L \to 0$, our coupling tends to the infinite volume one with corrections of $\mathrm{O}(c^4)$.

## 11.1 One-loop GF coupling in infinite volume

We begin by considering the perturbative results of ref. [69] for the energy density at positive flow time $\langle t^2 E(t)\rangle$ (cf. eq. (36) of that reference). After a proper normalization by an overall constant, we readily obtain the perturbative expansion of the GF coupling at the 1-loop order and for finite quark masses,

$$\bar{g}^2_{\rm GF}(\mu, \overline{m}(\mu)) = \bar{g}^2_s(\mu) + c_1(z)\bar{g}^4_s(\mu) + \mathrm{O}(\bar{g}^6_s)\,, \tag{129}$$

$$c_1(z) = (1.0978 + 0.0075\, N_{\rm f})/(4\pi) - \frac{N_{\rm f}}{24\pi^2}\Omega_1(z)\,, \tag{130}$$

where $\mu = 1/\sqrt{8t}$, $\bar{g}^2_s \equiv \bar{g}^2_{\overline{\rm MS}}(\mu)$, and $z \equiv \overline{m}(\mu)/\mu$. Note that we restrict to the case of $N_{\rm f}$ mass-degenerate quarks with renormalized quark mass $\overline{m}(\mu)$. At this order in perturbation theory there is no need to specify a renormalization scheme for the mass, which is therefore left unspecified.

The function $\Omega_1(z)$, which encodes the mass dependence of the coupling, is given here for completeness,

$$\Omega_1(z) = 1 - \gamma_{\rm E} - \ln(z^2/4) - z^2 + z^2 I(z)\,, \tag{131}$$

where

$$I(z) = \frac{1}{2}\int_0^\infty \mathrm{d}x\, e^{-x/4}\left(1+\frac{x}{4z^2}\right)\left(1-\frac{x}{2z^2}\right)\frac{u\ln u}{u^2-1}\,, \qquad u = \frac{\sqrt{x+4z^2}-\sqrt{x}}{\sqrt{x+4z^2}+\sqrt{x}}\,. \tag{132}$$

In the following, we will mostly be interested in its limits for small and large values of $z$,

$$\Omega_1(z) \quad \to \quad \begin{cases} -\frac{3}{2}\,z^2 + \mathrm{O}(z^4)\,, \\ -2\ln z - h - \frac{8}{5z^2} + \mathrm{O}(z^{-4})\,, \end{cases} \tag{133}$$

with $h = \gamma_\mathrm{E} + \frac{2}{3} - 2\ln 2$.

## 11.2 Renormalization group improved coupling in the one-loop model

Eq. (129) is a well behaved perturbative expansion for small $z$, where the coefficient function $c_1(z)$ remains finite. However, $c_1(z)$ diverges in the limit of large $z$ and the expansion becomes useless. Renormalization group (RG) improvement removes such an issue (and has already been silently used, setting $\mu = 1/\sqrt{8t}$), but for large $z$ a second scale appears and RG improvement is not generally applicable to our knowledge. One could resort to the effective theory at large $z$ at the expense of having to patch up domains of small and large $z$. Instead, here, we give a full RG improved expression valid for all $z$. Since it employs a non-systematic approximation, we call this the one-loop model.

We start from the truncated RG equations

$$\mu\frac{\mathrm{d}}{\mathrm{d}\mu}\frac{1}{\bar{g}^2_\mathrm{GF}} = 2b_0(N_\mathrm{f}) + \frac{N_\mathrm{f}}{24\pi^2}\left(\mu\frac{\mathrm{d}}{\mathrm{d}\mu}\right)\Omega_1(z)\,, \tag{134}$$

$$\mu\frac{\mathrm{d}}{\mathrm{d}\mu}\overline{m} = 0\,, \tag{135}$$

where $b_0(N_\mathrm{f})$ is the universal lowest order coefficient of the perturbative expansion of the $\beta$-function for $N_\mathrm{f}$-flavours. While in eq. (134) the one-loop term is taken along, in eq. (135) it is dropped. It only contributes via $c_1$ in eq. (129) and therefore is higher order, as we have remarked before. However, even in the massless case, one has to include the two-loop term in the $\beta$-function and the one-loop term in the mass anomalous dimension in order to get a systematic expansion of the $\Lambda$-parameter and the RGI mass in terms of the coupling, see eq. ([1].8,9) and eqs. (100),(101). We do not do this here and therefore we use the term model, not approximation.

The truncated RG equations can be integrated exactly to obtain,

$$\frac{1}{\bar{g}^2_\mathrm{GF}} = 2b_0(N_\mathrm{f})\ln\left(\mu/\Lambda^{(N_\mathrm{f})}_\mathrm{GF}\right) + \frac{N_\mathrm{f}}{24\pi^2}\Omega_1(z)\,, \quad \overline{m}(\mu) = M, \tag{136}$$

where $\Lambda_{\rm GF}^{(N_{\rm f})}$ is a conveniently chosen constant; taking $z \to 0$ (and $b_1 = d_0 = 0$ as fit for the model), we see that it is the $\Lambda$-parameter in the GF scheme for the $N_{\rm f}$-flavour theory. In the same way, the constant $M$ can be identified with the RGI mass or the running mass.

At large values of $z$, the quarks decouple. We thus expect that,

$$\frac{1}{\bar{g}^2_{\rm GF}} \overset{z\to\infty}{=} 2b_0(0)\ln\left(\mu/\Lambda^{(0)}_{\rm GF,dec}\right) + {\rm O}(1/z^2)\,, \tag{137}$$

for a properly chosen function $\Lambda^{(0)}_{\rm GF,dec} \equiv \Lambda^{(0)}_{\rm GF,dec}(\Lambda^{(N_{\rm f})}_{\rm GF}, M)$ of the $\Lambda$-parameter of the $N_{\rm f}$-flavour theory, $\Lambda^{(N_{\rm f})}_{\rm GF}$ , and of the RGI quark mass $M$. We call $\Lambda^{(0)}_{\rm GF,dec}$ the $\Lambda$-parameter of the effective theory.

Taking into account the asymptotic limit of $\Omega_1(z)$ for large $z$ (cf. eq. (133)),

$$\Omega_1(z) = -2\ln(z) - h + \Omega_1^{\rm sub}(z)\,, \quad \Omega_1^{\rm sub}(z) = {\rm O}(1/z^2)\,, \tag{138}$$

we find

$$\frac{1}{\bar{g}^2_{\rm GF}} \overset{z\to\infty}{=} 2b_0(N_{\rm f})\ln(\mu/\Lambda^{(N_{\rm f})}_{\rm GF}) - \frac{N_{\rm f}}{24\pi^2}(2\ln(z)+h) + {\rm O}(1/z^2) \equiv 2b_0(0)\ln(\mu/\Lambda^{(0)}_{\rm GF,dec})\,, \tag{139}$$

and with

$$b_0(N_{\rm f}) = b_0(0) - \frac{N_{\rm f}}{24\pi^2}\,, \tag{140}$$

we infer the relation between the $\Lambda$-parameter of the $N_{\rm f}$-flavour theory and that of the effective theory,

$$P_{\rm GF} \equiv \frac{\Lambda^{(0)}_{\rm GF,dec}}{\Lambda^{(N_{\rm f})}_{\rm GF}} = \left(\frac{M}{\Lambda^{(N_{\rm f})}_{\rm GF}}\right)^{\eta_0(N_{\rm f})} \exp\left(\frac{h\eta_0(N_{\rm f})}{2}\right)\,, \quad \eta_0(N_{\rm f}) = 1 - \frac{b_0(N_{\rm f})}{b_0(0)}\,. \tag{141}$$

This relation can also be obtained from the ratio of $\Lambda$-parameters in the $\overline{\rm MS}$-scheme, i.e. the function $P$ in eq. (51), setting $b_i = d_{i-1} = 0$, $i > 0$ and changing to the GF scheme for both $\Lambda^{(0)}$ and $\Lambda^{(3)}$. Note that this scheme-change is a $z$-independent factor.

### 11.3 Leading $\mathrm{O}(1/z^2)$ corrections to the decoupling relation in the model

We can gain some insight into the leading order corrections to the decoupling relation by keeping the ${\rm O}(1/z^2)$ terms in the large $z$ expansion of the function $\Omega_1(z)$ when establishing the relation eq. (139). Along the lines of our decoupling strategy we thus introduce effective $\Lambda$-parameters (cf. section 6.2), $\Lambda^{\rm eff} \equiv \Lambda^{(N_{\rm f}),{\rm eff}}_{\rm GF}$ and $\Lambda^{\rm eff}_{\rm dec} \equiv \Lambda^{(0),{\rm eff}}_{\rm GF,dec}$,

for the $N_\mathrm{f}$-flavour and effective theory respectively, through the definitions

$$\begin{aligned} \frac{1}{\bar{g}^2_\mathrm{GF}} &= 2b_0(N_\mathrm{f})\ln(\mu/\Lambda) - \frac{N_\mathrm{f}}{24\pi^2}(2\ln(z)+h-\Omega_1^\mathrm{sub}(z))\,, \\ &\equiv 2b_0(N_\mathrm{f})\ln(\mu/\Lambda^\mathrm{eff}) - \frac{N_\mathrm{f}}{24\pi^2}(2\ln(z)+h)\,, \\ &\equiv 2b_0(0)\ln(\mu/\Lambda^\mathrm{eff}_\mathrm{dec})\,, \end{aligned} \tag{142}$$

where for the ease of notation we also define $\Lambda \equiv \Lambda^{(N_\mathrm{f})}_\mathrm{GF}$ and $\Lambda_\mathrm{dec} \equiv \Lambda^{(0)}_\mathrm{GF,dec}$. First note that, analogously to $\Lambda$ and $\Lambda_\mathrm{dec}$, the effective $\Lambda$-parameters are related by (cf. eqs. (139)-(141))[15]

$$\Lambda^\mathrm{eff}_\mathrm{dec} = P_\mathrm{GF}(M/\Lambda^\mathrm{eff})\Lambda^\mathrm{eff} = (M/\Lambda^\mathrm{eff})^{\eta_0} e^{\frac{h\eta_0}{2}}\Lambda^\mathrm{eff}\,. \tag{143}$$

Second, by construction, in the limit $z\to\infty$, $\Lambda^\mathrm{eff}$ and $\Lambda^\mathrm{eff}_\mathrm{dec}$ approach their respective counterparts $\Lambda$ and $\Lambda_\mathrm{dec}$ with corrections of $\mathrm{O}(1/z^2)$. In particular, from the definitions in eq. (142), we have that,

$$\ln(\Lambda^\mathrm{eff}/\Lambda) = -\frac{\eta_0(N_\mathrm{f})}{2-2\eta_0(N_\mathrm{f})}\Omega_1^\mathrm{sub}(z)\,. \tag{144}$$

Considering the large $z$ expansion of $\Omega_1(z)$ in eq. (133), this gives

$$\frac{\Lambda^\mathrm{eff}}{\Lambda} = 1 + \frac{\eta_0(N_\mathrm{f})}{2-2\eta_0(N_\mathrm{f})}\frac{8}{5z^2} + \mathrm{O}(1/z^4) \overset{N_\mathrm{f}=3}{=} 1 + \frac{8}{45z^2} + \mathrm{O}(1/z^4)\,, \tag{145}$$

where the coefficient of the neglected $1/z^4$ term is of a similar size.

Considering that in our finite volume set-up $z = M/\mu = cML$, we find that, for $N_\mathrm{f}=3$, the above correction term is around 4% for $c=0.36$ and $ML=6$. Instead, if we use for $M$ in the model the value of $\overline{m}(\mu)$ in the non-perturbative computation and use the non-perturbative relation $M/\overline{m}_\mathrm{SF}(\mu_\mathrm{dec}) \approx 1.47$, eq. (118), the effect we find is about two times larger. This level of ambiguity should not be surprising given the model used in our calculation; we recall that the coupling is computed only at one-loop order in perturbation theory (in infinite volume), and the running of the quark mass is neglected all-together. Nonetheless, the model captures the right bulk part of the effect. Looking at figure, that shows our non-perturbative results for $\Lambda^\mathrm{eff}$, we see that for $c=0.36$ and $ML=6$ the correction is about 6% with respect to the infinite mass limit.

The result in eq. (145) also elucidates how the approach to the infinite mass limit can be tested when working at fixed $L$ by considering different values of $c$. In particular, larger values of $c$ are expected to lead to smaller corrections to the decoupling limit as

[15] In our strategy the $\Lambda$-parameters are first converted to the $\overline{\mathrm{MS}}$-scheme and the function $P_{\overline{\mathrm{MS}}}$ is used. However, since the ratio $P_\mathrm{GF}/P_{\overline{\mathrm{MS}}}$ is independent of $z$, this change of schemes is irrelevant.

they effectively correspond to larger ratios $M/\mu$.[16] Following this reasoning, our final determination of $\Lambda$ is based on the results for $c = 0.36$, which strikes a good balance between systematic and statistical uncertainties; both of them are small.

### 11.4 Illustration

The behavior of the coupling in the one loop model is shown in [1, Figure 3] (left) together with the massless coupling and the one in the decoupled theory with $\Lambda^{(0)}_{\mathrm{GF,dec}}$ from eq. (141). The figure further illustrates the trajectory that one follows in the non-perturbative decoupling strategy.

## References


[1] Dalla Brida, M. *et al.* High-precision calculation of the Quark-Gluon coupling from Lattice QCD (2025); *We refer to equation* (x) *in this main part of the paper as* ([1].x).

[2] Madras, N. & Sokal, A. D. The Pivot algorithm: a highly efficient Monte Carlo method for selfavoiding walk. *J. Statist. Phys.* **50**, 109–186 (1988).

[3] Wolff, U. Monte Carlo errors with less errors. *Comput. Phys. Commun.* **156**, 143–153 (2004). [Erratum: Comput.Phys.Commun. 176, 383 (2007)].

[4] Ramos, A. Automatic differentiation for error analysis of Monte Carlo data. *Comput. Phys. Commun.* **238**, 19–35 (2019).

[5] Virotta, F. *Critical slowing down and error analysis of lattice QCD simulations.* Ph.D. thesis, Humboldt-Universität zu Berlin, Mathematisch-Naturwissenschaftliche Fakultät I (2012).

[6] Dalla Brida, M. *et al.* A non-perturbative exploration of the high energy regime in $N_\mathrm{f} = 3$ QCD. *Eur. Phys. J. C* **78**, 372 (2018).

[7] Dalla Brida, M. *et al.* Results for $\alpha_s$ from the decoupling - strategy. *PoS* **LATTICE2021**, 492 (2022).

[8] Dalla Brida, M. & Ramos, A. The gradient flow coupling at high-energy and the scale of SU(3) Yang–Mills theory. *Eur. Phys. J. C* **79**, 720 (2019).

[9] Nada, A. & Ramos, A. An analysis of systematic effects in finite size scaling studies using the gradient flow. *Eur. Phys. J. C* **81**, 1 (2021).

[10] Lüscher, M. Properties and uses of the Wilson flow in lattice QCD. *JHEP* **08**, 071 (2010). [Erratum: JHEP 03, 092 (2014)].


[16] We recall that, instead, statistical uncertainties in $\bar{g}^2_{\mathrm{GF}}$ and thus $\Lambda^{\mathrm{eff}}$ tend to increase for larger $c$-values.

[11] Luscher, M. & Weisz, P. Perturbative analysis of the gradient flow in non-abelian gauge theories. *JHEP* **02**, 051 (2011).

[12] Bruno, M., Korzec, T. & Schaefer, S. Setting the scale for the CLS $2+1$ flavor ensembles. *Phys. Rev. D* **95**, 074504 (2017).

[13] Bruno, M. *et al.* QCD Coupling from a Nonperturbative Determination of the Three-Flavor $\Lambda$ Parameter. *Phys. Rev. Lett.* **119**, 102001 (2017).

[14] Ramos, A. & Sint, S. Symanzik improvement of the gradient flow in lattice gauge theories. *Eur. Phys. J. C* **76**, 15 (2016).

[15] Lüscher, M., Narayanan, R., Weisz, P. & Wolff, U. The Schrödinger Functional: a renormalizable probe for non-abelian gauge theories. *Nucl.Phys.* **B384**, 168–228 (1992).

[16] Sint, S. On the Schrödinger functional in QCD. *Nucl.Phys.* **B421**, 135–158 (1994).

[17] Gonzalez-Arroyo, A., Jurkiewicz, J. & Korthals-Altes, C. P. *Ground State Metamorphosis for Yang-Mills Fields on a Finite Periodic Lattice* (1987).

[18] Bode, A., Weisz, P. & Wolff, U. Two loop computation of the Schrodinger functional in lattice QCD. *Nucl. Phys. B* **576**, 517–539 (2000). [Erratum: Nucl.Phys.B 608, 481–481 (2001), Erratum: Nucl.Phys.B 600, 453–453 (2001)].

[19] Luscher, M., Sint, S., Sommer, R. & Weisz, P. Chiral symmetry and O(a) improvement in lattice QCD. *Nucl. Phys. B* **478**, 365–400 (1996).

[20] Fritzsch, P. & Ramos, A. The gradient flow coupling in the Schrödinger Functional. *JHEP* **1310**, 008 (2013).

[21] Dalla Brida, M. *et al.* Slow running of the Gradient Flow coupling from 200 MeV to 4 GeV in $N_\mathrm{f} = 3$ QCD. *Phys. Rev.* **D95**, 014507 (2017).

[22] Del Debbio, L., Panagopoulos, H. & Vicari, E. theta dependence of SU(N) gauge theories. *JHEP* **08**, 044 (2002).

[23] Schaefer, S., Sommer, R. & Virotta, F. Critical slowing down and error analysis in lattice QCD simulations. *Nucl. Phys. B* **845**, 93–119 (2011).

[24] Dalla Brida, M. *et al.* Determination of $\alpha_s(m_Z)$ by the non-perturbative decoupling method. *Eur. Phys. J. C* **82**, 1092 (2022).

[25] [RQCD 22] G. S. Bali *et al.* Scale setting and the light baryon spectrum in $\mathrm{N}_f = 2+1$ QCD with Wilson fermions. *JHEP* **05**, 035 (2023).

[26] Strassberger, B. *et al.* Scale setting for CLS 2+1 simulations. *PoS* **LATTICE2021**, 135 (2022).

[27] Aoki, Y. *et al.* FLAG Review 2021. *Eur. Phys. J. C* **82**, 869 (2022).

[28] d'Enterria, D. *et al.* The strong coupling constant: state of the art and the decade ahead. *J. Phys. G* **51**, 090501 (2024).

[29] Dalla Brida, M. *et al.* *Supplementary material* of The strength of the interaction between quarks and gluons (2025).

[30] Fritzsch, P., Heitger, J. & Kuberski, S. $\mathcal{O}(a)$ improved quark mass renormalization for a non-perturbative matching of HQET to three-flavor QCD. *PoS* **LATTICE2018**, 218 (2018).

[31] Sommer, R. *et al.* A strategy for B-physics observables in the continuum limit. *PoS* **LATTICE2023**, 268 (2024).

[32] Conigli, A. *et al.* $m_b$ and $f_{B^{(*)}}$ in $2+1$ flavour QCD from a combination of continuum limit static and relativistic results. *PoS* **LATTICE2023**, 237 (2024).

[33] Conigli, A. *et al.* B-physics observables in the continuum from a combination of static and relativistic results. *PoS* **EuroPLEx2023**, 005 (2024).

[34] Bruno, M. & Sommer, R. On fits to correlated and auto-correlated data. *Comput. Phys. Commun.* **285**, 108643 (2023).

[35] Dalla Brida, M. *et al.* Replication package 2501.06633. https://igit.ific.uv.es/alramos/replication-package-2501.06633 (2025).

[36] Dalla Brida, M. *et al.* Determination of the QCD Λ-parameter and the accuracy of perturbation theory at high energies. *Phys. Rev. Lett.* **117**, 182001 (2016).

[37] Dalla Brida, M. *et al.* Non-perturbative renormalization by decoupling. *Phys. Lett. B* **807**, 135571 (2020).

[38] Athenodorou, A. *et al.* How perturbative are heavy sea quarks? *Nucl. Phys. B* **943**, 114612 (2019).

[39] [ETM 21] C. Alexandrou *et al.* Ratio of kaon and pion leptonic decay constants with $N_f = 2+1+1$ Wilson-clover twisted-mass fermions. *Phys. Rev. D* **104**, 074520 (2021).

[40] Chetyrkin, K. G., Kühn, J. H. & Sturm, C. QCD decoupling at four loops. *Nucl. Phys.* **B744**, 121–135 (2006).

[41] Schröder, Y. & Steinhauser, M. Four-loop decoupling relations for the strong coupling. *JHEP* **01**, 051 (2006).

[42] Gerlach, M., Herren, F. & Steinhauser, M. Wilson coefficients for Higgs boson production and decoupling relations to $\mathcal{O}\left(\alpha_s^4\right)$. *JHEP* **11**, 141 (2018).

[43] Dalla Brida, M. & Lüscher, M. SMD-based numerical stochastic perturbation theory. *Eur. Phys. J. C* **77**, 308 (2017).

[44] Symanzik, K. Continuum Limit and Improved Action in Lattice Theories. 1. Principles and $\phi^4$ Theory. *Nucl. Phys. B* **226**, 187–204 (1983).

[45] Symanzik, K. Continuum Limit and Improved Action in Lattice Theories. 2. O(N) Nonlinear Sigma Model in Perturbation Theory. *Nucl. Phys. B* **226**, 205–227 (1983).

[46] Lüscher, M. and Weisz, P. On-Shell Improved Lattice Gauge Theories. *Commun. Math. Phys.* **97**, 59 (1985). [Erratum: Commun.Math.Phys. 98, 433 (1985)].

[47] Sheikholeslami, B. & Wohlert, R. Improved Continuum Limit Lattice Action for QCD with Wilson Fermions. *Nucl. Phys. B* **259**, 572 (1985).

[48] Balog, J., Niedermayer, F. & Weisz, P. Logarithmic corrections to O(a**2) lattice artifacts. *Phys. Lett. B* **676**, 188–192 (2009).

[49] Balog, J., Niedermayer, F. & Weisz, P. The Puzzle of apparent linear lattice artifacts in the 2d non-linear sigma-model and Symanzik's solution. *Nucl. Phys. B* **824**, 563–615 (2010).

[50] Husung, N., Marquard, P. & Sommer, R. Asymptotic behavior of cutoff effects in Yang-Mills theory and in Wilson's lattice QCD. *Eur. Phys. J. C* **80**, 200 (2020).

[51] Husung, N., Marquard, P. & Sommer, R. The asymptotic approach to the continuum of lattice QCD spectral observables. *Phys. Lett. B* **829**, 137069 (2022).

[52] Husung, N. Logarithmic corrections to O(a) and O($a^2$) effects in lattice QCD with Wilson or Ginsparg–Wilson quarks. *Eur. Phys. J. C* **83**, 142 (2023). [Erratum: Eur.Phys.J.C 83, 144 (2023)].

[53] Sint, S. & Sommer, R. The Running coupling from the QCD Schrodinger functional: A One loop analysis. *Nucl. Phys.* **B465**, 71–98 (1996).

[54] Dalla Brida, M. *et al.* Heavy Wilson quarks and O(a) improvement: nonperturbative results for $b_g$. *JHEP* **2024**, 188 (2024).

[55] Bruno, M., Finkenrath, J., Knechtli, F., Leder, B. & Sommer, R. Effects of Heavy Sea Quarks at Low Energies. *Phys. Rev. Lett.* **114**, 102001 (2015).

[56] Knechtli, F., Korzec, T., Leder, B. & Moir, G. Power corrections from decoupling of the charm quark. *Phys. Lett. B* **774**, 649–655 (2017).

[57] Navas, S. *et al.* Review of particle physics. *Phys. Rev. D* **110**, 030001 (2024).

[58] Campos, I. *et al.* Non-perturbative quark mass renormalisation and running in $N_f = 3$ QCD. *Eur. Phys. J. C* **78**, 387 (2018).

[59] Weinberg, S. Effective Gauge Theories. *Phys. Lett. B* **91**, 51–55 (1980).

[60] Bernreuther, W. & Wetzel, W. Decoupling of Heavy Quarks in the Minimal Subtraction Scheme. *Nucl. Phys.* **B197**, 228–236 (1982). [Erratum: Nucl. Phys.B513,758(1998)].

[61] Grozin, A. G., Hoeschele, M., Hoff, J. & Steinhauser, M. Simultaneous decoupling of bottom and charm quarks. *JHEP* **09**, 066 (2011).

[62] Kniehl, B. A., Kotikov, A. V., Onishchenko, A. I. & Veretin, O. L. Strong-coupling constant with flavor thresholds at five loops in the anti-MS scheme. *Phys. Rev. Lett.* **97**, 042001 (2006).

[63] van Ritbergen, T., Vermaseren, J. A. M. & Larin, S. A. The Four loop beta function in quantum chromodynamics. *Phys. Lett.* **B400**, 379–384 (1997).

[64] Czakon, M. The Four-loop QCD beta-function and anomalous dimensions. *Nucl. Phys.* **B710**, 485–498 (2005).

[65] Baikov, P. A., Chetyrkin, K. G. & Kühn, J. H. Five-Loop Running of the QCD coupling constant. *Phys. Rev. Lett.* **118**, 082002 (2017).

[66] Herzog, F., Ruijl, B., Ueda, T., Vermaseren, J. A. M. & Vogt, A. The five-loop beta function of Yang-Mills theory with fermions. *JHEP* **02**, 090 (2017).

[67] Luthe, T., Maier, A., Marquard, P. & Schroder, Y. The five-loop Beta function for a general gauge group and anomalous dimensions beyond Feynman gauge. *JHEP* **10**, 166 (2017).

[68] Chetyrkin, K. G., Falcioni, G., Herzog, F. & Vermaseren, J. A. M. Five-loop renormalisation of QCD in covariant gauges. *JHEP* **10**, 179 (2017). [Addendum: JHEP 12, 006 (2017)].

[69] Harlander, R. V. & Neumann, T. The perturbative QCD gradient flow to three loops. *JHEP* **06**, 161 (2016).
